# Effect of intravenous fluid volume on biomarkers of endothelial glycocalyx shedding and inflammation during initial resuscitation of sepsis

**DOI:** 10.1186/s40635-023-00508-4

**Published:** 2023-04-17

**Authors:** Stephen Macdonald, Erika Bosio, Gerben Keijzers, Sally Burrows, Moira Hibbs, Helen O’Donoghue, David Taylor, Ashes Mukherjee, Frances Kinnear, Lisa Smart, Juan-Carlos Ascencio-Lane, Edward Litton, John Fraser, Nathan I. Shapiro, Glenn Arendts, Daniel Fatovich, The REFRESH Trial Investigators, David McCutcheon, Anton Leonard, Jonathan Burcham, Rinaldo Bellomo, Glenn Arendts, Edward Litton, Amanda Harley, James Winearls, Juan Carlos Ascencio-Lane, Simon Brown, David Cooper, Daniel Fatovich, Ioana Vlad, Bradley Wibrow, Matthew Anstey, Sarah Hazelwood

**Affiliations:** 1 Centre for Clinical Research in Emergency Medicine, Harry Perkins Institute of Medical Research, Perth, WA Australia; 2 Medical School, University of Western Australia, Perth, WA Australia; 3 Emergency Department, Royal Perth Hospital, Perth, WA Australia; 4 Emergency Department, Gold Coast University Hospital, Gold Coast, QLD Australia; 5 Faculty of Health Sciences and Medicine, Bond University, Gold Coast, QLD Australia; 6 School of Medicine, Griffith University, Gold Coast, QLD Australia; 7 Research Foundation, Royal Perth Hospital, Perth, WA Australia; 8 Research Centre, Royal Perth Hospital, Perth, WA Australia; 9 Emergency Department, Austin Health, Melbourne, Australia; 10 Emergency Department, Armadale Health Service, Perth, WA Australia; 11 Department of Medicine, University of Queensland, Brisbane, QLD Australia; 12 School of Science, Health Engineering and Education, Murdoch University, Perth, WA Australia; 13 Emergency Department, Royal Hobart Hospital, Hobart, TAS Australia; 14 Intensive Care, Fiona Stanley Hospital, Perth, WA Australia; 15 Critical Care Research Group, The Prince Charles Hospital, University of Queensland, Brisbane, QLD Australia; 16 Department of Emergency Medicine, Beth Israel Deaconess Medical Center and Harvard Medical School, Boston, MA USA; 17 Emergency Department, Fiona Stanley Hospital, Perth, WA Australia

**Keywords:** Sepsis, Fluid therapy, Endothelium, Endothelial glycocalyx, Inflammation

## Abstract

**Purpose:**

To investigate the effect of IV fluid resuscitation on endothelial glycocalyx (EG) shedding and activation of the vascular endothelium and inflammation.

**Materials and methods:**

A planned biomarker sub-study of the REFRESH trial in which emergency department (ED) patients) with suspected sepsis and hypotension were randomised to a restricted fluid/early vasopressor regimen or IV fluid resuscitation with later vasopressors if required (usual care). Blood samples were collected at randomisation (T0) and at 3 h (T3), 6 h (T6)- and 24 h (T24) for measurement of a range of biomarkers if EG shedding, endothelial cell activation and inflammation.

**Results:**

Blood samples were obtained in 95 of 99 enrolled patients (46 usual care, 49 restricted fluid). Differences in the change in biomarker over time between the groups were observed for Hyaluronan (2.2-fold from T3 to T24, *p* = 0.03), SYN-4 (1.5-fold from T3 to T24, P = 0.01) and IL-6 (2.5-fold from T0 to T3, *p* = 0.03). No difference over time was observed between groups for the other biomarkers.

**Conclusions:**

A consistent signal across a range of biomarkers of EG shedding or of endothelial activation or inflammation was not demonstrated. This could be explained by pre-existing EG shedding or overlap between the fluid volumes administered in the two groups in this clinical trial.

*Trial registration* Australia New Zealand Clinical Trials Registry ACTRN126160000006448 Registered 12 January 2016.

**Supplementary Information:**

The online version contains supplementary material available at 10.1186/s40635-023-00508-4.

## Introduction

Sepsis is defined as life-threatening organ dysfunction due to a dysregulated response to infection, with septic shock being a subset of sepsis in which underlying circulatory and cellular/metabolic abnormalities are profound enough to substantially increase mortality [1]. The Surviving Sepsis Campaign suggest initial resuscitation with at least 30 ml/kg of isotonic crystalloid fluid to optimise cardiac output and restore perfusion in septic shock, although this is based upon consensus rather than high-level evidence [2]. In the past decade evidence has emerged which questions the effectiveness and safety of this approach [3–6]. Some have promoted more sparing use of fluids and earlier introduction of vasopressor medications to restore perfusion [7]. The resulting uncertainty has led to substantial practice variation [8].

In a landmark clinical trial of fluid bolus therapy in children with hypoperfusion in resource-poor settings in Africa, the excess mortality in those children treated with fluids was found to be due to cardiovascular collapse [9]. This finding is consistent with a preclinical trial in an experimental ovine sepsis model, where initial fluid resuscitation resulted in an increased subsequent requirement for vasopressors [10]. One suggested mechanism for harm with IV fluids is an impact on the endothelial surface layer due to shedding of the endothelial glycocalyx (EG) and consequently propagation of systemic inflammation [11, 12]. Although studying the EG in vivo is challenging, measurement of the soluble components of the EG such as the proteoglycan Syndecan molecules and glycosaminoglycans such as Hyaluronan and Heparan Sulphate in blood may be used as a surrogate indicator for the extent of EG shedding [13]. Clinical studies in sepsis have demonstrated variable associations between IV fluid volume and the level of biomarkers of EG shedding [14–17]. Furthermore, studies in healthy subjects and perioperative settings may not be relevant to critical illnesses, such as sepsis, where EG shedding is already occurring [18].

The Restricted Fluid Resuscitation in Sepsis-associated Hypotension (REFRESH) trial was a multicentre randomised trial designed to determine the feasibility of comparing a fluid-restricted/early vasopressor regimen compared to usual care to achieve perfusion targets during the early resuscitation of patients with suspected septic shock in the emergency department [19]. The aim of this planned sub-study was to test the hypothesis that IV fluid is a mediator of EG shedding, endothelial cell activation and inflammation during the resuscitation phase of septic shock [20].

## Methods

### Participants and setting

**Fig. 1:**
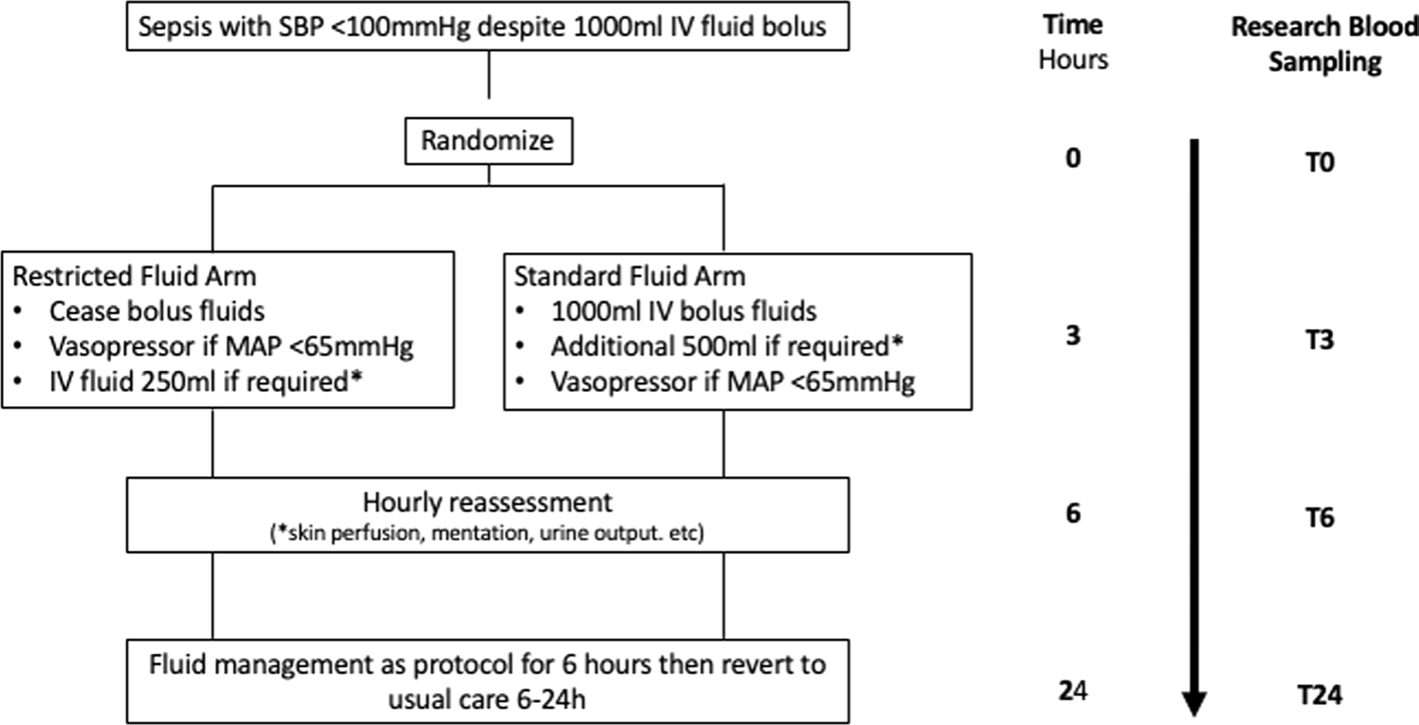
Flowchart summarising trial interventions

The methods for the REFRESH trial have previously been described [19, 20]. Briefly, patients presenting to the emergency department (ED) of eight Australian hospitals with clinically suspected infection who had a systolic blood pressure (SBP) of < 100 mmHg despite a minimum of 1000 ml crystalloid fluids were randomised to either (1) the early commencement of a vasopressor infusion with limited IV fluid boluses administered for defined indications (restricted fluids) or (2) additional IV fluid boluses with later introduction of vasopressors, if required, to achieve the clinically desired blood pressure target (usual care). The haemodynamic resuscitation protocol was followed for the first 6 h and up to 24 h for those admitted to the intensive care unit (ICU). A summary of the trial interventions and procedures is shown in Fig. 1.

### Research samples

Research blood sampling was performed at randomisation and at 3, 6 and at 24 h later (T0, T3, T6 and T24). Following collection, samples were processed within 2 h with serum collected by centrifugation at 3000 RPM for 10 min, followed by storage in 0.5 ml aliquots at − 80 °C. Samples were subsequently transported for batch analysis at the laboratory of the Centre for Clinical Research in Emergency Medicine in Perth, Australia. We selected the following biomarkers based upon prior work by our group and others. EG shedding: Syndecan 1 (SYN-1), Syndecan 4 (SYN-4), Hyaluronan, Heparan Sulphate. Endothelial Cell activation: Intercellular Adhesion Molecule (ICAM), Vascular Cell Adhesion Molecule (VCAM), E-Selectin, Vascular Endothelial Growth Factor Receptor-1 (VEGFR-1). Cardiac stretch: Pro-Atrial Natriuretic Peptide (Pro-ANP); Renal Injury: Neutrophil Gelatinase-Associated Lipocalin (NGAL); Systemic inflammation: Interleukin (IL)-6, IL-10, Resistin. Plasma biomarker concentrations were determined by enzyme-linked immunosorbent assay (ELISA) and Cytometric Bead Array (CBA) techniques. Heparan Sulfate was measured in plasma using a fully validated, commercial kit, with a lower limit of detection of 0.19 ng/ml (Elabscience, TX, USA). ICAM, VCAM, E-Selectin, IL-10 and IL-6 were measured in a multiplex CBA format using Flex sets (BD Biosciences, CA, USA). All remaining biomarkers were measured using DuoSet ELISA kits (R&D Systems, MN, USA), and individually optimised prior to use to achieve average intra-plate coefficients of variation below 10%.

### Statistical analyses

Biomarkers were analysed by group allocation according to intention to treat. Normality of distribution was assessed by inspection of box and whisker plots of values within assay limits by group at each timepoint. All variables demonstrated departures from normality and were subsequently log-transformed for analysis. An interaction of group and time was used in linear mixed models to assess whether the change over time in each marker differed between the groups. For biomarkers, where some values lay outside the limit of detection for the assay, random effects Tobit regression was used. These analysis techniques retain patients without biomarker data at all timepoints in the analysis by utilising maximum likelihood estimation (MLE). MLE uses all available information to calculate the most likely parameter estimates at the sample level as opposed to imputing values at the individual level. Results are reported as fold changes after exponentiating coefficients derived on the log scale. A per-protocol analysis was also undertaken excluding those cases in which there was a deviation from protocol regarding the recommended fluid volumes in each arm. As an exploratory study, no adjustment was made for multiple testing and interactions with *p* values < 0.1 were considered to provide sufficient evidence to warrant further consideration. In this instance, Bootstrapping was employed to investigate sensitivity to sampling variation.

## Results

The REFRESH trial enrolled a total of 99 participants randomised to one of the two treatment arms [19]. Mortality at 90 days was 4/48 (8%) and 3/47 (6%) in the restricted and usual care groups, respectively (two from each group were lost to follow up). Four participants had no research blood sampling, three of which were in the usual care group and one in the restricted fluids group leaving a total of 95 (46 usual care and 49 restricted fluid) in this analysis. Of the 95 cases in the sub-study, 68 (72%) had complete sampling at all four timepoints, 14 (15%) were sampled at 3 timepoints, 12 (13%) at two timepoints and one (1%) at one timepoint. The baseline characteristics of participants are shown in Table 1. Table 2 shows the volume of IV fluids and use of vasopressors over the first 24 h of care. The haematocrit measured at T24 was 0.34 ± 0.06 in the usual care group and 0.34 ± 0.05 in the restricted group, *p* = 0.7. Table 3 shows the results of the biomarker analyses.

**Table 1:** Baseline characteristics of study participants at randomisation

	Usual care *N* = 46	Restricted volume *N* = 49
Age (years)	66 (45, 76)	65 (52, 78)
Male sex n (%)	29 (63)	30 (61)
Weight (kg)	72 (64, 92)	80 (66, 88)
Mean temperature (°C)	37.5 ± 1.2	37.3 ± 1.3
Mean heart rate (beats/min)	96 ± 20	96 ± 21
Mean respiratory rate (breaths/min)	23 ± 6	22 ± 5
SpO_2_ (%)	96 (95, 98)	96 (94, 98)
FiO_2_	0.21 (0.21, 0.32)	0.21 (0.21, 0.3)
GCS	15 (15,15)	15 (15, 15)
Mean SBP (mmHg)	87 ± 9	86 ± 9
Mean MAP (mmHg)	64 ± 8	65 ± 7
Lactate (mmol/L)	1.95 (1.25, 2.85)	1.7 (1.1, 3.5)
Charlson score	2 (0, 5)	2 (1, 4)
APACHE II score	14 (10, 19)	15 (11, 20)
SOFA score	5 (4, 7)	5 (3, 9)
Non-CVS SOFA score	3 (2, 5)	3 (1, 6)
Creatinine (μmol/L)	130 (80, 173)	112 (76, 160)
Haematocrit T0	0.38 ± 0.07	0.38 ± 0.06
Infection source *N* (%)		
Respiratory	18 (39)	13 (27)
Urinary	8 (17)	16 (33)
Skin/soft tissue	6 (13)	6 (12)
Bloodstream	7 (15)	3 (6)
Abdominal/pelvis	2 (5)	5 (10)
Other/unidentified	5 (11)	6 (12)
Pre randomisation fluid volume (ml)	1250 (1000, 2000)	1400 (1000, 1500)
Time from ED arrival (minutes)	143 (89, 250)	140 (103, 214)

Data are medians (Q1, Q3) unless stated otherwise. *SBP* systolic blood pressure, *SpO*_*2*_ peripheral oxygen saturations, *FiO*_*2*_ fractional inspired oxygen concentration, *GCS* Glasgow Coma Scale, *APACHE* Acute Physiology and Chronic Health Evaluation, *SOFA* Sequential Organ Failure Assessment, *Non-CVS SOFA* total SOFA score minus cardiovascular domain

**Table 2:** Fluid and vasopressor use

	Usual care *N* = 46	Restricted volume *N* = 49	P value
*Fluid volume*			
T0–T6 (ml) total	1685 (1017, 2500)	1000 (625, 1458)	< 0.001
T0–T6/kg (ml)	23 (15, 33)	12 (7, 20)	< 0.001
Total prerandomisation-T6	3000 (2550, 3900)	2400 (1860, 2750)	< 0.001
Total to T6/kg (ml)	43 (35, 49)	31 (23, 39)	< 0.001
T6–T24 (ml)	1060 (428, 2166)	1145 (500, 2000)	0.84
Total prerandomisation-T24 (ml)	4360 (3350, 5252)	3550 (2750, 4410)	0.008
Total to T24/kg (ml)	61 (46, 79)	40 (31, 64)	0.005
*Vasopressor use N (%)*	**26 (53)**	**39 (78)**	
Vasopressor in ED *N* (%)	23 (50)	36 (73)	0.018
Vasopressor at 24 h *N*	19 (41)	24 (49)	0.45
Time to start vasopressor (mins):			
From ED arrival	250 (168, 483)	223 (127, 316)	0.12
From randomisation	150 (63, 224)	34 (15, 88)	0.001
Type of vasopressor:			
Noradrenaline *N* (%)	23 (50)	30 (60)	0.33
Metaraminol only *N* (%)	3 (6)	9 (18)	
Volume prior to vasopressor (ml)	2000 (2000, 2777)	1400 (1000, 1700)	< 0.001
Duration of vasopressor (h)	33 (15, 50)	21 (9, 42)	0.13
Peak vasopressor dose	0.18 (0.1, 0.43)	0.11 (0.08, 0.22)	0.14
Mean MAP T0–T6 (mmHg)	71 ± 6	73 ± 6	0.21

Data are medians (Q1, Q3) unless stated otherwise. *P* values calculated using Wilcoxon rank-sum test for continuous variables and Fishers exact test for categorical variables. Peak vasopressor dose mcg/kg/min of noradrenaline (or equivalent)

**Table 3:** Biomarker values

	Standard			Restricted		
	*N*	Mean (SD)	Median (Q1, Q3)	*N*	Mean (SD)	Median (Q1, Q3)
*ProANP (ng/ml)*						
T0	44	35 (27)	26 (17, 46)	46	41 (29)	33 (20, 58)
T3	44	31 (23)	24 (14, 44)	43	34 (30)	23 (14, 46)
T6	39	25 (19)	32 (18, 55)	42	27 (21)	23 (11, 34)
T24	39	35 (21)	23 (14, 42)	45	32 (18)	30 (16, 47)
*Syndecan-1 (ng/ml)*						
T0	44	19.0 (26.7)	7.0 (5.0, 20.2)	46	19.5 (36.4)	5.5 (3.2, 16.8)
T3	44	19.3 (32.3)	6.9 (3.9, 18.4)	44	20.6 (37.8)	5.9 (2.8, 15.1)
T6	38	17.0 (23.2)	5.9 (3.9, 18)	41	18.7 (41.3)	5.1 (2.8, 10.8)
T24	39	17.9 (28.0)	7.6 (5.1, 12.9)	44	25.0 (46.8)	6.9 (4.7, 15.5)
*Syndecan-4 (pg/ml)*						
T0	44	2853 (5595)	804 (510, 2136)	46	3189 (7021)	868 (483, 2156)
T3	44	3439 (6410)	1195 (502, 2388)	44	3773 (11,950)	826 (449, 1918)
T6	39	2407 (4030)	756 (411, 1628)	42	2494 (5723)	602 (361, 2134)
T24	39	2144 (4462)	542 (283, 1281)	45	3301 (8633)	776 (324, 1714)
*Hyaluronan (ng/ml)*						
T0	44	920 (1688)	216 (73, 1123)	45	1046 (2700)	261 (74, 702)
T3	44	930 (2700)	144 (80, 543)	44	508 (1614)	152 (40, 377)
T6	39	671 (2096)	136 (58, 372)	42	561 (2249)	121 (33, 269)
T24	39	1397 (6422)	124 (36, 230)	45	978 (3217)	172 (43, 515)
*Hep Sulphate (ng/ml)*						
T0	44	2650 (1687)	2264 (1481, 3361)	46	3123 (2265)	2248 (1601, 3853)
T3	44	2737 (1651)	2142 (1368, 4093)	44	2819 (1790)	2141 (1522, 4048)
T6	39	3028 (2711)	2064 (1381, 4048)	42	2995 (2335)	2118 (1138, 4536)
T24	39	2683 (1608)	2244 (1384, 3691)	45	3020 (1891)	2264 (1505, 4183)
*ICAM-1 (pg/ml)*						
T0	44	1070 (953)	681 (456, 1272)	46	1179 (949)	856 (615, 1476)
T3	44	912 (748)	608 (451, 1070)	44	1004 (948)	633 (440, 1063)
T6	39	1332 (1855)	811 (480, 1362)	42	1401 (1381)	1150 (637, 1617)
T24	39	1075 (1088)	598 (361, 1363)	45	1191 (1251)	690 (326, 986)
*VCAM-1 (pg/ml)*						
T0	44	3348 (2509)	3075 (1210, 4844)	46	3751 (2713)	3066 (1858, 4780)
T3	44	2781 (2905)	1745 (923, 3827)	44	2751 (2153)	2007 (1071, 4196)
T6	39	3913 (3936)	2235 (1680, 4765)	42	4581 (3361)	3549 (2345, 5830)
T24	39	2664 (3158)	1354 (920, 3618)	45	2785 (4209)	1176 (570, 2718)
*E Selectin (ng/ml)*						
T0	44	49 (47)	30 (12, 87)	47	62 (71)	32 (13, 104)
T3	43	44 (38)	31 (16, 59)	41	55 (60)	31 (12, 89)
T6	39	47 (40)	34 (12, 74)	42	55 (55)	27 (10, 111)
T24	39	36 (27)	30 (14, 65)	45	45 (48)	23 (11, 66)
*Resistin (ng/ml)*						
T0	44	140 (135)	103 (55, 146)	46	122 (205)	73 (31, 122)
T3	44	197 (327)	118 (60, 164)	44	177 (324)	104 (41, 171)
T6	39	137 (142)	101 (55, 151)	41	154 (253)	107 (38, 188)
T24	39	128 (157)	74 (35, 162)	45	133 (165)	90 (37, 139)
*NGAL (pg/ml)*						
T0	44	501 (630)	286 (137, 517)	46	539 (641)	354 (158, 661)
T3	44	395 (462)	262 (155, 468)	43	397(405)	242 (137, 517)
T6	38	359 (279)	283 (145, 493)	42	615 (816)	341 (148, 594)
T24	38	369 (366)	230 (161, 486)	45	431 (447)	281 (151, 540)
*IL-6 (ng/ml)*						
T0	44	58.7 (164.1)	1.6 (0.3, 21.9)	46	50.1 (146.7)	9.3 (2.9, 13.1)
T3	43	15.8 (38.0)	1.0 (0.3, 8.1)	42	13.6 (45.5)	2.9 (0.1, 18.1)
T6	39	4.9 (10.5)	0.8 (0.1, 4.5)	42	12.2 (41.4)	3.3 (0.1, 16.6)
T24	39	0.7 (1.5)	0.1 (0.05, 4.5)	45	5.5 (11.2)	0.1 (0.02, 0.4)
*IL-10 (ng/ml)*						
T0	42	254 (1386)	3.5 (0.13, 54)	45	265 (796)	0.13 (0.13, 62)
T3	43	159 (591)	11.3 (0.13, 67)	41	131 (492)	0.13 (0.13, 38)
T6	37	42 (64)	15.3 (0.13, 39)	39	97 (232)	7.87 (0.13, 84)
T24	38	2.9 (8.5)	0.13 (0.13, 0.13)	44	128 (495)	0.13 (0.13, 38)

### Endothelial glycocalyx markers

A significant difference in Hyaluronan levels over time was detected (interaction term *p* = 0.04), driven by opposing slope directions between the two groups for T3 to T24 and T6 to T24. This resulted in a 2.2-fold change in slope for T3 to T24 (*p* = 0.03) and a 2.6-fold change for T6 to T24 (*p* = 0.01) for the restricted fluid group compared to usual care.

**Fig. 2:**
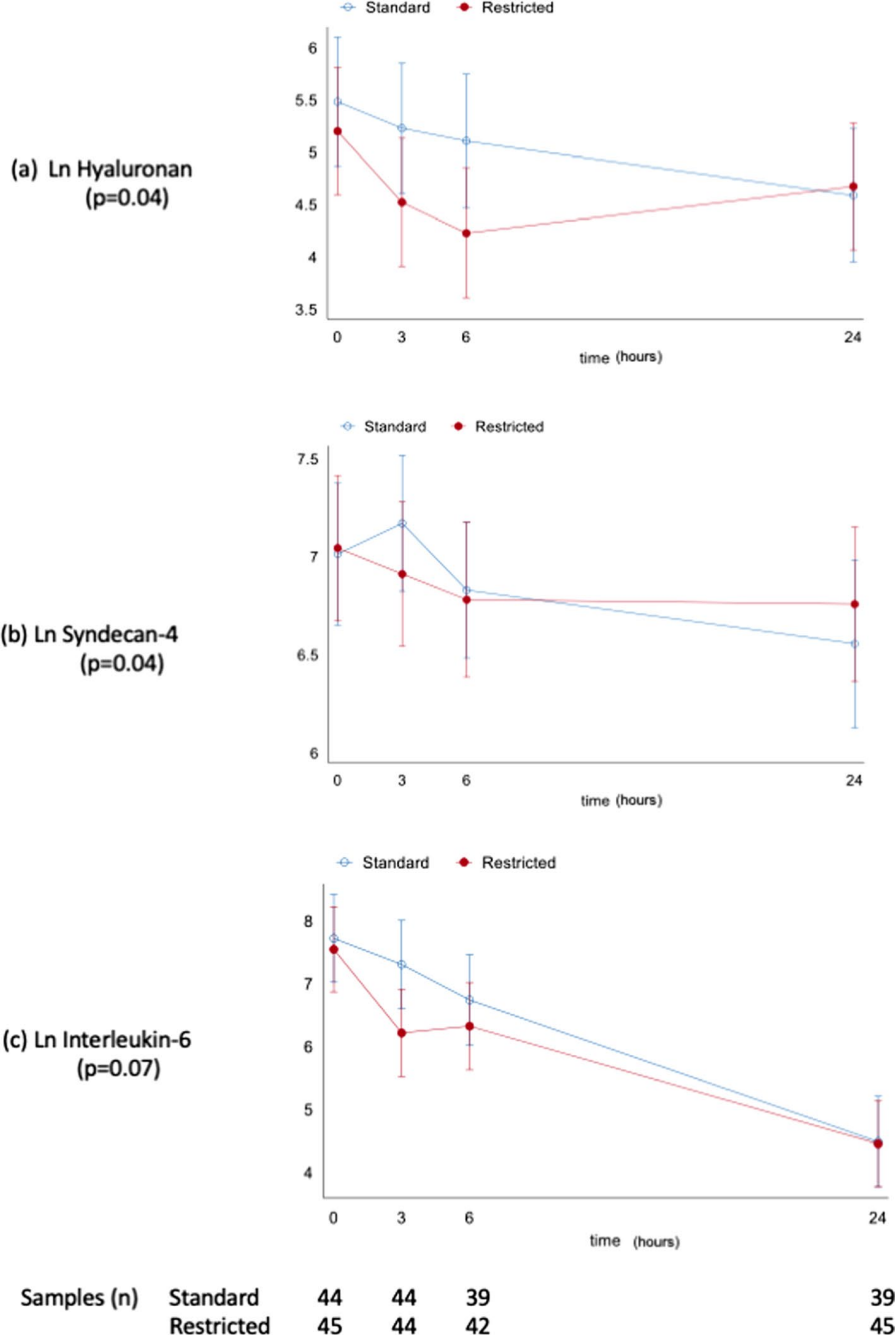
Log (Ln) linear predicted means (with 95% confidence intervals) against time (h) for Hyaluronan, Syndecan-4 and Interleukin-6 for usual (standard) care and restricted fluid resuscitation. *P* values for interaction term for group*fluid; See Additional files 1 and 2 for details of analysis

There were no differences in absolute values at the T0 and T24 timepoints (Fig. 2a). For SYN-4 there was a significant difference over time between the groups (Interaction term *p* value 0.04) driven by a greater negative slope in the restricted fluid group compared to usual care, equating to a 1.6-fold change in slope between T3 and T24, *p* = 0.007 (Fig. 2b). For SYN-1 and Heparan Sulphate, no difference over time between the groups was found (interaction term *p* values 0.92 and 0.72, respectively).

### Natriuretic peptide

There was no difference between the groups in the change over time for the natriuretic peptide Pro-ANP (interaction term *p* value 0.31).

### Endothelial cell activation markers

No differences between the groups were identified in the pattern over time of any biomarker associated with activation of endothelial cells. These were ICAM (*p* = 0.31), VCAM (*p* = 0.26), E-Selectin (*p* = 0.73), and VEGFR-1 (0.96).

### Inflammatory and other biomarkers

For the cytokine IL-6, the pattern over time differed between the groups (interaction term *p* = 0.07) driven by a greater negative slope in the restricted fluids group (2.5-fold change in slope compared to usual care) between T0 and T3 (*p* = 0.03) (Fig. 2c). There were no significant differences between the groups in IL-10 (*p* = 0.22), Resistin (*p* = 0.43) and the renal injury biomarker NGAL (*p* = 0.15) over time.

A sensitivity analysis ‘per-protocol’ excluded six cases in the usual care group who did not receive the minimum recommended fluid volume of at least 1 L post randomisation and one case in the restricted fluid group who received more than the permitted fluid volume of 1.25 L in the first 6 h post randomisation. This did not alter the results of the primary analysis (data not shown). Bootstrapping of the analyses, where the interaction term *p* value was < 0.1 also did not alter the results. Further details of the results including boxplots for each biomarker by group at each timepoint is given in the supplementary appendix. Similarly, analysis of the EG biomarkers adjusted for pre-randomisaiton fluid volume did not yield different results.

## Discussion

In this planned sub-study of the REFRESH trial, differences over time in the pattern of Hyaluronan, SYN-4 and IL-6 were observed during the first 24 h of care driven by greater or contrasting downward patterns in the restricted volume group involving the T3 and T6 timepoints. No differences were noted for other biomarkers of EG shedding, SYN1, Heparan Sulphate, nor for Pro-ANP, IL-10 and any biomarkers related to endothelial cell activation.

The question of the optimal approach to haemodynamic resuscitation has been identified as one of the top priorities for research in septic shock [21]. The conventional approach, supported by consensus guidelines, involves initial resuscitation with at least 30 ml/kg of crystalloid fluid, although this is not based upon high level clinical trial evidence [22]. Potential harm associated with IV fluids may be due to tissue oedema in the setting of increased capillary leakiness in sepsis [23]. Specifically, there has been a focus on how IV fluids may interact with the EG layer in the setting of critical illness [24]. Understanding the pathobiological mechanisms underpinning the effect of IV fluids is relevant to inform future trials focused on patient centred outcomes.

Previous studies have found evidence supporting an effect of IV fluids on the EG in sepsis. Pouska et al. demonstrated a persistent change in the perfusion boundary ratio on sublingual intravital microscopy, a surrogate measure of EG thickness, among 16 patients with sepsis undergoing fluid bolus administration [25]. Smart et al. found an association between fluid volume and hyaluronan during the first 3 h of resuscitation, although no relationship was seen for SYN-1 or SYN-4 [16]. Hippensteel et al. reported an association between Heparan Sulphate and the volume of IV fluids at 6 h [14]. Finally, in a preclinical ovine endotoxic shock model animals randomised to fluid resuscitation had a more rapid rate of rise in Hyaluronan compared to animals that did not, although the overall peak did not differ. It is noted, however, that Hyaluronan elevation in response to fluid resuscitation may be a reflection of return of hyaluronan to the circulation from interstitial fluid rather than shed from the EG [13].

In a multicentre study of 619 patients with sepsis, there was no association between fluid volume and SYN-1 on the first day of admission, although SYN-1 was associated with illness severity and mortality [26]. In a prospective study, Puskarich et al. found that elevated SYN-1 was associated with mortality but there was no relationship with the volume of fluid administered [27]. However, Saoraya et al. reported that SYN-1 measured at the time of admission was associated with subsequent fluid requirements as well as mortality, raising the question of whether the association was due to sicker patients requiring a greater volume of fluid [28]. In a randomised trial of a liberal versus restricted fluid resuscitation, the same authors measured SYN-1 levels at 6 h finding a geometric mean ratio of 0.82 (95% CI 0.66–1.020, *p* = 0.07), in favour of the restricted fluid regimen [15]. In our study, no relationship between fluid volume and SYN-1 was identified. Our finding of a difference in the pattern of SYN-4 between the groups is of interest, although SYN-4 has been relatively less studied as a biomarker of EG shedding [29]. The lack of an associated difference in SYN-1 or HS, however, increases the likelihood that this may be due to chance.

Overall, we found limited evidence for an effect of fluids on EG shedding. In support of the hypothesis are temporal changes in some biomarkers coinciding with the maximal difference in fluid volume during the first 6 h of treatment. Against is the lack of consistency across all EG and inflammatory markers, and the lack of any effect on changes in the endothelial activation biomarkers. Though there was a 20 ml/kg separation in fluid volume between the two study arms at 24 h, this difference may have been insufficient to yield a consistent effect on EG shedding. This is supported by there being no difference in Pro-ANP levels between the groups. In a preclinical endotoxic shock model, ANP was significantly higher in the fluid group [10]; however, Hippensteel et al. did not detect any association between ANP and fluid volume among patients with sepsis [14]. This is relevant, because one postulated mechanism of EG shedding due to fluid administration is the activation of matrix metalloproteinases (MMPs) by natriuretic peptides [30, 31]. In addition, we observed no difference between groups for markers of endothelial cell activation. The endothelium is considered to be a player in the pathogenesis of sepsis [12]. One suggested cause of harm with exogenous fluids in sepsis is oedema from increased vascular permeability due to endothelial activation in the setting of EG shedding [23], although recent experimental evidence has challenged this concept [32, 33].

Of some interest is the pattern of IL-6 with a steeper decline in the restricted fluids group which coincided with the period of maximal difference in fluid volume. As an acute phase pro-inflammatory cytokine IL-6 displayed a typical pattern of decline over the first 24 h [34]. The temporal pattern was similar to that of Hyaluronan which is itself a pro-inflammatory mediator [35].

### Limitations

In critical illness, where EG shedding and inflammation are already established, identifying a consistent response to an intervention is challenged by the confounding effects of illness severity, the clinical heterogeneity of sepsis and the timing of presentation to hospital. In this pilot clinical trial set in the ED the overall severity of illness was lower compared to other sepsis studies. As a pragmatic clinical trial there were several protocol deviations which potentially impacted on separation; however, a ‘per-protocol’ analysis did not yield different results from the intention to treat analysis. Importantly, participants received at least 1 L of fluid prior to randomisation (Table 1) which means fluid mediated EG shedding may have occurred prior to T0 and early signals may have been missed, and subsequent sample collection was related to time rather than fluid volume administered. We undertook a sensitivity analysis adjusting for baseline fluid volume did not alter the results for the EG biomarkers. The limitations of serum biomarkers as measures of EG shedding has previously been described [18]. In vitro assessment of EG thickness is an alternative approach which should be considered in future studies.

One issue is the question of whether haemodilution may mask as significant difference in biomarkers measured between groups receiving different volumes of fluid. Some authors have adjusted biomarker results for albumin [11] or haemoglobin [36], although this is not standard. Uncertainty about the pharmacokinetics of these molecules means the validity of such adjustments is uncertain [18]. Of note, while haematocrit reduced by a similar amount in the first 24 h in both groups, there was no difference between the groups at T24, although this was not measured at the intervening timepoints. Finally, since this was an exploratory secondary analysis of a trial which was powered for a clinical feasibility outcome and as such the possibility of Type II error cannot be excluded.

## Conclusions

The question of whether exogenous intravenous fluid has a detrimental effect on EG shedding and inflammation in the setting of sepsis remains unclear. This study did not demonstrate a consistent signal across a range of biomarkers of EG shedding or of endothelial activation or inflammation. Patient heterogeneity, low illness severity and insufficient separation between the groups in the overall fluid volume mean that an effect cannot be excluded. While the results of clinical trials are awaited, a judicious approach to fluid management in sepsis is still recommended [24].

## Supplementary Information

**Additional file 1.** Analysis of biomarkers in those treated per protocol.

**Additional file 2.** Analysis of biomarkers from refresh RCT.

## Data Availability

The data sets used during the current study are available from the corresponding author on reasonable request.

## Abbreviations

ANP: Atrial natriuretic peptide
ED: Emergency Department
EG: Endothelial glycocalyx
ICAM: Intracellular adhesion molecule
ICU: Intensive care unit
IL-6: Interleukin-6
IL-10: Interleukin-10
IV: Intravenous
MLE: Maximum likelihood estimation
NGAL: Neutrophil Gelatinase-Associated Lipocalin
REFRESH: Restricted Fluid Resuscitation in Sepsis-associated Hypotension
RPM: Revolutions per minute
SBP: Systolic blood pressure
SYN-1: Syndecan-1
SYN-4: Syndecan-4
VCAM: Vascular cell adhesion molecule
VEGFR-1: Vascular endothelial growth factor receptor-1
