# Supplementary material for: Effect of intravenous fluid volume on biomarkers of endothelial glycocalyx shedding and inflammation during initial resuscitation of sepsis
Source: Intensive Care Med Exp. 2023 Apr 17;11:21. doi: 10.1186/s40635-023-00508-4 (PMC10106534; doi:10.1186/s40635-023-00508-4)
Supplement: Supplementary file 1 — Additional file 1. Analysis of biomarkers in those treated per protocol. [file 40635_2023_508_MOESM1_ESM.docx]

# Analysis of Biomarkers in those treated per protocol

There were 6 cases in the standard group who didn't receive the minimum fluid volume

ID 17, 25, 72, 81, 86 , 95

There was one case in the restricted group who received fluid in excess of that permitted

ID 68.

. tab restricted per_protocol if rec_num==1

Randomised | per_protocol

Group | 0 1 | Total

-----------+----------------------+----------

Standard | 6 43 | 49

Restricted | 1 49 | 50

-----------+----------------------+----------

Total | 7 92 | 99

After removing the patients who were not treated as per protocol, there are n=43 in the standard group and n=49 in the restricted group.

When the propensity score is applied this is reduced to 39 and 45 respectively (below) which is one less than the full analysis.

. tab restricted per_protocol if rec_num==1 &pscore~=.

| per_protoc

Randomised | ol

Group | 1 | Total

-----------+-----------+----------

Standard | 39 | 39

Restricted | 45 | 45

-----------+-----------+----------

Total | 84 | 84

# Summary of Results

| Biomarker | P value for interaction term | Bootstrapped | Pscore effect^*^ |
| --- | --- | --- | --- |
| Ln_ProANP | 0.2 |  | Significantly associated.  Does not impact on intervention effects |
| Ln_ICAM | 0.27 |  | Not significantly associated. |
| Ln_VCAM | 0.24 |  | Not significantly associated. |
| Ln_Eselectin | 0.97 |  | Significantly associated.  Does not impact on intervention effects |
| Ln_NGAL | 0.18 | 0.29 | Significantly associated.  Does not impact on intervention effects |
| Ln_Resistin | 0.52 | 0.8 | Significantly associated.  Does not impact on intervention effects |
| Ln_Syn1 | 0.87 | 0.91 | Not significantly associated. |
| **Ln_Hyaluronan** | **0.04** | **0.054** | Not significantly associated. |
| **Ln_Il6** | **0.09** | **0.03** | Not significantly associated (but very close) |
| Ln_Il10 | 0.33 | 0.2 | Not significantly associated. |
| Ln_VEGFR1 | 0.95 | 0.93 | Significantly associated.  Does not impact on intervention effects |
| Ln_HepSulfate | 0.85 |  | Significantly associated  Does not impact on intervention effects |
| **Ln_Syn4** | **0.04** | **0.02** | Not significantly associated. |

^*^Significantly associated (or not) is with respect to the outcome ie biomarker

# ProANP

. bysort restricted:tabstat proANPTn ln_proANP if per_protocol==1, ///

> s(n mean sd median min max p25 p75) by(time) col(stats) long f(%8.5g)

-> restricted = Standard

time Variable | N Mean SD p50 Min Max p25 p75

-----------------+--------------------------------------------------------------------------

0 proANPTn | 39 35535 28311 26050 2068 142700 15950 49960

ln_proANP | 39 10.192 .80854 10.168 7.6343 11.868 9.6772 10.819

-----------------+--------------------------------------------------------------------------

3 proANPTn | 39 32928 23338 25570 4488 108700 14430 47900

ln_proANP | 39 10.147 .75633 10.149 8.4092 11.596 9.5771 10.777

-----------------+--------------------------------------------------------------------------

6 proANPTn | 35 26065 19285 20170 5084 74720 12640 30550

ln_proANP | 35 9.9273 .70654 9.912 8.5339 11.222 9.4446 10.327

-----------------+--------------------------------------------------------------------------

24 proANPTn | 35 36066 21863 32660 4800 84430 19190 57630

ln_proANP | 35 10.256 .76959 10.394 8.4764 11.344 9.8621 10.962

-----------------+--------------------------------------------------------------------------

Total proANPTn | 148 32734 23662 24555 2068 142700 14225 47150

ln_proANP | 148 10.133 .76421 10.109 7.6343 11.868 9.5627 10.761

--------------------------------------------------------------------------------------------

-> restricted = Restricted

time Variable | N Mean SD p50 Min Max p25 p75

-----------------+--------------------------------------------------------------------------

0 proANPTn | 45 40982 29064 33090 5992 135250 20200 57590

ln_proANP | 45 10.393 .69478 10.407 8.6982 11.815 9.9134 10.961

-----------------+--------------------------------------------------------------------------

3 proANPTn | 42 34346 30872 21850 2731 137900 14000 45690

ln_proANP | 42 10.108 .83519 9.9914 7.9124 11.834 9.5468 10.73

-----------------+--------------------------------------------------------------------------

6 proANPTn | 41 26960 21392 22440 4397 86370 11410 34530

ln_proANP | 41 9.8884 .82439 10.019 8.3887 11.366 9.3422 10.45

-----------------+--------------------------------------------------------------------------

24 proANPTn | 44 31834 18311 29105 2616 71400 15755 46955

ln_proANP | 44 10.172 .69005 10.278 7.8694 11.176 9.6649 10.757

-----------------+--------------------------------------------------------------------------

Total proANPTn | 172 33679 25750 26405 2616 137900 14805 46535

ln_proANP | 172 10.147 .77629 10.181 7.8694 11.834 9.6027 10.748

--------------------------------------------------------------------------------------------

. mixed ln_proANP i.time##restricted if per_protocol==1 ||RecordID:, mle

Mixed-effects ML regression Number of obs = 320

Group variable: RecordID Number of groups = 89

Obs per group:

min = 1

avg = 3.6

max = 4

Wald chi2(7) = 43.67

Log likelihood = -278.38612 Prob > chi2 = 0.0000

---------------------------------------------------------------------------------

ln_proANP | Coefficient Std. err. z P>|z| [95% conf. interval]

----------------+----------------------------------------------------------------

time |

3 | -.0682925 .0984385 -0.69 0.488 -.2612283 .1246433

6 | -.3049942 .102234 -2.98 0.003 -.5053692 -.1046192

24 | .0313947 .1028871 0.31 0.760 -.1702603 .2330497

|

restricted |

Restricted | .1946257 .1614889 1.21 0.228 -.1218867 .511138

|

time#restricted |

3#Restricted | -.2274488 .1363239 -1.67 0.095 -.4946389 .0397412

6#Restricted | -.2177194 .1393109 -1.56 0.118 -.4907638 .0553251

24#Restricted | -.2664864 .1385794 -1.92 0.054 -.5380971 .0051243

|

_cons | 10.21613 .1183902 86.29 0.000 9.984088 10.44817

---------------------------------------------------------------------------------

------------------------------------------------------------------------------

Random-effects parameters | Estimate Std. err. [95% conf. interval]

-----------------------------+------------------------------------------------

RecordID: Identity |

var(_cons) | .3732186 .064199 .2664062 .5228564

-----------------------------+------------------------------------------------

var(Residual) | .1872899 .0173964 .1561171 .2246871

------------------------------------------------------------------------------

LR test vs. linear model: chibar2(01) = 169.34 Prob >= chibar2 = 0.0000

. testparm time#restricted

( 1) [ln_proANP]3.time#1.restricted = 0

( 2) [ln_proANP]6.time#1.restricted = 0

( 3) [ln_proANP]24.time#1.restricted = 0

chi2( 3) = 4.65

Prob > chi2 = 0.1989

. mixed ln_proANP i.time##restricted if pscore~=. & per_protocol==1 ||RecordID:, mle

Mixed-effects ML regression Number of obs = 304

Group variable: RecordID Number of groups = 84

Obs per group:

min = 1

avg = 3.6

max = 4

Wald chi2(7) = 43.13

Log likelihood = -265.95105 Prob > chi2 = 0.0000

---------------------------------------------------------------------------------

ln_proANP | Coefficient Std. err. z P>|z| [95% conf. interval]

----------------+----------------------------------------------------------------

time |

3 | -.072539 .1003327 -0.72 0.470 -.2691875 .1241094

6 | -.3067024 .1043127 -2.94 0.003 -.5111515 -.1022532

24 | .0196344 .1052987 0.19 0.852 -.1867472 .226016

|

restricted |

Restricted | .2374997 .166413 1.43 0.154 -.0886639 .5636632

|

time#restricted |

3#Restricted | -.2587226 .1405638 -1.84 0.066 -.5342227 .0167775

6#Restricted | -.2342127 .143101 -1.64 0.102 -.5146855 .0462601

24#Restricted | -.3154861 .1430256 -2.21 0.027 -.5958112 -.035161

|

_cons | 10.22796 .1211346 84.43 0.000 9.990538 10.46538

---------------------------------------------------------------------------------

------------------------------------------------------------------------------

Random-effects parameters | Estimate Std. err. [95% conf. interval]

-----------------------------+------------------------------------------------

RecordID: Identity |

var(_cons) | .3763185 .0666938 .2658885 .5326127

-----------------------------+------------------------------------------------

var(Residual) | .1895347 .0180574 .1572508 .2284466

------------------------------------------------------------------------------

LR test vs. linear model: chibar2(01) = 159.39 Prob >= chibar2 = 0.0000

. testparm time#restricted

( 1) [ln_proANP]3.time#1.restricted = 0

( 2) [ln_proANP]6.time#1.restricted = 0

( 3) [ln_proANP]24.time#1.restricted = 0

chi2( 3) = 5.82

Prob > chi2 = 0.1206

. mixed ln_proANP i.time##restricted pscore if per_protocol==1 ||RecordID:, mle

Mixed-effects ML regression Number of obs = 304

Group variable: RecordID Number of groups = 84

Obs per group:

min = 1

avg = 3.6

max = 4

Wald chi2(8) = 60.10

Log likelihood = -258.15534 Prob > chi2 = 0.0000

---------------------------------------------------------------------------------

ln_proANP | Coefficient Std. err. z P>|z| [95% conf. interval]

----------------+----------------------------------------------------------------

time |

3 | -.0683354 .1004037 -0.68 0.496 -.2651231 .1284524

6 | -.2982992 .1043471 -2.86 0.004 -.5028157 -.0937827

24 | .0205771 .1053196 0.20 0.845 -.1858456 .2269997

|

restricted |

Restricted | .1313607 .1573694 0.83 0.404 -.1770777 .4397991

|

time#restricted |

3#Restricted | -.2587801 .140618 -1.84 0.066 -.5343863 .016826

6#Restricted | -.2438425 .1431552 -1.70 0.089 -.5244216 .0367365

24#Restricted | -.3154534 .1430525 -2.21 0.027 -.5958311 -.0350757

|

pscore | 2.851197 .6870841 4.15 0.000 1.504537 4.197857

_cons | 8.756566 .3719584 23.54 0.000 8.027541 9.485591

---------------------------------------------------------------------------------

------------------------------------------------------------------------------

Random-effects parameters | Estimate Std. err. [95% conf. interval]

-----------------------------+------------------------------------------------

RecordID: Identity |

var(_cons) | .3014502 .0554539 .2101992 .4323149

-----------------------------+------------------------------------------------

var(Residual) | .1898651 .0181176 .1574781 .2289127

------------------------------------------------------------------------------

LR test vs. linear model: chibar2(01) = 127.93 Prob >= chibar2 = 0.0000

. testparm time#restricted

( 1) [ln_proANP]3.time#1.restricted = 0

( 2) [ln_proANP]6.time#1.restricted = 0

( 3) [ln_proANP]24.time#1.restricted = 0

chi2( 3) = 5.89

Prob > chi2 = 0.1172

. mixed ln_proANP i.time i.restricted if per_protocol==1 ||RecordID:, mle

Mixed-effects ML regression Number of obs = 320

Group variable: RecordID Number of groups = 89

Obs per group:

min = 1

avg = 3.6

max = 4

Wald chi2(4) = 38.26

Log likelihood = -280.6917 Prob > chi2 = 0.0000

------------------------------------------------------------------------------

ln_proANP | Coefficient Std. err. z P>|z| [95% conf. interval]

-------------+----------------------------------------------------------------

time |

3 | -.1881872 .0687512 -2.74 0.006 -.3229372 -.0534373

6 | -.422391 .0701128 -6.02 0.000 -.5598096 -.2849725

24 | -.1142548 .0695766 -1.64 0.101 -.2506224 .0221128

|

restricted |

Restricted | .0204155 .1394053 0.15 0.884 -.2528138 .2936448

_cons | 10.30928 .1102463 93.51 0.000 10.0932 10.52536

------------------------------------------------------------------------------

------------------------------------------------------------------------------

Random-effects parameters | Estimate Std. err. [95% conf. interval]

-----------------------------+------------------------------------------------

RecordID: Identity |

var(_cons) | .372809 .064294 .2658823 .522737

-----------------------------+------------------------------------------------

var(Residual) | .1909495 .0177353 .1591693 .2290749

------------------------------------------------------------------------------

LR test vs. linear model: chibar2(01) = 166.53 Prob >= chibar2 = 0.0000

. mixed ln_proANP i.time i.restricted pscore if per_protocol==1 ||RecordID:, mle

Mixed-effects ML regression Number of obs = 304

Group variable: RecordID Number of groups = 84

Obs per group:

min = 1

avg = 3.6

max = 4

Wald chi2(5) = 53.22

Log likelihood = -261.0626 Prob > chi2 = 0.0000

------------------------------------------------------------------------------

ln_proANP | Coefficient Std. err. z P>|z| [95% conf. interval]

-------------+----------------------------------------------------------------

time |

3 | -.2017937 .0711962 -2.83 0.005 -.3413357 -.0622517

6 | -.4273677 .0723402 -5.91 0.000 -.5691519 -.2855834

24 | -.1488483 .0721721 -2.06 0.039 -.2903031 -.0073935

|

restricted |

Restricted | -.0677049 .1333622 -0.51 0.612 -.3290901 .1936802

pscore | 2.846641 .6875657 4.14 0.000 1.499037 4.194245

_cons | 8.862791 .3694396 23.99 0.000 8.138703 9.58688

------------------------------------------------------------------------------

------------------------------------------------------------------------------

Random-effects parameters | Estimate Std. err. [95% conf. interval]

-----------------------------+------------------------------------------------

RecordID: Identity |

var(_cons) | .3006746 .0555523 .2093291 .4318808

-----------------------------+------------------------------------------------

var(Residual) | .1948108 .0185879 .161583 .2348717

------------------------------------------------------------------------------

LR test vs. linear model: chibar2(01) = 124.77 Prob >= chibar2 = 0.0000

# ICAM

. bysort restricted:tabstat ICAMTn ln_ICAM if per_protocol==1, ///

> s(n mean sd median min max p25 p75) by(time) col(stats) long f(%7.5g)

-> restricted = Standard

time Variable | N Mean SD p50 Min Max p25 p75

------------------+--------------------------------------------------------------------------

0 ICAMTn | 39 1045.3 964.27 669.33 99.955 4152.3 467.29 1205.1

ln_ICAM | 39 6.5688 .91895 6.5063 4.6047 8.3314 6.1469 7.0943

------------------+--------------------------------------------------------------------------

3 ICAMTn | 39 842.65 699.67 544.33 87.05 2853.1 398.3 1026.2

ln_ICAM | 39 6.4399 .79479 6.2995 4.4665 7.9561 5.9872 6.9336

------------------+--------------------------------------------------------------------------

6 ICAMTn | 35 1269.5 1925.4 777.11 183.54 10538 395.58 1189.5

ln_ICAM | 35 6.655 .89395 6.6556 5.2124 9.2627 5.9804 7.0813

------------------+--------------------------------------------------------------------------

24 ICAMTn | 35 1041.9 1136.4 505.6 111.95 4093.9 318.79 1350

ln_ICAM | 35 6.4648 .99191 6.2257 4.7181 8.3172 5.7645 7.2079

------------------+--------------------------------------------------------------------------

Total ICAMTn | 148 1044.1 1243.3 644.85 87.05 10538 404.83 1190

ln_ICAM | 148 6.5306 .89514 6.4689 4.4665 9.2627 6.0033 7.0817

---------------------------------------------------------------------------------------------

-> restricted = Restricted

time Variable | N Mean SD p50 Min Max p25 p75

-----------------+--------------------------------------------------------------------------

0 ICAMTn | 45 1125.1 886.64 846.58 102.74 4252.1 615.74 1471.5

ln_ICAM | 45 6.764 .74788 6.7412 4.6322 8.3552 6.4228 7.2941

-----------------+--------------------------------------------------------------------------

3 ICAMTn | 43 991.67 955.47 632.34 182.93 4518.4 411.01 1032.7

ln_ICAM | 43 6.5783 .77744 6.4494 5.2091 8.4159 6.0186 6.9399

-----------------+--------------------------------------------------------------------------

6 ICAMTn | 41 1396.5 1398.4 1121.9 286.64 8571.6 637.23 1617.5

ln_ICAM | 41 6.9715 .69192 7.0228 5.6582 9.0562 6.4571 7.3886

-----------------+--------------------------------------------------------------------------

24 ICAMTn | 44 1073.9 1427.2 672.7 93.58 6490.4 325.26 951.16

ln_ICAM | 44 6.4503 .98521 6.5109 4.5388 8.7781 5.7846 6.857

-----------------+--------------------------------------------------------------------------

Total ICAMTn | 173 1143.2 1187.6 770.69 93.58 8571.6 484.92 1316.9

ln_ICAM | 173 6.6872 .82631 6.6473 4.5388 9.0562 6.184 7.1831

--------------------------------------------------------------------------------------------

. mixed ln_ICAM i.time##restricted if per_protocol==1 ||RecordID:, mle

Mixed-effects ML regression Number of obs = 321

Group variable: RecordID Number of groups = 89

Obs per group:

min = 1

avg = 3.6

max = 4

Wald chi2(7) = 26.00

Log likelihood = -350.7908 Prob > chi2 = 0.0005

---------------------------------------------------------------------------------

ln_ICAM | Coefficient Std. err. z P>|z| [95% conf. interval]

----------------+----------------------------------------------------------------

time |

3 | -.1401584 .1318919 -1.06 0.288 -.3986618 .1183451

6 | .0984173 .1367943 0.72 0.472 -.1696945 .3665292

24 | -.0903023 .1374987 -0.66 0.511 -.3597948 .1791901

|

restricted |

Restricted | .2039395 .1816703 1.12 0.262 -.1521278 .5600068

|

time#restricted |

3#Restricted | -.038268 .181804 -0.21 0.833 -.3945973 .3180612

6#Restricted | .1366583 .1863756 0.73 0.463 -.2286311 .5019477

24#Restricted | -.2338952 .1853024 -1.26 0.207 -.5970813 .1292909

|

_cons | 6.553254 .1331046 49.23 0.000 6.292373 6.814134

---------------------------------------------------------------------------------

------------------------------------------------------------------------------

Random-effects parameters | Estimate Std. err. [95% conf. interval]

-----------------------------+------------------------------------------------

RecordID: Identity |

var(_cons) | .3658515 .0694089 .2522412 .5306323

-----------------------------+------------------------------------------------

var(Residual) | .3366027 .0311306 .2807983 .4034974

------------------------------------------------------------------------------

LR test vs. linear model: chibar2(01) = 99.00 Prob >= chibar2 = 0.0000

. testparm time#restricted

( 1) [ln_ICAM]3.time#1.restricted = 0

( 2) [ln_ICAM]6.time#1.restricted = 0

( 3) [ln_ICAM]24.time#1.restricted = 0

chi2( 3) = 3.88

Prob > chi2 = 0.2742

. mixed ln_ICAM i.time##restricted pscore if per_protocol==1 ||RecordID:, mle

Mixed-effects ML regression Number of obs = 305

Group variable: RecordID Number of groups = 84

Obs per group:

min = 1

avg = 3.6

max = 4

Wald chi2(8) = 27.33

Log likelihood = -336.58634 Prob > chi2 = 0.0006

---------------------------------------------------------------------------------

ln_ICAM | Coefficient Std. err. z P>|z| [95% conf. interval]

----------------+----------------------------------------------------------------

time |

3 | -.1251919 .1364281 -0.92 0.359 -.3925859 .1422022

6 | .1162945 .141628 0.82 0.412 -.1612912 .3938802

24 | -.0791706 .1429321 -0.55 0.580 -.3593124 .2009712

|

restricted |

Restricted | .1584643 .1880487 0.84 0.399 -.2101044 .5270329

|

time#restricted |

3#Restricted | -.0272574 .1901203 -0.14 0.886 -.3998863 .3453714

6#Restricted | .1189678 .194276 0.61 0.540 -.2618062 .4997418

24#Restricted | -.2503294 .1941451 -1.29 0.197 -.6308467 .1301879

|

pscore | 1.478129 .7714786 1.92 0.055 -.033941 2.9902

_cons | 5.771267 .4202726 13.73 0.000 4.947547 6.594986

---------------------------------------------------------------------------------

------------------------------------------------------------------------------

Random-effects parameters | Estimate Std. err. [95% conf. interval]

-----------------------------+------------------------------------------------

RecordID: Identity |

var(_cons) | .3487119 .0694156 .2360609 .5151211

-----------------------------+------------------------------------------------

var(Residual) | .3508892 .0332762 .2913716 .4225642

------------------------------------------------------------------------------

LR test vs. linear model: chibar2(01) = 85.70 Prob >= chibar2 = 0.0000

. testparm time#restricted

( 1) [ln_ICAM]3.time#1.restricted = 0

( 2) [ln_ICAM]6.time#1.restricted = 0

( 3) [ln_ICAM]24.time#1.restricted = 0

chi2( 3) = 3.60

Prob > chi2 = 0.3078

. mixed ln_ICAM i.time i.restricted if per_protocol==1 ||RecordID:, mle

Mixed-effects ML regression Number of obs = 321

Group variable: RecordID Number of groups = 89

Obs per group:

min = 1

avg = 3.6

max = 4

Wald chi2(4) = 21.75

Log likelihood = -352.71479 Prob > chi2 = 0.0002

------------------------------------------------------------------------------

ln_ICAM | Coefficient Std. err. z P>|z| [95% conf. interval]

-------------+----------------------------------------------------------------

time |

3 | -.1603982 .0915695 -1.75 0.080 -.3398711 .0190747

6 | .1708723 .0937178 1.82 0.068 -.0128113 .3545559

24 | -.219913 .0929643 -2.37 0.018 -.4021197 -.0377063

|

restricted |

Restricted | .1672912 .1447515 1.16 0.248 -.1164166 .4509989

_cons | 6.573132 .1195956 54.96 0.000 6.338729 6.807535

------------------------------------------------------------------------------

------------------------------------------------------------------------------

Random-effects parameters | Estimate Std. err. [95% conf. interval]

-----------------------------+------------------------------------------------

RecordID: Identity |

var(_cons) | .3628357 .069168 .2497146 .5272009

-----------------------------+------------------------------------------------

var(Residual) | .3425894 .0316742 .2858089 .4106502

------------------------------------------------------------------------------

LR test vs. linear model: chibar2(01) = 96.69 Prob >= chibar2 = 0.0000

. mixed ln_ICAM i.time i.restricted pscore if per_protocol==1 ||RecordID:, mle

Mixed-effects ML regression Number of obs = 305

Group variable: RecordID Number of groups = 84

Obs per group:

min = 1

avg = 3.6

max = 4

Wald chi2(5) = 23.43

Log likelihood = -338.37054 Prob > chi2 = 0.0003

------------------------------------------------------------------------------

ln_ICAM | Coefficient Std. err. z P>|z| [95% conf. interval]

-------------+----------------------------------------------------------------

time |

3 | -.1394399 .095834 -1.46 0.146 -.3272711 .0483914

6 | .1789265 .0977541 1.83 0.067 -.012668 .3705211

24 | -.2161765 .097532 -2.22 0.027 -.4073358 -.0250173

|

restricted |

Restricted | .1179752 .1494077 0.79 0.430 -.1748584 .4108088

pscore | 1.501564 .770188 1.95 0.051 -.0079767 3.011105

_cons | 5.779523 .4150092 13.93 0.000 4.96612 6.592926

------------------------------------------------------------------------------

------------------------------------------------------------------------------

Random-effects parameters | Estimate Std. err. [95% conf. interval]

-----------------------------+------------------------------------------------

RecordID: Identity |

var(_cons) | .3457326 .0691984 .2335459 .5118096

-----------------------------+------------------------------------------------

var(Residual) | .3569709 .0338457 .2964336 .4298711

------------------------------------------------------------------------------

LR test vs. linear model: chibar2(01) = 83.60 Prob >= chibar2 = 0.0000

# VCAM

. bysort restricted: tabstat VCAMTn ln_VCAM if per_protocol==1, ///

> s(n mean sd median min max p25 p75) by(time) col(stats) long f(%7.5g)

-> restricted = Standard

time Variable | N Mean SD p50 Min Max p25 p75

-----------------+--------------------------------------------------------------------------

0 VCAMTn | 39 3354.3 2556.8 2987.9 153.73 11527 1180.6 4872.6

ln_VCAM | 39 7.8024 .88654 8.0023 5.0352 9.3525 7.0737 8.4914

-----------------+--------------------------------------------------------------------------

3 VCAMTn | 39 2443.7 2136.2 1655.2 499.92 9957.1 917.59 3786.8

ln_VCAM | 39 7.4628 .83195 7.4117 6.2144 9.206 6.8218 8.2393

-----------------+--------------------------------------------------------------------------

6 VCAMTn | 35 3816.3 4007.3 2026.6 512.36 16202 1560.5 4765

ln_VCAM | 35 7.8524 .8592 7.6141 6.239 9.6929 7.3528 8.4691

-----------------+--------------------------------------------------------------------------

24 VCAMTn | 35 2575.6 3284.9 1252.5 161.31 13574 731.86 2342

ln_VCAM | 35 7.3023 1.0196 7.1329 5.0833 9.5159 6.5956 7.7588

-----------------+--------------------------------------------------------------------------

Total VCAMTn | 148 3039.4 3065.1 1856.1 153.73 16202 1013.8 3884.9

ln_VCAM | 148 7.6065 .91945 7.5262 5.0352 9.6929 6.9215 8.2649

--------------------------------------------------------------------------------------------

-> restricted = Restricted

time Variable | N Mean SD p50 Min Max p25 p75

-----------------+-------------------------------------------------------------------------

0 VCAMTn | 45 3688.7 2709.9 3043.2 98.276 12040 1858.8 4292

ln_VCAM | 45 7.9017 .927 8.0207 4.5878 9.396 7.5277 8.3645

-----------------+-------------------------------------------------------------------------

3 VCAMTn | 43 2769.7 2175.6 2069.4 419.89 8683.3 1041.1 4537.7

ln_VCAM | 43 7.5942 .86689 7.635 6.04 9.0692 6.948 8.4202

-----------------+-------------------------------------------------------------------------

6 VCAMTn | 41 4626 3390.3 3590.6 774.68 13345 2345.6 5830.5

ln_VCAM | 41 8.1739 .76256 8.1861 6.6525 9.4989 7.7603 8.6709

-----------------+-------------------------------------------------------------------------

24 VCAMTn | 44 2669 4184.5 1017.1 196.76 20071 561.39 2669.6

ln_VCAM | 44 7.1804 1.1397 6.9123 5.282 9.907 6.3303 7.8895

-----------------+-------------------------------------------------------------------------

Total VCAMTn |173 3423.1 3271.2 2489.6 98.276 20071 1136.1 4292

ln_VCAM |173 7.7063 .99965 7.8199 4.5878 9.907 7.0354 8.3645

-------------------------------------------------------------------------------------------

. mixed ln_VCAM i.time##restricted if per_protocol==1 ||RecordID:, mle

Mixed-effects ML regression Number of obs = 321

Group variable: RecordID Number of groups = 89

Obs per group:

min = 1

avg = 3.6

max = 4

Wald chi2(7) = 57.47

Log likelihood = -403.28407 Prob > chi2 = 0.0000

---------------------------------------------------------------------------------

ln_VCAM | Coefficient Std. err. z P>|z| [95% conf. interval]

----------------+----------------------------------------------------------------

time |

3 | -.3556033 .166098 -2.14 0.032 -.6811494 -.0300573

6 | .0376181 .1719426 0.22 0.827 -.2993832 .3746194

24 | -.4850139 .1725803 -2.81 0.005 -.8232651 -.1467627

|

restricted |

Restricted | .107339 .1984404 0.54 0.589 -.281597 .4962751

|

time#restricted |

3#Restricted | .0596081 .2287651 0.26 0.794 -.3887632 .5079793

6#Restricted | .2379017 .234316 1.02 0.310 -.2213492 .6971526

24#Restricted | -.2462229 .2327619 -1.06 0.290 -.7024279 .2099821

|

_cons | 7.789649 .1453122 53.61 0.000 7.504842 8.074455

---------------------------------------------------------------------------------

------------------------------------------------------------------------------

Random-effects parameters | Estimate Std. err. [95% conf. interval]

-----------------------------+------------------------------------------------

RecordID: Identity |

var(_cons) | .2958864 .0695886 .1866095 .469155

-----------------------------+------------------------------------------------

var(Residual) | .5347196 .0497709 .4455511 .6417335

------------------------------------------------------------------------------

LR test vs. linear model: chibar2(01) = 42.98 Prob >= chibar2 = 0.0000

. testparm time#restricted

( 1) [ln_VCAM]3.time#1.restricted = 0

( 2) [ln_VCAM]6.time#1.restricted = 0

( 3) [ln_VCAM]24.time#1.restricted = 0

chi2( 3) = 4.21

Prob > chi2 = 0.2399

. mixed ln_VCAM i.time##restricted pscore if per_protocol==1 ||RecordID:, mle

Mixed-effects ML regression Number of obs = 305

Group variable: RecordID Number of groups = 84

Obs per group:

min = 1

avg = 3.6

max = 4

Wald chi2(8) = 51.07

Log likelihood = -387.11074 Prob > chi2 = 0.0000

---------------------------------------------------------------------------------

ln_VCAM | Coefficient Std. err. z P>|z| [95% conf. interval]

----------------+----------------------------------------------------------------

time |

3 | -.3437995 .1705966 -2.02 0.044 -.6781626 -.0094364

6 | .0527965 .1767983 0.30 0.765 -.2937219 .3993148

24 | -.4684027 .1783891 -2.63 0.009 -.818039 -.1187664

|

restricted |

Restricted | .0454571 .2077403 0.22 0.827 -.3617065 .4526206

|

time#restricted |

3#Restricted | .0846065 .2375366 0.36 0.722 -.3809566 .5501697

6#Restricted | .2169044 .2425926 0.89 0.371 -.2585683 .6923771

24#Restricted | -.2385279 .2423433 -0.98 0.325 -.7135122 .2364563

|

pscore | .9493211 .7789505 1.22 0.223 -.577394 2.476036

_cons | 7.308112 .4287803 17.04 0.000 6.467718 8.148506

---------------------------------------------------------------------------------

------------------------------------------------------------------------------

Random-effects parameters | Estimate Std. err. [95% conf. interval]

-----------------------------+------------------------------------------------

RecordID: Identity |

var(_cons) | .3022097 .0731575 .1880415 .4856946

-----------------------------+------------------------------------------------

var(Residual) | .5494703 .0524433 .4557248 .6624998

------------------------------------------------------------------------------

LR test vs. linear model: chibar2(01) = 40.44 Prob >= chibar2 = 0.0000

. testparm time#restricted

( 1) [ln_VCAM]3.time#1.restricted = 0

( 2) [ln_VCAM]6.time#1.restricted = 0

( 3) [ln_VCAM]24.time#1.restricted = 0

chi2( 3) = 3.57

Prob > chi2 = 0.3114

. mixed ln_VCAM i.time i.restricted if per_protocol==1 ||RecordID:, mle

Mixed-effects ML regression Number of obs = 321

Group variable: RecordID Number of groups = 89

Obs per group:

min = 1

avg = 3.6

max = 4

Wald chi2(4) = 52.24

Log likelihood = -405.36254 Prob > chi2 = 0.0000

------------------------------------------------------------------------------

ln_VCAM | Coefficient Std. err. z P>|z| [95% conf. interval]

-------------+----------------------------------------------------------------

time |

3 | -.3242691 .1153673 -2.81 0.005 -.5503848 -.0981534

6 | .1646939 .1179871 1.40 0.163 -.0665565 .3959443

24 | -.6225176 .116951 -5.32 0.000 -.8517373 -.3932978

|

restricted |

Restricted | .1165752 .1422703 0.82 0.413 -.1622694 .3954199

_cons | 7.785081 .125271 62.15 0.000 7.539554 8.030607

------------------------------------------------------------------------------

------------------------------------------------------------------------------

Random-effects parameters | Estimate Std. err. [95% conf. interval]

-----------------------------+------------------------------------------------

RecordID: Identity |

var(_cons) | .2897622 .0689715 .1817322 .4620101

-----------------------------+------------------------------------------------

var(Residual) | .545754 .0507457 .4548308 .6548533

------------------------------------------------------------------------------

LR test vs. linear model: chibar2(01) = 41.12 Prob >= chibar2 = 0.0000

. mixed ln_VCAM i.time i.restricted pscore if per_protocol==1 ||RecordID:, mle

Mixed-effects ML regression Number of obs = 305

Group variable: RecordID Number of groups = 84

Obs per group:

min = 1

avg = 3.6

max = 4

Wald chi2(5) = 46.71

Log likelihood = -388.87858 Prob > chi2 = 0.0000

------------------------------------------------------------------------------

ln_VCAM | Coefficient Std. err. z P>|z| [95% conf. interval]

-------------+----------------------------------------------------------------

time |

3 | -.3003097 .1197872 -2.51 0.012 -.5350884 -.065531

6 | .1674851 .1221192 1.37 0.170 -.0718641 .4068343

24 | -.600532 .1218024 -4.93 0.000 -.8392602 -.3618037

|

restricted |

Restricted | .0595074 .1507304 0.39 0.693 -.2359187 .3549335

pscore | .9785329 .7763603 1.26 0.208 -.5431053 2.500171

_cons | 7.284817 .4204094 17.33 0.000 6.46083 8.108805

------------------------------------------------------------------------------

------------------------------------------------------------------------------

Random-effects parameters | Estimate Std. err. [95% conf. interval]

-----------------------------+------------------------------------------------

RecordID: Identity |

var(_cons) | .2967282 .0726516 .1836321 .4794782

-----------------------------+------------------------------------------------

var(Residual) | .5595417 .0533696 .4641347 .6745605

------------------------------------------------------------------------------

LR test vs. linear model: chibar2(01) = 38.84 Prob >= chibar2 = 0.0000

# Eselectin

. bysort restricted: tabstat EselectinTn ln_Eselectin if per_protocol==1, ///

> s(n mean sd median min max p25 p75) by(time) col(stats) long f(%8.6g)

-> restricted = Standard

time Variable | N Mean SD p50 Min Max p25 p75

-------------------+--------------------------------------------------------------------------

0 EselectinTn | 39 51326.7 49272.9 29776.3 4276.98 173432 10789.9 101241

ln_Eselectin | 39 10.331 1.08653 10.3015 8.361 12.0635 9.28636 11.5253

-------------------+--------------------------------------------------------------------------

3 EselectinTn | 38 44232.1 40415.3 27704.8 3079.69 152063 13399.9 59561

ln_Eselectin | 38 10.2425 1.03549 10.2281 8.03258 11.9321 9.503 10.9948

-------------------+--------------------------------------------------------------------------

6 EselectinTn | 35 45323.9 42035.4 25041.9 6761.25 151416 11109.8 74509.1

ln_Eselectin | 35 10.2786 .976928 10.1283 8.81896 11.9278 9.31558 11.2187

-------------------+--------------------------------------------------------------------------

24 EselectinTn | 35 36469.8 28264 28861.1 5602.95 119418 14318.7 59918.6

ln_Eselectin | 35 10.1974 .823919 10.2703 8.63105 11.6904 9.56932 11.0007

-------------------+--------------------------------------------------------------------------

Total EselectinTn | 147 44526.2 40883.4 28234.7 3079.69 173432 12375.8 66843.9

ln_Eselectin | 147 10.2638 .980701 10.2483 8.03258 12.0635 9.4235 11.1101

----------------------------------------------------------------------------------------------

-> restricted = Restricted

time Variable | N Mean SD p50 Min Max p25 p75

-------------------+-------------------------------------------------------------------------

0 EselectinTn | 46 60889.1 72141 30434.6 3347.52 317128 13527.9 93542.6

ln_Eselectin | 46 10.4057 1.14721 10.3215 8.11598 12.6671 9.51251 11.4462

-------------------+-------------------------------------------------------------------------

3 EselectinTn | 40 54128.8 61086.1 30935.2 4473.5 308372 11630.3 85060.6

ln_Eselectin | 40 10.3463 1.08692 10.3395 8.40593 12.6391 9.36124 11.35

-------------------+-------------------------------------------------------------------------

6 EselectinTn | 41 53945.2 54727.8 24757.9 3966.48 185489 9587.07 96211.9

ln_Eselectin | 41 10.3072 1.15542 10.1169 8.28563 12.1307 9.16817 11.4743

-------------------+-------------------------------------------------------------------------

24 EselectinTn | 44 44161.2 48317.8 21754.3 4660.06 219418 11192.6 63983.8

ln_Eselectin | 44 10.176 1.03167 9.98668 8.44678 12.2987 9.32293 11.0658

-------------------+-------------------------------------------------------------------------

Total EselectinTn |171 53338.6 59719.2 28440.3 3347.52 317128 11900.6 79980.2

ln_Eselectin |171 10.3091 1.10008 10.2556 8.11598 12.6671 9.38434 11.2895

---------------------------------------------------------------------------------------------

. mixed ln_Eselectin i.time##restricted if per_protocol==1||RecordID:, mle

Mixed-effects ML regression Number of obs = 318

Group variable: RecordID Number of groups = 89

Obs per group:

min = 1

avg = 3.6

max = 4

Wald chi2(7) = 13.53

Log likelihood = -284.70319 Prob > chi2 = 0.0601

---------------------------------------------------------------------------------

ln_Eselectin | Coefficient Std. err. z P>|z| [95% conf. interval]

----------------+----------------------------------------------------------------

time |

3 | -.1086419 .0874291 -1.24 0.214 -.2799999 .062716

6 | -.0621164 .0900553 -0.69 0.490 -.2386214 .1143887

24 | -.2131251 .0907738 -2.35 0.019 -.3910385 -.0352118

|

restricted |

Restricted | .1079454 .2246395 0.48 0.631 -.3323399 .5482307

|

time#restricted |

3#Restricted | -.0074635 .1212485 -0.06 0.951 -.2451062 .2301793

6#Restricted | -.0529392 .12236 -0.43 0.665 -.2927604 .1868821

24#Restricted | -.0076715 .1214741 -0.06 0.950 -.2457563 .2304134

|

_cons | 10.30903 .1650608 62.46 0.000 9.985518 10.63254

---------------------------------------------------------------------------------

------------------------------------------------------------------------------

Random-effects parameters | Estimate Std. err. [95% conf. interval]

-----------------------------+------------------------------------------------

RecordID: Identity |

var(_cons) | .9613192 .1509333 .7066812 1.307711

-----------------------------+------------------------------------------------

var(Residual) | .1439723 .0134625 .1198629 .172931

------------------------------------------------------------------------------

LR test vs. linear model: chibar2(01) = 358.52 Prob >= chibar2 = 0.0000

. testparm time#restricted

( 1) [ln_Eselectin]3.time#1.restricted = 0

( 2) [ln_Eselectin]6.time#1.restricted = 0

( 3) [ln_Eselectin]24.time#1.restricted = 0

chi2( 3) = 0.23

Prob > chi2 = 0.9730

. mixed ln_Eselectin i.time##restricted if pscore~=. & per_protocol==1 ||RecordID:, mle

Mixed-effects ML regression Number of obs = 302

Group variable: RecordID Number of groups = 84

Obs per group:

min = 1

avg = 3.6

max = 4

Wald chi2(7) = 11.21

Log likelihood = -268.94949 Prob > chi2 = 0.1297

---------------------------------------------------------------------------------

ln_Eselectin | Coefficient Std. err. z P>|z| [95% conf. interval]

----------------+----------------------------------------------------------------

time |

3 | -.101494 .0892869 -1.14 0.256 -.2764931 .0735052

6 | -.0557855 .092043 -0.61 0.544 -.2361865 .1246155

24 | -.1975308 .0929056 -2.13 0.033 -.3796225 -.0154391

|

restricted |

Restricted | .146768 .2243528 0.65 0.513 -.2929555 .5864914

|

time#restricted |

3#Restricted | -.0073192 .1253298 -0.06 0.953 -.2529611 .2383228

6#Restricted | -.0451352 .1258709 -0.36 0.720 -.2918376 .2015671

24#Restricted | -.0090009 .125418 -0.07 0.943 -.2548156 .2368139

|

_cons | 10.27677 .164033 62.65 0.000 9.955267 10.59826

---------------------------------------------------------------------------------

------------------------------------------------------------------------------

Random-effects parameters | Estimate Std. err. [95% conf. interval]

-----------------------------+------------------------------------------------

RecordID: Identity |

var(_cons) | .8980977 .1457518 .6534052 1.234425

-----------------------------+------------------------------------------------

var(Residual) | .1461964 .014018 .1211488 .1764225

------------------------------------------------------------------------------

LR test vs. linear model: chibar2(01) = 324.78 Prob >= chibar2 = 0.0000

. testparm time#restricted

( 1) [ln_Eselectin]3.time#1.restricted = 0

( 2) [ln_Eselectin]6.time#1.restricted = 0

( 3) [ln_Eselectin]24.time#1.restricted = 0

chi2( 3) = 0.15

Prob > chi2 = 0.9851

. mixed ln_Eselectin i.time##restricted pscore if per_protocol==1 ||RecordID:, mle

Mixed-effects ML regression Number of obs = 302

Group variable: RecordID Number of groups = 84

Obs per group:

min = 1

avg = 3.6

max = 4

Wald chi2(8) = 16.65

Log likelihood = -266.31403 Prob > chi2 = 0.0340

---------------------------------------------------------------------------------

ln_Eselectin | Coefficient Std. err. z P>|z| [95% conf. interval]

----------------+----------------------------------------------------------------

time |

3 | -.100363 .0892805 -1.12 0.261 -.2753495 .0746236

6 | -.0537569 .0920354 -0.58 0.559 -.234143 .1266292

24 | -.197247 .0928948 -2.12 0.034 -.3793175 -.0151765

|

restricted |

Restricted | .0515968 .2219356 0.23 0.816 -.3833891 .4865826

|

time#restricted |

3#Restricted | -.0068728 .1253173 -0.05 0.956 -.2524903 .2387446

6#Restricted | -.0471531 .1258618 -0.37 0.708 -.2938377 .1995315

24#Restricted | -.0087266 .1254079 -0.07 0.945 -.2545215 .2370683

|

pscore | 2.524169 1.082353 2.33 0.020 .4027965 4.645541

_cons | 8.975225 .580453 15.46 0.000 7.837558 10.11289

---------------------------------------------------------------------------------

------------------------------------------------------------------------------

Random-effects parameters | Estimate Std. err. [95% conf. interval]

-----------------------------+------------------------------------------------

RecordID: Identity |

var(_cons) | .8408485 .1368866 .6111471 1.156884

-----------------------------+------------------------------------------------

var(Residual) | .1461956 .0140176 .1211487 .1764208

------------------------------------------------------------------------------

LR test vs. linear model: chibar2(01) = 314.29 Prob >= chibar2 = 0.0000

. testparm time#restricted

( 1) [ln_Eselectin]3.time#1.restricted = 0

( 2) [ln_Eselectin]6.time#1.restricted = 0

( 3) [ln_Eselectin]24.time#1.restricted = 0

chi2( 3) = 0.17

Prob > chi2 = 0.9827

. mixed ln_Eselectin i.time i.restricted if per_protocol==1 ||RecordID:, mle

Mixed-effects ML regression Number of obs = 318

Group variable: RecordID Number of groups = 89

Obs per group:

min = 1

avg = 3.6

max = 4

Wald chi2(4) = 13.29

Log likelihood = -284.8169 Prob > chi2 = 0.0099

------------------------------------------------------------------------------

ln_Eselectin | Coefficient Std. err. z P>|z| [95% conf. interval]

-------------+----------------------------------------------------------------

time |

3 | -.1130601 .0605769 -1.87 0.062 -.2317886 .0056683

6 | -.0906738 .0609892 -1.49 0.137 -.2102105 .0288628

24 | -.2174527 .0603433 -3.60 0.000 -.3357235 -.099182

|

restricted |

Restricted | .0921086 .213208 0.43 0.666 -.3257713 .5099886

_cons | 10.31758 .1605479 64.26 0.000 10.00291 10.63224

------------------------------------------------------------------------------

------------------------------------------------------------------------------

Random-effects parameters | Estimate Std. err. [95% conf. interval]

-----------------------------+------------------------------------------------

RecordID: Identity |

var(_cons) | .9612831 .1509296 .7066517 1.307667

-----------------------------+------------------------------------------------

var(Residual) | .1441149 .0134757 .1199819 .173102

------------------------------------------------------------------------------

LR test vs. linear model: chibar2(01) = 358.45 Prob >= chibar2 = 0.0000

. mixed ln_Eselectin i.time i.restricted pscore if per_protocol==1 ||RecordID:, mle

Mixed-effects ML regression Number of obs = 302

Group variable: RecordID Number of groups = 84

Obs per group:

min = 1

avg = 3.6

max = 4

Wald chi2(5) = 16.47

Log likelihood = -266.39759 Prob > chi2 = 0.0056

------------------------------------------------------------------------------

ln_Eselectin | Coefficient Std. err. z P>|z| [95% conf. interval]

-------------+----------------------------------------------------------------

time |

3 | -.1043812 .0626437 -1.67 0.096 -.2271605 .0183981

6 | -.0789313 .0627963 -1.26 0.209 -.2020098 .0441472

24 | -.2020726 .0624219 -3.24 0.001 -.3244172 -.0797279

|

restricted |

Restricted | .0369783 .2097457 0.18 0.860 -.3741156 .4480723

pscore | 2.519893 1.082244 2.33 0.020 .3987339 4.641052

_cons | 8.985293 .5790802 15.52 0.000 7.850317 10.12027

------------------------------------------------------------------------------

------------------------------------------------------------------------------

Random-effects parameters | Estimate Std. err. [95% conf. interval]

-----------------------------+------------------------------------------------

RecordID: Identity |

var(_cons) | .8407382 .1368652 .6110717 1.156723

-----------------------------+------------------------------------------------

var(Residual) | .1463125 .0140285 .1212462 .176561

------------------------------------------------------------------------------

LR test vs. linear model: chibar2(01) = 314.32 Prob >= chibar2 = 0.0000

# NGAL (censored)

. bysort restricted:tabstat NGALTn ln_NGAL if per_protocol==1, ///

> s(n mean sd median min max p25 p75) by(time) col(stats) long f(%6.4g)

-> restricted = Standard

time Variable | N Mean SD p50 Min Max p25 p75

-----------------+--------------------------------------------------------------------------

0 NGALTn | 39 525.2 664.5 274 .078 3392 137.4 568.7

ln_NGAL | 39 5.382 2.104 5.613 -2.551 8.129 4.923 6.343

-----------------+--------------------------------------------------------------------------

3 NGALTn | 39 403.5 489.8 249.4 53.05 2763 111 519.9

ln_NGAL | 39 5.553 .9321 5.519 3.971 7.924 4.71 6.254

-----------------+--------------------------------------------------------------------------

6 NGALTn | 34 348.6 290.1 257.4 86.14 1551 139 456.5

ln_NGAL | 34 5.586 .7373 5.551 4.456 7.347 4.934 6.124

-----------------+--------------------------------------------------------------------------

24 NGALTn | 34 375.3 384.4 229.8 71.77 1690 162.4 359.1

ln_NGAL | 34 5.597 .7646 5.437 4.273 7.432 5.09 5.884

-----------------+--------------------------------------------------------------------------

Total NGALTn | 146 416.7 485.9 257.8 .078 3392 140.4 493.2

ln_NGAL | 146 5.525 1.286 5.552 -2.551 8.129 4.944 6.201

--------------------------------------------------------------------------------------------

-> restricted = Restricted

time Variable | N Mean SD p50 Min Max p25 p75

----------------+--------------------------------------------------------------------------

0 NGALTn | 45 537.9 648.4 313.7 70.9 3756 158.3 661.3

ln_NGAL | 45 5.813 .9621 5.748 4.261 8.231 5.064 6.494

----------------+--------------------------------------------------------------------------

3 NGALTn | 42 393.9 409.4 213.8 52.28 1848 136.8 479.2

ln_NGAL | 42 5.549 .9289 5.357 3.957 7.522 4.919 6.172

----------------+--------------------------------------------------------------------------

6 NGALTn | 41 617.1 827 335 37.36 3605 148 594

ln_NGAL | 41 5.798 1.113 5.814 3.621 8.19 4.997 6.387

----------------+--------------------------------------------------------------------------

24 NGALTn | 44 427.3 451.6 279.8 41.56 2021 143.3 510.8

ln_NGAL | 44 5.628 .9392 5.634 3.727 7.611 4.964 6.234

----------------+--------------------------------------------------------------------------

Total NGALTn | 172 493.3 606.1 293 37.36 3756 146.3 559.8

ln_NGAL | 172 5.698 .9845 5.68 3.621 8.231 4.986 6.327

-------------------------------------------------------------------------------------------

. xttobit ln_NGAL i.time##restricted if per_protocol==1, ll(ln(0.0781)) nolog

Random-effects tobit regression Number of obs = 318

Uncensored = 316

Limits: Lower = ln(0.0781) Left-censored = 2

Upper = +inf Right-censored = 0

Group variable: RecordID Number of groups = 89

Random effects u_i ~ Gaussian Obs per group:

min = 1

avg = 3.6

max = 4

Integration method: mvaghermite Integration pts. = 12

Wald chi2(7) = 7.52

Log likelihood = -399.9948 Prob > chi2 = 0.3770

---------------------------------------------------------------------------------

ln_NGAL | Coefficient Std. err. z P>|z| [95% conf. interval]

----------------+----------------------------------------------------------------

time |

3 | .1736916 .1387116 1.25 0.211 -.0981781 .4455613

6 | .1763042 .1455475 1.21 0.226 -.1089637 .461572

24 | .1266501 .1466012 0.86 0.388 -.160683 .4139831

|

restricted |

Restricted | .4628044 .2500709 1.85 0.064 -.0273256 .9529344

|

time#restricted |

3#Restricted | -.3778371 .1920688 -1.97 0.049 -.7542849 -.0013892

6#Restricted | -.2189946 .197345 -1.11 0.267 -.6057838 .1677945

24#Restricted | -.3535653 .1964963 -1.80 0.072 -.738691 .0315605

|

_cons | 5.355257 .1833935 29.20 0.000 4.995812 5.714702

----------------+----------------------------------------------------------------

/sigma_u | .9890677 .0837505 11.81 0.000 .8249198 1.153216

/sigma_e | .6095664 .0288031 21.16 0.000 .5531134 .6660194

----------------+----------------------------------------------------------------

rho | .7247266 .0398999 .6415804 .7970685

---------------------------------------------------------------------------------

LR test of sigma_u=0: chibar2(01) = 184.41 Prob >= chibar2 = 0.000

. testparm time#restricted

( 1) [ln_NGAL]3.time#1.restricted = 0

( 2) [ln_NGAL]6.time#1.restricted = 0

( 3) [ln_NGAL]24.time#1.restricted = 0

chi2( 3) = 4.84

Prob > chi2 = 0.1836

. xttobit ln_NGAL i.time##restricted if per_protocol==1, ll(ln(0.0781)) nolog ///

> vce(bootstrap, reps(500) seed(010967))

(running xttobit on estimation sample)

Random-effects tobit regression Number of obs = 318

Uncensored = 316

Limits: Lower = ln(0.0781) Left-censored = 2

Upper = +inf Right-censored = 0

Replications = 500

Group variable: RecordID Number of groups = 89

Random effects u_i ~ Gaussian Obs per group:

min = 1

avg = 3.6

max = 4

Integration method: mvaghermite Integration pts. = 12

Wald chi2(7) = 16.06

Log likelihood = -399.9948 Prob > chi2 = 0.0246

(Replications based on 89 clusters in RecordID)

---------------------------------------------------------------------------------

| Observed Bootstrap Normal-based

ln_NGAL | coefficient std. err. z P>|z| [95% conf. interval]

----------------+----------------------------------------------------------------

time |

3 | .1736916 .2407668 0.72 0.471 -.2982027 .6455859

6 | .1763042 .2237484 0.79 0.431 -.2622347 .614843

24 | .1266501 .247912 0.51 0.609 -.3592486 .6125487

|

restricted |

Restricted | .4628044 .3415541 1.35 0.175 -.2066293 1.132238

|

time#restricted |

3#Restricted | -.3778371 .2520886 -1.50 0.134 -.8719216 .1162475

6#Restricted | -.2189946 .2389835 -0.92 0.359 -.6873938 .2494045

24#Restricted | -.3535653 .2590066 -1.37 0.172 -.8612089 .1540783

|

_cons | 5.355257 .3102173 17.26 0.000 4.747242 5.963272

----------------+----------------------------------------------------------------

/sigma_u | .9890677 .1050139 9.42 0.000 .7832442 1.194891

/sigma_e | .6095664 .1516093 4.02 0.000 .3124176 .9067152

----------------+----------------------------------------------------------------

rho | .7247266 .086484 .5355342 .8653537

---------------------------------------------------------------------------------

LR test of sigma_u=0: chibar2(01) = 184.41 Prob >= chibar2 = 0.000

. testparm time#restricted

( 1) [ln_NGAL]3.time#1.restricted = 0

( 2) [ln_NGAL]6.time#1.restricted = 0

( 3) [ln_NGAL]24.time#1.restricted = 0

chi2( 3) = 3.74

Prob > chi2 = 0.2911

Running the model again (bootstrapped) in the reduced sample before including the propensity score:

. xttobit ln_NGAL i.time##restricted if pscore~=. & per_protocol==1, ll(ln(0.0781)) nolog /// vce(bootstrap, reps(500) seed(010967))

(running xttobit on estimation sample)

Random-effects tobit regression Number of obs = 302

Uncensored = 300

Limits: Lower = ln(0.0781) Left-censored = 2

Upper = +inf Right-censored = 0

Replications = 500

Group variable: RecordID Number of groups = 84

Random effects u_i ~ Gaussian Obs per group:

min = 1

avg = 3.6

max = 4

Integration method: mvaghermite Integration pts. = 12

Wald chi2(7) = 14.12

Log likelihood = -385.59943 Prob > chi2 = 0.0490

(Replications based on 84 clusters in RecordID)

---------------------------------------------------------------------------------

| Observed Bootstrap Normal-based

ln_NGAL | coefficient std. err. z P>|z| [95% conf. interval]

----------------+----------------------------------------------------------------

time |

3 | .193685 .2597647 0.75 0.456 -.3154445 .7028144

6 | .1870786 .2455352 0.76 0.446 -.2941616 .6683188

24 | .1569105 .267476 0.59 0.557 -.3673328 .6811538

|

restricted |

Restricted | .4862804 .3713047 1.31 0.190 -.2414634 1.214024

|

time#restricted |

3#Restricted | -.3880595 .2699867 -1.44 0.151 -.9172236 .1411047

6#Restricted | -.2194766 .2600745 -0.84 0.399 -.7292133 .29026

24#Restricted | -.3869702 .2806739 -1.38 0.168 -.9370811 .1631406

|

_cons | 5.358415 .3424883 15.65 0.000 4.687151 6.02968

----------------+----------------------------------------------------------------

/sigma_u | 1.002574 .1122785 8.93 0.000 .7825125 1.222636

/sigma_e | .6227704 .1613432 3.86 0.000 .3065436 .9389972

----------------+----------------------------------------------------------------

rho | .7215768 .0931432 .5174371 .8710445

---------------------------------------------------------------------------------

LR test of sigma_u=0: chibar2(01) = 173.11 Prob >= chibar2 = 0.000

. testparm time#restricted

( 1) [ln_NGAL]3.time#1.restricted = 0

( 2) [ln_NGAL]6.time#1.restricted = 0

( 3) [ln_NGAL]24.time#1.restricted = 0

chi2( 3) = 3.94

Prob > chi2 = 0.2677

. xttobit ln_NGAL i.time##restricted pscore if per_protocol==1, ll(ln(0.0781)) nolog ///

> vce(bootstrap, reps(500) seed(010967))

(running xttobit on estimation sample)

Random-effects tobit regression Number of obs = 302

Uncensored = 300

Limits: Lower = ln(0.0781) Left-censored = 2

Upper = +inf Right-censored = 0

Replications = 500

Group variable: RecordID Number of groups = 84

Random effects u_i ~ Gaussian Obs per group:

min = 1

avg = 3.6

max = 4

Integration method: mvaghermite Integration pts. = 12

Wald chi2(8) = 32.07

Log likelihood = -379.66874 Prob > chi2 = 0.0001

(Replications based on 84 clusters in RecordID)

---------------------------------------------------------------------------------

| Observed Bootstrap Normal-based

ln_NGAL | coefficient std. err. z P>|z| [95% conf. interval]

----------------+----------------------------------------------------------------

time |

3 | .1973645 .2608714 0.76 0.449 -.3139341 .7086631

6 | .1966583 .251554 0.78 0.434 -.2963785 .6896951

24 | .1615029 .2718732 0.59 0.552 -.3713589 .6943646

|

restricted |

Restricted | .3359149 .3406383 0.99 0.324 -.331724 1.003554

|

time#restricted |

3#Restricted | -.388506 .2701543 -1.44 0.150 -.9179987 .1409866

6#Restricted | -.2311528 .2670866 -0.87 0.387 -.754633 .2923274

24#Restricted | -.3909916 .2839802 -1.38 0.169 -.9475826 .1655995

|

pscore | 4.045676 1.0439 3.88 0.000 1.999669 6.091684

_cons | 3.271892 .6948766 4.71 0.000 1.909959 4.633825

----------------+----------------------------------------------------------------

/sigma_u | .9221851 .1229267 7.50 0.000 .6812532 1.163117

/sigma_e | .6236071 .1621803 3.85 0.000 .3057396 .9414746

----------------+----------------------------------------------------------------

rho | .6862081 .1140993 .4421856 .8677228

---------------------------------------------------------------------------------

LR test of sigma_u=0: chibar2(01) = 146.29 Prob >= chibar2 = 0.000

. testparm time#restricted

( 1) [ln_NGAL]3.time#1.restricted = 0

( 2) [ln_NGAL]6.time#1.restricted = 0

( 3) [ln_NGAL]24.time#1.restricted = 0

chi2( 3) = 3.86

Prob > chi2 = 0.2769

. xttobit ln_NGAL i.time i.restricted if per_protocol==1 , ll(ln(0.0781)) nolog ///

> vce(bootstrap, reps(500) seed(010967))

(running xttobit on estimation sample)

Random-effects tobit regression Number of obs = 318

Uncensored = 316

Limits: Lower = ln(0.0781) Left-censored = 2

Upper = +inf Right-censored = 0

Replications = 500

Group variable: RecordID Number of groups = 89

Random effects u_i ~ Gaussian Obs per group:

min = 1

avg = 3.6

max = 4

Integration method: mvaghermite Integration pts. = 12

Wald chi2(4) = 5.60

Log likelihood = -402.39377 Prob > chi2 = 0.2309

(Replications based on 89 clusters in RecordID)

------------------------------------------------------------------------------

| Observed Bootstrap Normal-based

ln_NGAL | coefficient std. err. z P>|z| [95% conf. interval]

-------------+----------------------------------------------------------------

time |

3 | -.0244785 .1155382 -0.21 0.832 -.2509291 .2019721

6 | .0584814 .110723 0.53 0.597 -.1585316 .2754945

24 | -.0667922 .120543 -0.55 0.580 -.3030522 .1694678

|

restricted |

Restricted | .2284745 .2186664 1.04 0.296 -.2001037 .6570528

_cons | 5.480552 .2273018 24.11 0.000 5.035048 5.926055

-------------+----------------------------------------------------------------

/sigma_u | .9889491 .1053148 9.39 0.000 .782536 1.195362

/sigma_e | .6157324 .1570356 3.92 0.000 .3079483 .9235165

-------------+----------------------------------------------------------------

rho | .7206445 .0899209 .5241665 .8662635

------------------------------------------------------------------------------

LR test of sigma_u=0: chibar2(01) = 181.58 Prob >= chibar2 = 0.000

. xttobit ln_NGAL i.time i.restricted pscore if per_protocol==1, ll(ln(0.0781)) nolog ///

> vce(bootstrap, reps(500) seed(010967))

(running xttobit on estimation sample)

Random-effects tobit regression Number of obs = 302

Uncensored = 300

Limits: Lower = ln(0.0781) Left-censored = 2

Upper = +inf Right-censored = 0

Replications = 500

Group variable: RecordID Number of groups = 84

Random effects u_i ~ Gaussian Obs per group:

min = 1

avg = 3.6

max = 4

Integration method: mvaghermite Integration pts. = 12

Wald chi2(5) = 24.20

Log likelihood = -382.13395 Prob > chi2 = 0.0002

(Replications based on 84 clusters in RecordID)

------------------------------------------------------------------------------

| Observed Bootstrap Normal-based

ln_NGAL | coefficient std. err. z P>|z| [95% conf. interval]

-------------+----------------------------------------------------------------

time |

3 | -.0020523 .1305668 -0.02 0.987 -.2579586 .2538539

6 | .0748627 .1272787 0.59 0.556 -.1745989 .3243243

24 | -.0489552 .1352996 -0.36 0.717 -.3141375 .2162271

|

restricted |

Restricted | .0886298 .220395 0.40 0.688 -.3433365 .5205961

pscore | 4.047581 1.0382 3.90 0.000 2.012746 6.082416

_cons | 3.399717 .6249068 5.44 0.000 2.174923 4.624512

-------------+----------------------------------------------------------------

/sigma_u | .9226826 .1237262 7.46 0.000 .6801838 1.165182

/sigma_e | .6302465 .1685321 3.74 0.000 .2999295 .9605634

-------------+----------------------------------------------------------------

rho | .6818635 .1180564 .430262 .8689708

------------------------------------------------------------------------------

LR test of sigma_u=0: chibar2(01) = 143.73 Prob >= chibar2 = 0.000

# Resisitin (censored values)

. bysort restricted:tabstat ResistinTn ln_Resistin if per_protocol==1, ///

> s(n mean sd median min max p25 p75) by(time) col(stats) long f(%6.4g)

-> restricted = Standard

time Variable | N Mean SD p50 Min Max p25 p75

------------------+-------------------------------------------------------------------------

0 ResistinTn | 39 142.4 143 101.8 .0031 658.2 54.34 141.3

ln_Resistin | 39 4.341 1.879 4.623 -5.77 6.49 3.995 4.951

------------------+-------------------------------------------------------------------------

3 ResistinTn | 39 205.5 346.4 117 .0031 1600 48.87 175.4

ln_Resistin | 39 4.478 1.954 4.762 -5.77 7.378 3.889 5.167

------------------+-------------------------------------------------------------------------

6 ResistinTn | 35 140.8 149.2 100.8 .0031 729.3 55.65 152.8

ln_Resistin | 35 4.34 1.929 4.613 -5.77 6.592 4.019 5.029

------------------+-------------------------------------------------------------------------

24 ResistinTn | 35 131.1 164.7 73.72 .0031 810.5 35.01 162.3

ln_Resistin | 35 4.141 1.955 4.3 -5.77 6.698 3.556 5.089

------------------+-------------------------------------------------------------------------

Total ResistinTn |148 156 220.5 105.7 .0031 1600 46.75 157.6

ln_Resistin |148 4.329 1.913 4.661 -5.77 7.378 3.844 5.06

--------------------------------------------------------------------------------------------

-> restricted = Restricted

time Variable | N Mean SD p50 Min Max p25 p75

-----------------+-------------------------------------------------------------------------

0 ResistinTn | 45 121 207.3 71.03 .0031 1350 30.81 118.6

ln_Resistin | 45 4.034 1.789 4.263 -5.77 7.208 3.428 4.776

-----------------+-------------------------------------------------------------------------

3 ResistinTn | 43 176.3 327.6 94.41 16.81 1600 40.73 168.9

ln_Resistin | 43 4.497 1.052 4.548 2.822 7.378 3.707 5.129

-----------------+-------------------------------------------------------------------------

6 ResistinTn | 40 152.7 256.2 105.8 10.82 1600 36.49 177.6

ln_Resistin | 40 4.418 1.074 4.662 2.381 7.378 3.596 5.177

-----------------+-------------------------------------------------------------------------

24 ResistinTn | 44 129.3 165.6 87.91 13.68 811.9 35.72 136.4

ln_Resistin | 44 4.307 1.06 4.476 2.616 6.699 3.575 4.915

-----------------+-------------------------------------------------------------------------

Total ResistinTn |172 144.3 244.5 88.53 .0031 1600 37.19 149.8

ln_Resistin |172 4.309 1.294 4.483 -5.77 7.378 3.616 5.01

-------------------------------------------------------------------------------------------

. xttobit ln_Resistin i.time##restricted if per_protocol==1, ll(ln(0.00312001)) ul(ln(1600)) nolog

Random-effects tobit regression Number of obs = 320

Uncensored = 310

Limits: Lower = ln(0.00312001) Left-censored = 5

Upper = ln(1600) Right-censored = 5

Group variable: RecordID Number of groups = 89

Random effects u_i ~ Gaussian Obs per group:

min = 1

avg = 3.6

max = 4

Integration method: mvaghermite Integration pts. = 12

Wald chi2(7) = 8.80

Log likelihood = -540.16533 Prob > chi2 = 0.2670

---------------------------------------------------------------------------------

ln_Resistin | Coefficient Std. err. z P>|z| [95% conf. interval]

----------------+----------------------------------------------------------------

time |

3 | .1650137 .2302912 0.72 0.474 -.2863487 .6163762

6 | .0073418 .238283 0.03 0.975 -.4596843 .4743678

24 | -.199398 .2397078 -0.83 0.406 -.6692167 .2704207

|

restricted |

Restricted | -.2801166 .3493671 -0.80 0.423 -.9648635 .4046304

|

time#restricted |

3#Restricted | .3768158 .317605 1.19 0.235 -.2456786 .9993101

6#Restricted | .356159 .3261677 1.09 0.275 -.2831181 .995436

24#Restricted | .4301144 .3228684 1.33 0.183 -.202696 1.062925

|

_cons | 4.32237 .2560618 16.88 0.000 3.820498 4.824242

----------------+----------------------------------------------------------------

/sigma_u | 1.262892 .1117087 11.31 0.000 1.043947 1.481837

/sigma_e | 1.008792 .047702 21.15 0.000 .9152981 1.102286

----------------+----------------------------------------------------------------

rho | .6104729 .0489446 .5121419 .7021741

---------------------------------------------------------------------------------

LR test of sigma_u=0: chibar2(01) = 144.20 Prob >= chibar2 = 0.000

. testparm time#restricted

( 1) [ln_Resistin]3.time#1.restricted = 0

( 2) [ln_Resistin]6.time#1.restricted = 0

( 3) [ln_Resistin]24.time#1.restricted = 0

chi2( 3) = 2.28

Prob > chi2 = 0.5166

. xttobit ln_Resistin i.time##restricted if per_protocol==1, ll(ln(0.00312001)) ul(ln(1600)) nolog ///

> vce(bootstrap, reps(500) seed(010967)) nodots

Random-effects tobit regression Number of obs = 320

Uncensored = 310

Limits: Lower = ln(0.00312001) Left-censored = 5

Upper = ln(1600) Right-censored = 5

Replications = 500

Group variable: RecordID Number of groups = 89

Random effects u_i ~ Gaussian Obs per group:

min = 1

avg = 3.6

max = 4

Integration method: mvaghermite Integration pts. = 12

Wald chi2(7) = 23.03

Log likelihood = -540.16533 Prob > chi2 = 0.0017

(Replications based on 89 clusters in RecordID)

---------------------------------------------------------------------------------

| Observed Bootstrap Normal-based

ln_Resistin | coefficient std. err. z P>|z| [95% conf. interval]

----------------+----------------------------------------------------------------

time |

3 | .1650137 .3777585 0.44 0.662 -.5753794 .9054069

6 | .0073418 .3894602 0.02 0.985 -.7559862 .7706698

24 | -.199398 .4071052 -0.49 0.624 -.9973094 .5985134

|

restricted |

Restricted | -.2801166 .4163751 -0.67 0.501 -1.096197 .5359637

|

time#restricted |

3#Restricted | .3768158 .4397774 0.86 0.392 -.485132 1.238764

6#Restricted | .356159 .4474736 0.80 0.426 -.5208731 1.233191

24#Restricted | .4301144 .4699917 0.92 0.360 -.4910525 1.351281

|

_cons | 4.32237 .3015634 14.33 0.000 3.731316 4.913423

----------------+----------------------------------------------------------------

/sigma_u | 1.262892 .246328 5.13 0.000 .7800977 1.745686

/sigma_e | 1.008792 .2529928 3.99 0.000 .5129355 1.504649

----------------+----------------------------------------------------------------

rho | .6104729 .0775945 .4538406 .7508184

---------------------------------------------------------------------------------

LR test of sigma_u=0: chibar2(01) = 144.20 Prob >= chibar2 = 0.000

. testparm time#restricted

( 1) [ln_Resistin]3.time#1.restricted = 0

( 2) [ln_Resistin]6.time#1.restricted = 0

( 3) [ln_Resistin]24.time#1.restricted = 0

chi2( 3) = 1.01

Prob > chi2 = 0.7984

. xttobit ln_Resistin i.time##restricted if pscore~=. & per_protocol==1, ///

> ll(ln(0.00312001)) ul(ln(1600)) nolog ///

> vce(bootstrap, reps(500) seed(010967)) nodots

Random-effects tobit regression Number of obs = 304

Uncensored = 294

Limits: Lower = ln(0.00312001) Left-censored = 5

Upper = ln(1600) Right-censored = 5

Replications = 500

Group variable: RecordID Number of groups = 84

Random effects u_i ~ Gaussian Obs per group:

min = 1

avg = 3.6

max = 4

Integration method: mvaghermite Integration pts. = 12

Wald chi2(7) = 19.15

Log likelihood = -520.01763 Prob > chi2 = 0.0077

(Replications based on 84 clusters in RecordID)

---------------------------------------------------------------------------------

| Observed Bootstrap Normal-based

ln_Resistin | coefficient std. err. z P>|z| [95% conf. interval]

----------------+----------------------------------------------------------------

time |

3 | .1667501 .3938846 0.42 0.672 -.6052495 .9387497

6 | .0099756 .4151585 0.02 0.981 -.8037202 .8236713

24 | -.1700691 .4408954 -0.39 0.700 -1.034208 .69407

|

restricted |

Restricted | -.2703063 .411832 -0.66 0.512 -1.077482 .5368696

|

time#restricted |

3#Restricted | .4087662 .4447361 0.92 0.358 -.4629004 1.280433

6#Restricted | .3876529 .4529458 0.86 0.392 -.5001046 1.27541

24#Restricted | .4356895 .4760587 0.92 0.360 -.4973684 1.368747

|

_cons | 4.317773 .3054988 14.13 0.000 3.719006 4.916539

----------------+----------------------------------------------------------------

/sigma_u | 1.295168 .2592371 5.00 0.000 .7870727 1.803263

/sigma_e | 1.033436 .2723033 3.80 0.000 .4997311 1.567141

----------------+----------------------------------------------------------------

rho | .6109965 .0784185 .4526523 .7526326

---------------------------------------------------------------------------------

LR test of sigma_u=0: chibar2(01) = 136.97 Prob >= chibar2 = 0.000

. testparm time#restricted

( 1) [ln_Resistin]3.time#1.restricted = 0

( 2) [ln_Resistin]6.time#1.restricted = 0

( 3) [ln_Resistin]24.time#1.restricted = 0

chi2( 3) = 1.00

Prob > chi2 = 0.8013

. xttobit ln_Resistin i.time##restricted pscore if per_protocol==1, ///

> ll(ln(0.00312001)) ul(ln(1600)) nolog ///

> vce(bootstrap, reps(500) seed(010967)) nodots

Random-effects tobit regression Number of obs = 304

Uncensored = 294

Limits: Lower = ln(0.00312001) Left-censored = 5

Upper = ln(1600) Right-censored = 5

Replications = 500

Group variable: RecordID Number of groups = 84

Random effects u_i ~ Gaussian Obs per group:

min = 1

avg = 3.6

max = 4

Integration method: mvaghermite Integration pts. = 12

Wald chi2(8) = 25.57

Log likelihood = -511.55418 Prob > chi2 = 0.0012

(Replications based on 84 clusters in RecordID)

---------------------------------------------------------------------------------

| Observed Bootstrap Normal-based

ln_Resistin | coefficient std. err. z P>|z| [95% conf. interval]

----------------+----------------------------------------------------------------

time |

3 | .1755643 .3930971 0.45 0.655 -.5948918 .9460203

6 | .0269553 .4133437 0.07 0.948 -.7831835 .8370941

24 | -.1715677 .4406416 -0.39 0.697 -1.035209 .6920741

|

restricted |

Restricted | -.5066291 .4221335 -1.20 0.230 -1.333996 .3207373

|

time#restricted |

3#Restricted | .4074359 .4446036 0.92 0.359 -.4639712 1.278843

6#Restricted | .3667198 .4486881 0.82 0.414 -.5126927 1.246132

24#Restricted | .4394124 .4763569 0.92 0.356 -.49423 1.373055

|

pscore | 6.361341 2.484216 2.56 0.010 1.492367 11.23032

_cons | 1.036547 1.314575 0.79 0.430 -1.539973 3.613066

----------------+----------------------------------------------------------------

/sigma_u | 1.146853 .1733611 6.62 0.000 .8070714 1.486634

/sigma_e | 1.03375 .2723458 3.80 0.000 .4999625 1.567538

----------------+----------------------------------------------------------------

rho | .5517283 .0905703 .3749727 .7186278

---------------------------------------------------------------------------------

LR test of sigma_u=0: chibar2(01) = 108.44 Prob >= chibar2 = 0.000

. testparm time#restricted

( 1) [ln_Resistin]3.time#1.restricted = 0

( 2) [ln_Resistin]6.time#1.restricted = 0

( 3) [ln_Resistin]24.time#1.restricted = 0

chi2( 3) = 1.20

Prob > chi2 = 0.7537

. xttobit ln_Resistin i.time i.restricted if per_protocol==1 , ///

> ll(ln(0.00312001)) ul(ln(1600)) nolog ///

> vce(bootstrap, reps(500) seed(010967)) nodots

Random-effects tobit regression Number of obs = 320

Uncensored = 310

Limits: Lower = ln(0.00312001) Left-censored = 5

Upper = ln(1600) Right-censored = 5

Replications = 500

Group variable: RecordID Number of groups = 89

Random effects u_i ~ Gaussian Obs per group:

min = 1

avg = 3.6

max = 4

Integration method: mvaghermite Integration pts. = 12

Wald chi2(4) = 20.52

Log likelihood = -541.29976 Prob > chi2 = 0.0004

(Replications based on 89 clusters in RecordID)

------------------------------------------------------------------------------

| Observed Bootstrap Normal-based

ln_Resistin | coefficient std. err. z P>|z| [95% conf. interval]

-------------+----------------------------------------------------------------

time |

3 | .3647201 .2078568 1.75 0.079 -.0426718 .7721119

6 | .1991105 .207114 0.96 0.336 -.2068255 .6050465

24 | .0357973 .2214506 0.16 0.872 -.3982378 .4698324

|

restricted |

Restricted | .00441 .2945856 0.01 0.988 -.5729673 .5817872

_cons | 4.17005 .2479588 16.82 0.000 3.684059 4.65604

-------------+----------------------------------------------------------------

/sigma_u | 1.262569 .2457586 5.14 0.000 .7808908 1.744247

/sigma_e | 1.013575 .2571485 3.94 0.000 .5095733 1.517577

-------------+----------------------------------------------------------------

rho | .6080991 .0771845 .4524887 .7479691

------------------------------------------------------------------------------

LR test of sigma_u=0: chibar2(01) = 142.95 Prob >= chibar2 = 0.000

. xttobit ln_Resistin i.time i.restricted pscore if per_protocol==1, ///

> ll(ln(0.00312001)) ul(ln(1600)) nolog ///

> vce(bootstrap, reps(500) seed(010967)) nodots

Random-effects tobit regression Number of obs = 304

Uncensored = 294

Limits: Lower = ln(0.00312001) Left-censored = 5

Upper = ln(1600) Right-censored = 5

Replications = 500

Group variable: RecordID Number of groups = 84

Random effects u_i ~ Gaussian Obs per group:

min = 1

avg = 3.6

max = 4

Integration method: mvaghermite Integration pts. = 12

Wald chi2(5) = 23.80

Log likelihood = -512.67313 Prob > chi2 = 0.0002

(Replications based on 84 clusters in RecordID)

------------------------------------------------------------------------------

| Observed Bootstrap Normal-based

ln_Resistin | coefficient std. err. z P>|z| [95% conf. interval]

-------------+----------------------------------------------------------------

time |

3 | .3868728 .2285362 1.69 0.090 -.0610498 .8347955

6 | .2207955 .2340268 0.94 0.345 -.2378887 .6794796

24 | .0642475 .2496619 0.26 0.797 -.4250809 .5535758

|

restricted |

Restricted | -.2112516 .2705821 -0.78 0.435 -.7415828 .3190795

pscore | 6.368645 2.49279 2.55 0.011 1.482867 11.25442

_cons | .8782626 1.385599 0.63 0.526 -1.837461 3.593986

-------------+----------------------------------------------------------------

/sigma_u | 1.145678 .1715501 6.68 0.000 .8094462 1.48191

/sigma_e | 1.038995 .2769207 3.75 0.000 .4962402 1.581749

-------------+----------------------------------------------------------------

rho | .5487166 .0902457 .3728617 .7153634

------------------------------------------------------------------------------

LR test of sigma_u=0: chibar2(01) = 107.25 Prob >= chibar2 = 0.000

# Syn 1 (censored values)

. bysort restricted:tabstat Syn1Tn ln_Syn1 if per_protocol==1, ///

> s(n mean sd median min max p25 p75) by(time) col(stats) long f(%8.0g)

-> restricted = Standard

time Variable | N Mean SD p50 Min Max p25 p75

------------------+-------------------------------------------------------------------------

0 Syn1Tn | 39 17817.3 24946.6 6913 125 112800 4804 18820

ln_Syn1 | 39 9.1222 1.22248 8.84116 4.82831 11.6334 8.4772 9.84268

------------------+-------------------------------------------------------------------------

3 Syn1Tn | 39 16723.2 25316.6 6203 2030 104400 3883 18260

ln_Syn1 | 39 9.0898 1.01968 8.73279 7.61579 11.556 8.26436 9.81247

------------------+-------------------------------------------------------------------------

6 Syn1Tn | 34 16020.6 22588.3 5657.5 2400 83260 3897 16060

ln_Syn1 | 34 9.04755 1.04146 8.64035 7.78322 11.3297 8.26796 9.68409

------------------+-------------------------------------------------------------------------

24 Syn1Tn | 35 18572.3 29410.9 7681 125 141000 5060 12960

ln_Syn1 | 35 9.08912 1.24305 8.9465 4.82831 11.8565 8.52912 9.46962

------------------+-------------------------------------------------------------------------

Total Syn1Tn |147 17291.2 25426.2 6866 125 141000 4425 16710

ln_Syn1 |147 9.08846 1.12484 8.83434 4.82831 11.8565 8.39503 9.72376

--------------------------------------------------------------------------------------------

-> restricted = Restricted

time Variable | N Mean SD p50 Min Max p25 p75

-----------------+-------------------------------------------------------------------------

0 Syn1Tn | 45 19544.3 36869.9 5337 1305 171100 3255 15030

ln_Syn1 | 45 8.9453 1.23822 8.58242 7.17396 12.05 8.08795 9.6178

-----------------+-------------------------------------------------------------------------

3 Syn1Tn | 43 18136 34411 5878 1709 154400 2861 14610

ln_Syn1 | 43 8.88635 1.18464 8.67897 7.44366 11.9473 7.95893 9.58946

-----------------+-------------------------------------------------------------------------

6 Syn1Tn | 40 15166.6 34758 4994 1592 186300 2818 10870

ln_Syn1 | 40 8.75254 1.08437 8.51587 7.37275 12.1351 7.94367 9.29376

-----------------+-------------------------------------------------------------------------

24 Syn1Tn | 43 22364.3 43840.4 6866 1360 226800 4664 14970

ln_Syn1 | 43 9.13047 1.16662 8.83434 7.21524 12.3318 8.44763 9.6138

-----------------+-------------------------------------------------------------------------

Total Syn1Tn |171 18875.3 37456.1 5753 1305 226800 3255 14350

ln_Syn1 |171 8.93195 1.16947 8.65748 7.17396 12.3318 8.08795 9.57151

-------------------------------------------------------------------------------------------

. xttobit ln_Syn1 i.time##restricted if per_protocol==1, ll(ln(125.0001)) ul(ln(1280000)) nolog

Random-effects tobit regression Number of obs = 318

Uncensored = 316

Limits: Lower = ln(125.0001) Left-censored = 2

Upper = ln(1280000) Right-censored = 0

Group variable: RecordID Number of groups = 89

Random effects u_i ~ Gaussian Obs per group:

min = 1

avg = 3.6

max = 4

Integration method: mvaghermite Integration pts. = 12

Wald chi2(7) = 6.05

Log likelihood = -315.15543 Prob > chi2 = 0.5341

---------------------------------------------------------------------------------

ln_Syn1 | Coefficient Std. err. z P>|z| [95% conf. interval]

----------------+----------------------------------------------------------------

time |

3 | -.0419583 .0942413 -0.45 0.656 -.2266678 .1427512

6 | -.0788031 .0990037 -0.80 0.426 -.2728467 .1152405

24 | .0046376 .0988611 0.05 0.963 -.1891267 .1984019

|

restricted |

Restricted | -.0951694 .245925 -0.39 0.699 -.5771735 .3868347

|

time#restricted |

3#Restricted | -.0605467 .1300503 -0.47 0.642 -.3154406 .1943472

6#Restricted | -.0628789 .134734 -0.47 0.641 -.3269527 .2011949

24#Restricted | .0320876 .1335168 0.24 0.810 -.2296006 .2937757

|

_cons | 9.090742 .1804819 50.37 0.000 8.737004 9.44448

----------------+----------------------------------------------------------------

/sigma_u | 1.072361 .084019 12.76 0.000 .9076866 1.237035

/sigma_e | .4139005 .0194793 21.25 0.000 .3757218 .4520791

----------------+----------------------------------------------------------------

rho | .8703418 .0208572 .8249548 .9068498

---------------------------------------------------------------------------------

LR test of sigma_u=0: chibar2(01) = 361.04 Prob >= chibar2 = 0.000

. testparm time#restricted

( 1) [ln_Syn1]3.time#1.restricted = 0

( 2) [ln_Syn1]6.time#1.restricted = 0

( 3) [ln_Syn1]24.time#1.restricted = 0

chi2( 3) = 0.71

Prob > chi2 = 0.8715

. xttobit ln_Syn1 i.time##restricted if per_protocol==1, ll(ln(125.0001)) ul(ln(1280000)) nolog ///

> vce(bootstrap, reps(500) seed(010967))

(running xttobit on estimation sample)

Random-effects tobit regression Number of obs = 318

Uncensored = 316

Limits: Lower = ln(125.0001) Left-censored = 2

Upper = ln(1280000) Right-censored = 0

Replications = 500

Group variable: RecordID Number of groups = 89

Random effects u_i ~ Gaussian Obs per group:

min = 1

avg = 3.6

max = 4

Integration method: mvaghermite Integration pts. = 12

Wald chi2(7) = 9.59

Log likelihood = -315.15543 Prob > chi2 = 0.2131

(Replications based on 89 clusters in RecordID)

---------------------------------------------------------------------------------

| Observed Bootstrap Normal-based

ln_Syn1 | coefficient std. err. z P>|z| [95% conf. interval]

----------------+----------------------------------------------------------------

time |

3 | -.0419583 .1005157 -0.42 0.676 -.2389655 .1550489

6 | -.0788031 .1082275 -0.73 0.467 -.290925 .1333189

24 | .0046376 .0919188 0.05 0.960 -.17552 .1847952

|

restricted |

Restricted | -.0951694 .278371 -0.34 0.732 -.6407665 .4504277

|

time#restricted |

3#Restricted | -.0605467 .1536744 -0.39 0.694 -.3617431 .2406497

6#Restricted | -.0628789 .1564779 -0.40 0.688 -.3695699 .2438121

24#Restricted | .0320876 .1399838 0.23 0.819 -.2422757 .3064508

|

_cons | 9.090742 .2086196 43.58 0.000 8.681855 9.499629

----------------+----------------------------------------------------------------

/sigma_u | 1.072361 .0975251 11.00 0.000 .881215 1.263506

/sigma_e | .4139005 .0753325 5.49 0.000 .2662514 .5615496

----------------+----------------------------------------------------------------

rho | .8703418 .0436663 .7650687 .9374008

---------------------------------------------------------------------------------

LR test of sigma_u=0: chibar2(01) = 361.04 Prob >= chibar2 = 0.000

. testparm time#restricted

( 1) [ln_Syn1]3.time#1.restricted = 0

( 2) [ln_Syn1]6.time#1.restricted = 0

( 3) [ln_Syn1]24.time#1.restricted = 0

chi2( 3) = 0.53

Prob > chi2 = 0.9128

. xttobit ln_Syn1 i.time##restricted pscore if per_protocol==1, ll(ln(125.0001)) nolog ///

> vce(bootstrap, reps(500) seed(010967))

(running xttobit on estimation sample)

Random-effects tobit regression Number of obs = 302

Uncensored = 300

Limits: Lower = ln(125.0001) Left-censored = 2

Upper = +inf Right-censored = 0

Replications = 500

Group variable: RecordID Number of groups = 84

Random effects u_i ~ Gaussian Obs per group:

min = 1

avg = 3.6

max = 4

Integration method: mvaghermite Integration pts. = 12

Wald chi2(8) = 10.50

Log likelihood = -304.53353 Prob > chi2 = 0.2318

(Replications based on 84 clusters in RecordID)

---------------------------------------------------------------------------------

| Observed Bootstrap Normal-based

ln_Syn1 | coefficient std. err. z P>|z| [95% conf. interval]

----------------+----------------------------------------------------------------

time |

3 | -.0496449 .096369 -0.52 0.606 -.2385247 .1392349

6 | -.0760997 .1054719 -0.72 0.471 -.2828209 .1306215

24 | .0063281 .0974823 0.06 0.948 -.1847337 .19739

|

restricted |

Restricted | -.0710018 .3100439 -0.23 0.819 -.6786768 .5366731

|

time#restricted |

3#Restricted | -.0536952 .1560271 -0.34 0.731 -.3595026 .2521123

6#Restricted | -.0629331 .1567297 -0.40 0.688 -.3701176 .2442513

24#Restricted | .0399045 .1562426 0.26 0.798 -.2663253 .3461343

|

pscore | -1.678828 1.584061 -1.06 0.289 -4.783531 1.425875

_cons | 9.986218 .8399985 11.89 0.000 8.339851 11.63259

----------------+----------------------------------------------------------------

/sigma_u | 1.07846 .0972192 11.09 0.000 .8879136 1.269006

/sigma_e | .4235931 .075586 5.60 0.000 .2754472 .571739

----------------+----------------------------------------------------------------

rho | .8663462 .0442652 .7601968 .9346863

---------------------------------------------------------------------------------

LR test of sigma_u=0: chibar2(01) = 337.65 Prob >= chibar2 = 0.000

. testparm time#restricted

( 1) [ln_Syn1]3.time#1.restricted = 0

( 2) [ln_Syn1]6.time#1.restricted = 0

( 3) [ln_Syn1]24.time#1.restricted = 0

chi2( 3) = 0.53

Prob > chi2 = 0.9133

. xttobit ln_Syn1 i.time i.restricted if per_protocol==1 , ll(ln(125.0001)) nolog ///

> vce(bootstrap, reps(500) seed(010967))

(running xttobit on estimation sample)

Random-effects tobit regression Number of obs = 318

Uncensored = 316

Limits: Lower = ln(125.0001) Left-censored = 2

Upper = +inf Right-censored = 0

Replications = 500

Group variable: RecordID Number of groups = 89

Random effects u_i ~ Gaussian Obs per group:

min = 1

avg = 3.6

max = 4

Integration method: mvaghermite Integration pts. = 12

Wald chi2(4) = 4.38

Log likelihood = -315.50884 Prob > chi2 = 0.3571

(Replications based on 89 clusters in RecordID)

------------------------------------------------------------------------------

| Observed Bootstrap Normal-based

ln_Syn1 | coefficient std. err. z P>|z| [95% conf. interval]

-------------+----------------------------------------------------------------

time |

3 | -.073663 .0774319 -0.95 0.341 -.2254268 .0781007

6 | -.1120977 .0767442 -1.46 0.144 -.2625136 .0383182

24 | .0227802 .0745153 0.31 0.760 -.1232672 .1688276

|

restricted |

Restricted | -.1166713 .2395701 -0.49 0.626 -.58622 .3528774

_cons | 9.102078 .1950695 46.66 0.000 8.719749 9.484407

-------------+----------------------------------------------------------------

/sigma_u | 1.07334 .0977147 10.98 0.000 .8818232 1.264858

/sigma_e | .4143574 .0764855 5.42 0.000 .2644487 .5642662

-------------+----------------------------------------------------------------

rho | .8702989 .0440927 .7638172 .9378503

------------------------------------------------------------------------------

LR test of sigma_u=0: chibar2(01) = 361.26 Prob >= chibar2 = 0.000

. xttobit ln_Syn1 i.time i.restricted pscore if per_protocol==1, ll(ln(125.0001)) nolog ///

> vce(bootstrap, reps(500) seed(010967))

(running xttobit on estimation sample)

Random-effects tobit regression Number of obs = 302

Uncensored = 300

Limits: Lower = ln(125.0001) Left-censored = 2

Upper = +inf Right-censored = 0

Replications = 500

Group variable: RecordID Number of groups = 84

Random effects u_i ~ Gaussian Obs per group:

min = 1

avg = 3.6

max = 4

Integration method: mvaghermite Integration pts. = 12

Wald chi2(5) = 5.77

Log likelihood = -304.87147 Prob > chi2 = 0.3293

(Replications based on 84 clusters in RecordID)

------------------------------------------------------------------------------

| Observed Bootstrap Normal-based

ln_Syn1 | coefficient std. err. z P>|z| [95% conf. interval]

-------------+----------------------------------------------------------------

time |

3 | -.0771129 .0781467 -0.99 0.324 -.2302777 .0760519

6 | -.1090053 .0770145 -1.42 0.157 -.259951 .0419403

24 | .0283159 .077801 0.36 0.716 -.1241712 .1808031

|

restricted |

Restricted | -.0894057 .2783206 -0.32 0.748 -.6349041 .4560928

pscore | -1.682969 1.585862 -1.06 0.289 -4.791201 1.425263

_cons | 9.998249 .8485228 11.78 0.000 8.335175 11.66132

-------------+----------------------------------------------------------------

/sigma_u | 1.079299 .0974246 11.08 0.000 .8883505 1.270248

/sigma_e | .4240849 .0767399 5.53 0.000 .2736774 .5744925

-------------+----------------------------------------------------------------

rho | .8662576 .0447241 .7588285 .9351401

------------------------------------------------------------------------------

LR test of sigma_u=0: chibar2(01) = 337.72 Prob >= chibar2 = 0.000

# Hyaluronan (censored values)

. bysort restricted:tabstat HyaluronanTn ln_Hyaluronan if per_protocol==1, ///

> s(n mean sd median min max p25 p75) by(time) col(stats) long f(%7.0g)

-> restricted = Standard

time Variable | N Mean SD p50 Min Max p25 p75

--------------------+-----------------------------------------------------------------------

0 HyaluronanTn | 39 965.95 1776.8 244.8 19.29 8705 81.43 1117

ln_Hyaluro~n | 39 5.6185 1.638 5.5004 2.9596 9.0717 4.3997 7.0184

--------------------+-----------------------------------------------------------------------

3 HyaluronanTn | 39 1038.2 2854.1 203.7 17.487 13765 80.57 589.8

ln_Hyaluro~n | 39 5.4481 1.5363 5.3166 2.8615 9.5299 4.3891 6.3798

--------------------+-----------------------------------------------------------------------

6 HyaluronanTn | 35 738.34 2205.4 143.22 22.194 12760 57.042 496.86

ln_Hyaluro~n | 35 5.1905 1.4547 4.9644 3.0998 9.4541 4.0438 6.2083

--------------------+-----------------------------------------------------------------------

24 HyaluronanTn | 35 1548.5 6772.7 137.82 .37 40120 42.69 374.94

ln_Hyaluro~n | 35 4.7056 2.3579 4.9259 -.99425 10.6 3.754 5.9268

--------------------+-----------------------------------------------------------------------

Total HyaluronanTn |148 1068.9 3839.3 153.24 .37 40120 72.56 559.22

ln_Hyaluro~n |148 5.2565 1.788 5.032 -.99425 10.6 4.2843 6.3262

--------------------------------------------------------------------------------------------

-> restricted = Restricted

time Variable | N Mean SD p50 Min Max p25 p75

-------------------+-------------------------------------------------------------------------

0 HyaluronanTn | 44 1064.5 2737.8 245.35 .37 17312 67.285 704.8

ln_Hyaluro~n | 44 5.2632 2.2928 5.5006 -.99425 9.7592 4.2031 6.5579

-------------------+-------------------------------------------------------------------------

3 HyaluronanTn | 43 510 1633.6 144.6 .37 10725 38.91 374.7

ln_Hyaluro~n | 43 4.56 2.2324 4.974 -.99425 9.2803 3.6613 5.9261

-------------------+-------------------------------------------------------------------------

6 HyaluronanTn | 41 567.06 2276.8 117.48 .37 14676 32.67 269.4

ln_Hyaluro~n | 41 4.2882 2.3696 4.7663 -.99425 9.594 3.4865 5.5962

-------------------+-------------------------------------------------------------------------

24 HyaluronanTn | 44 996.28 3252.1 165.96 .37 18485 38.666 548.7

ln_Hyaluro~n | 44 4.801 2.4009 5.111 -.99425 9.8247 3.6493 6.3057

-------------------+-------------------------------------------------------------------------

Total HyaluronanTn |172 789.85 2544.5 144.63 .37 18485 44.943 471.43

ln_Hyaluro~n |172 4.7367 2.3318 4.9742 -.99425 9.8247 3.8054 6.1554

---------------------------------------------------------------------------------------------

. xttobit ln_Hyaluronan b24.time##restricted if per_protocol==1, ll(ln(0.370001)) nolog

Random-effects tobit regression Number of obs = 320

Uncensored = 301

Limits: Lower = ln(0.370001) Left-censored = 19

Upper = +inf Right-censored = 0

Group variable: RecordID Number of groups = 89

Random effects u_i ~ Gaussian Obs per group:

min = 1

avg = 3.6

max = 4

Integration method: mvaghermite Integration pts. = 12

Wald chi2(7) = 26.43

Log likelihood = -601.75091 Prob > chi2 = 0.0004

---------------------------------------------------------------------------------

ln_Hyaluronan | Coefficient Std. err. z P>|z| [95% conf. interval]

----------------+----------------------------------------------------------------

time |

0 | .8305479 .283424 2.93 0.003 .2750471 1.386049

3 | .6663689 .283679 2.35 0.019 .1103683 1.22237

6 | .5062017 .2894835 1.75 0.080 -.0611755 1.073579

|

restricted |

Restricted | -.0025654 .4776976 -0.01 0.996 -.9388355 .9337046

|

time#restricted |

0#Restricted | -.2975714 .3836108 -0.78 0.438 -1.049435 .4542919

3#Restricted | -.840967 .3857439 -2.18 0.029 -1.597011 -.0849228

6#Restricted | -.9763935 .3942163 -2.48 0.013 -1.749043 -.2037436

|

_cons | 4.662381 .3532172 13.20 0.000 3.970088 5.354674

----------------+----------------------------------------------------------------

/sigma_u | 1.83132 .1584757 11.56 0.000 1.520714 2.141927

/sigma_e | 1.188806 .058258 20.41 0.000 1.074623 1.30299

----------------+----------------------------------------------------------------

rho | .7035323 .0424293 .6156657 .780847

---------------------------------------------------------------------------------

LR test of sigma_u=0: chibar2(01) = 180.46 Prob >= chibar2 = 0.000

. testparm time#restricted

( 1) [ln_Hyaluronan]0.time#1.restricted = 0

( 2) [ln_Hyaluronan]3.time#1.restricted = 0

( 3) [ln_Hyaluronan]6.time#1.restricted = 0

chi2( 3) = 8.28

Prob > chi2 = 0.0406

. xttobit ln_Hyaluronan i.time##restricted if per_protocol==1, ll(ln(0.370001)) nolog ///

> vce(bootstrap, reps(500) seed(010967))

(running xttobit on estimation sample)

Random-effects tobit regression Number of obs = 320

Uncensored = 301

Limits: Lower = ln(0.370001) Left-censored = 19

Upper = +inf Right-censored = 0

Replications = 500

Group variable: RecordID Number of groups = 89

Random effects u_i ~ Gaussian Obs per group:

min = 1

avg = 3.6

max = 4

Integration method: mvaghermite Integration pts. = 12

Wald chi2(7) = 24.82

Log likelihood = -601.75091 Prob > chi2 = 0.0008

(Replications based on 89 clusters in RecordID)

---------------------------------------------------------------------------------

| Observed Bootstrap Normal-based

ln_Hyaluronan | coefficient std. err. z P>|z| [95% conf. interval]

----------------+----------------------------------------------------------------

time |

3 | -.164179 .2057182 -0.80 0.425 -.5673793 .2390213

6 | -.3243463 .2242033 -1.45 0.148 -.7637767 .1150842

24 | -.8305479 .3084323 -2.69 0.007 -1.435064 -.2260317

|

restricted |

Restricted | -.3001369 .4481533 -0.67 0.503 -1.178501 .5782274

|

time#restricted |

3#Restricted | -.5433956 .4124978 -1.32 0.188 -1.351876 .2650852

6#Restricted | -.678822 .4063258 -1.67 0.095 -1.475206 .117562

24#Restricted | .2975714 .4537773 0.66 0.512 -.5918158 1.186959

|

_cons | 5.492929 .2801029 19.61 0.000 4.943938 6.041921

----------------+----------------------------------------------------------------

/sigma_u | 1.83132 .1999279 9.16 0.000 1.439469 2.223172

/sigma_e | 1.188806 .1279996 9.29 0.000 .9379316 1.439681

----------------+----------------------------------------------------------------

rho | .7035323 .0466133 .6065753 .7877881

---------------------------------------------------------------------------------

LR test of sigma_u=0: chibar2(01) = 180.46 Prob >= chibar2 = 0.000

. testparm time#restricted

( 1) [ln_Hyaluronan]3.time#1.restricted = 0

( 2) [ln_Hyaluronan]6.time#1.restricted = 0

( 3) [ln_Hyaluronan]24.time#1.restricted = 0

chi2( 3) = 7.66

Prob > chi2 = 0.0536

. xttobit ln_Hyaluronan b24.time##restricted if pscore~=. & per_protocol==1, ll(ln(0.370001)) nolog ///

> vce(bootstrap, reps(500) seed(010967))

(running xttobit on estimation sample)

Random-effects tobit regression Number of obs = 304

Uncensored = 289

Limits: Lower = ln(0.370001) Left-censored = 15

Upper = +inf Right-censored = 0

Replications = 500

Group variable: RecordID Number of groups = 84

Random effects u_i ~ Gaussian Obs per group:

min = 1

avg = 3.6

max = 4

Integration method: mvaghermite Integration pts. = 12

Wald chi2(7) = 24.08

Log likelihood = -564.03478 Prob > chi2 = 0.0011

(Replications based on 84 clusters in RecordID)

---------------------------------------------------------------------------------

| Observed Bootstrap Normal-based

ln_Hyaluronan | coefficient std. err. z P>|z| [95% conf. interval]

----------------+----------------------------------------------------------------

time |

0 | .7914162 .3015657 2.62 0.009 .2003583 1.382474

3 | .6219378 .2296305 2.71 0.007 .1718702 1.072005

6 | .4647332 .2415686 1.92 0.054 -.0087326 .9381989

|

restricted |

Restricted | -.1944691 .5532334 -0.35 0.725 -1.278787 .8898485

|

time#restricted |

0#Restricted | -.1030808 .4332761 -0.24 0.812 -.9522863 .7461247

3#Restricted | -.6904576 .3971597 -1.74 0.082 -1.468876 .0879612

6#Restricted | -.8116832 .3661229 -2.22 0.027 -1.529271 -.0940955

|

_cons | 4.822859 .3848844 12.53 0.000 4.068499 5.577218

----------------+----------------------------------------------------------------

/sigma_u | 1.710263 .1799823 9.50 0.000 1.357505 2.063022

/sigma_e | 1.162775 .1302817 8.93 0.000 .9074277 1.418123

----------------+----------------------------------------------------------------

rho | .6838833 .0492816 .5820311 .7733964

---------------------------------------------------------------------------------

LR test of sigma_u=0: chibar2(01) = 167.97 Prob >= chibar2 = 0.000

. testparm time#restricted

( 1) [ln_Hyaluronan]0.time#1.restricted = 0

( 2) [ln_Hyaluronan]3.time#1.restricted = 0

( 3) [ln_Hyaluronan]6.time#1.restricted = 0

chi2( 3) = 5.47

Prob > chi2 = 0.1406

. xttobit ln_Hyaluronan b24.time##restricted pscore if per_protocol==1, ll(ln(0.370001)) nolog ///

> vce(bootstrap, reps(500) seed(010967))

(running xttobit on estimation sample)

Random-effects tobit regression Number of obs = 304

Uncensored = 289

Limits: Lower = ln(0.370001) Left-censored = 15

Upper = +inf Right-censored = 0

Replications = 500

Group variable: RecordID Number of groups = 84

Random effects u_i ~ Gaussian Obs per group:

min = 1

avg = 3.6

max = 4

Integration method: mvaghermite Integration pts. = 12

Wald chi2(8) = 25.63

Log likelihood = -562.92292 Prob > chi2 = 0.0012

(Replications based on 84 clusters in RecordID)

---------------------------------------------------------------------------------

| Observed Bootstrap Normal-based

ln_Hyaluronan | coefficient std. err. z P>|z| [95% conf. interval]

----------------+----------------------------------------------------------------

time |

0 | .7921649 .301242 2.63 0.009 .2017415 1.382588

3 | .625584 .2296724 2.72 0.006 .1754345 1.075734

6 | .4706461 .2416818 1.95 0.051 -.0030416 .9443338

|

restricted |

Restricted | -.3087485 .5416146 -0.57 0.569 -1.370294 .7527967

|

time#restricted |

0#Restricted | -.1043924 .4331458 -0.24 0.810 -.9533425 .7445578

3#Restricted | -.6910635 .3963277 -1.74 0.081 -1.467851 .0857245

6#Restricted | -.8193898 .3652507 -2.24 0.025 -1.535268 -.1035116

|

pscore | 3.10754 2.298882 1.35 0.176 -1.398186 7.613266

_cons | 3.219474 1.265949 2.54 0.011 .7382608 5.700688

----------------+----------------------------------------------------------------

/sigma_u | 1.684693 .1762925 9.56 0.000 1.339166 2.03022

/sigma_e | 1.16242 .1300747 8.94 0.000 .9074781 1.417361

----------------+----------------------------------------------------------------

rho | .6774677 .0502523 .573822 .7688745

---------------------------------------------------------------------------------

LR test of sigma_u=0: chibar2(01) = 164.81 Prob >= chibar2 = 0.000

. testparm time#restricted

( 1) [ln_Hyaluronan]0.time#1.restricted = 0

( 2) [ln_Hyaluronan]3.time#1.restricted = 0

( 3) [ln_Hyaluronan]6.time#1.restricted = 0

chi2( 3) = 5.63

Prob > chi2 = 0.1313

. xttobit ln_Hyaluronan i.time i.restricted if per_protocol==1 , ll(ln(0.370001)) nolog ///

> vce(bootstrap, reps(500) seed(010967))

(running xttobit on estimation sample)

Random-effects tobit regression Number of obs = 320

Uncensored = 301

Limits: Lower = ln(0.370001) Left-censored = 19

Upper = +inf Right-censored = 0

Replications = 500

Group variable: RecordID Number of groups = 89

Random effects u_i ~ Gaussian Obs per group:

min = 1

avg = 3.6

max = 4

Integration method: mvaghermite Integration pts. = 12

Wald chi2(4) = 14.91

Log likelihood = -605.83176 Prob > chi2 = 0.0049

(Replications based on 89 clusters in RecordID)

-------------------------------------------------------------------------------

| Observed Bootstrap Normal-based

ln_Hyaluronan | coefficient std. err. z P>|z| [95% conf. interval]

--------------+----------------------------------------------------------------

time |

3 | -.4458368 .2030512 -2.20 0.028 -.8438099 -.0478638

6 | -.6801132 .2053396 -3.31 0.001 -1.082571 -.2776549

24 | -.6587544 .2249601 -2.93 0.003 -1.099668 -.2178408

|

restricted |

Restricted | -.5218206 .4191894 -1.24 0.213 -1.343417 .2997755

_cons | 5.608002 .2912874 19.25 0.000 5.037089 6.178915

--------------+----------------------------------------------------------------

/sigma_u | 1.834726 .2032664 9.03 0.000 1.436332 2.233121

/sigma_e | 1.209017 .1323091 9.14 0.000 .9496962 1.468338

--------------+----------------------------------------------------------------

rho | .697237 .0482863 .596926 .7845177

-------------------------------------------------------------------------------

LR test of sigma_u=0: chibar2(01) = 175.52 Prob >= chibar2 = 0.000

. xttobit ln_Hyaluronan b24.time i.restricted if per_protocol==1, ll(ln(0.370001)) nolog ///

> vce(bootstrap, reps(500) seed(010967))

(running xttobit on estimation sample)

Random-effects tobit regression Number of obs = 320

Uncensored = 301

Limits: Lower = ln(0.370001) Left-censored = 19

Upper = +inf Right-censored = 0

Replications = 500

Group variable: RecordID Number of groups = 89

Random effects u_i ~ Gaussian Obs per group:

min = 1

avg = 3.6

max = 4

Integration method: mvaghermite Integration pts. = 12

Wald chi2(4) = 14.91

Log likelihood = -605.83176 Prob > chi2 = 0.0049

(Replications based on 89 clusters in RecordID)

-------------------------------------------------------------------------------

| Observed Bootstrap Normal-based

ln_Hyaluronan | coefficient std. err. z P>|z| [95% conf. interval]

--------------+----------------------------------------------------------------

time |

0 | .6587544 .2249601 2.93 0.003 .2178408 1.099668

3 | .2129176 .208604 1.02 0.307 -.1959388 .621774

6 | -.0213588 .1863949 -0.11 0.909 -.386686 .3439684

|

restricted |

Restricted | -.5218206 .4191894 -1.24 0.213 -1.343417 .2997755

_cons | 4.949248 .3299174 15.00 0.000 4.302621 5.595874

--------------+----------------------------------------------------------------

/sigma_u | 1.834726 .2032664 9.03 0.000 1.436332 2.233121

/sigma_e | 1.209017 .1323091 9.14 0.000 .9496962 1.468338

--------------+----------------------------------------------------------------

rho | .697237 .0482863 .596926 .7845177

-------------------------------------------------------------------------------

LR test of sigma_u=0: chibar2(01) = 175.52 Prob >= chibar2 = 0.000

. xttobit ln_Hyaluronan i.time i.restricted pscore if per_protocol==1, ll(ln(0.370001)) nolog ///

> vce(bootstrap, reps(500) seed(010967))

(running xttobit on estimation sample)

Random-effects tobit regression Number of obs = 304

Uncensored = 289

Limits: Lower = ln(0.370001) Left-censored = 15

Upper = +inf Right-censored = 0

Replications = 500

Group variable: RecordID Number of groups = 84

Random effects u_i ~ Gaussian Obs per group:

min = 1

avg = 3.6

max = 4

Integration method: mvaghermite Integration pts. = 12

Wald chi2(5) = 19.86

Log likelihood = -566.23893 Prob > chi2 = 0.0013

(Replications based on 84 clusters in RecordID)

-------------------------------------------------------------------------------

| Observed Bootstrap Normal-based

ln_Hyaluronan | coefficient std. err. z P>|z| [95% conf. interval]

--------------+----------------------------------------------------------------

time |

3 | -.4648488 .2332283 -1.99 0.046 -.9219679 -.0077298

6 | -.6931805 .2306862 -3.00 0.003 -1.145317 -.2410438

24 | -.7262514 .2083941 -3.48 0.000 -1.134696 -.3178065

|

restricted |

Restricted | -.7045471 .4041517 -1.74 0.081 -1.49667 .0875756

pscore | 3.05173 2.301399 1.33 0.185 -1.458929 7.56239

_cons | 4.19193 1.2287 3.41 0.001 1.783722 6.600138

--------------+----------------------------------------------------------------

/sigma_u | 1.682698 .1761457 9.55 0.000 1.337458 2.027937

/sigma_e | 1.180521 .1352716 8.73 0.000 .9153938 1.445649

--------------+----------------------------------------------------------------

rho | .6701546 .0524464 .5621587 .7655395

-------------------------------------------------------------------------------

LR test of sigma_u=0: chibar2(01) = 160.39 Prob >= chibar2 = 0.000

# IL6 (censored values)

. bysort restricted:tabstat Il6Tn ln_Il6 if per_protocol==1, ///

> s(n mean sd median min max p25 p75) by(time) col(stats) long f(%8.6g)

-> restricted = Standard

time Variable | N Mean SD p50 Min Max p25 p75

------------------+-------------------------------------------------------------------------

0 Il6Tn | 39 66265.4 173159 2519.94 1.6 732164 298.44 26782.4

ln_Il6 | 39 7.98775 2.93982 7.83199 .470004 13.5038 5.69857 10.1955

------------------+-------------------------------------------------------------------------

3 Il6Tn | 38 17856.6 40068.4 1783.93 13.48 162894 283.05 15411.8

ln_Il6 | 38 7.53997 2.37867 7.48641 2.60121 12.0009 5.64562 9.64289

------------------+-------------------------------------------------------------------------

6 Il6Tn | 35 5478.93 10994.2 925.435 10.09 53336 148.08 4872.9

ln_Il6 | 35 6.80796 2.16818 6.83026 2.31154 10.8844 4.99775 8.49144

------------------+-------------------------------------------------------------------------

24 Il6Tn | 35 733.59 1615.94 149.17 1.6 7135.35 49.95 448.22

ln_Il6 | 35 4.91992 1.96731 5.00509 .470004 8.87282 3.91102 6.10528

------------------+-------------------------------------------------------------------------

Total Il6Tn |147 23675.8 94544.2 786.76 1.6 732164 148.08 5670.9

ln_Il6 |147 6.86066 2.65376 6.66792 .470004 13.5038 4.99775 8.6431

--------------------------------------------------------------------------------------------

-> restricted = Restricted

time Variable | N Mean SD p50 Min Max p25 p75

------------------+--------------------------------------------------------------------------

0 Il6Tn | 45 50126.4 148346 905.89 15.57 739643 291.48 11479.3

ln_Il6 | 45 7.47585 2.64403 6.80892 2.74535 13.5139 5.67497 9.3483

------------------+--------------------------------------------------------------------------

3 Il6Tn | 41 13831.2 46094.2 282.42 1.6 207970 89.26 1025.56

ln_Il6 | 41 6.0799 2.49108 5.6434 .470004 12.2451 4.49155 6.93299

------------------+--------------------------------------------------------------------------

6 Il6Tn | 41 12485.5 41938.8 313.75 21.97 235183 106.44 1662.65

ln_Il6 | 41 6.29634 2.32415 5.7486 3.08968 12.3681 4.66758 7.41617

------------------+--------------------------------------------------------------------------

24 Il6Tn | 44 555.417 1137 85.305 1.6 5520.32 15.88 358.825

ln_Il6 | 44 4.55068 2.10698 4.44344 .470004 8.61619 2.76257 5.86911

------------------+--------------------------------------------------------------------------

Total Il6Tn | 171 19643.9 83490.6 299.78 1.6 739643 85.02 1814.67

ln_Il6 | 171 6.10567 2.60674 5.70305 .470004 13.5139 4.44289 7.50366

---------------------------------------------------------------------------------------------

. xttobit ln_Il6 i.time##restricted if per_protocol==1, ll(ln(1.60001)) nolog

Random-effects tobit regression Number of obs = 318

Uncensored = 311

Limits: Lower = ln(1.60001) Left-censored = 7

Upper = +inf Right-censored = 0

Group variable: RecordID Number of groups = 89

Random effects u_i ~ Gaussian Obs per group:

min = 1

avg = 3.6

max = 4

Integration method: mvaghermite Integration pts. = 12

Wald chi2(7) = 228.02

Log likelihood = -641.9077 Prob > chi2 = 0.0000

---------------------------------------------------------------------------------

ln_Il6 | Coefficient Std. err. z P>|z| [95% conf. interval]

----------------+----------------------------------------------------------------

time |

3 | -.4959235 .3145478 -1.58 0.115 -1.112426 .1205788

6 | -1.179262 .3236714 -3.64 0.000 -1.813646 -.5448772

24 | -3.31638 .327186 -10.14 0.000 -3.957652 -2.675107

|

restricted |

Restricted | -.465789 .5216056 -0.89 0.372 -1.488117 .5565391

|

time#restricted |

3#Restricted | -.7933655 .4349548 -1.82 0.068 -1.645861 .0591302

6#Restricted | .0398196 .4404505 0.09 0.928 -.8234476 .9030868

24#Restricted | .2632742 .4399342 0.60 0.550 -.598981 1.125529

|

_cons | 7.931596 .382489 20.74 0.000 7.181932 8.681261

----------------+----------------------------------------------------------------

/sigma_u | 1.995991 .1717716 11.62 0.000 1.659325 2.332657

/sigma_e | 1.366663 .0651132 20.99 0.000 1.239043 1.494283

----------------+----------------------------------------------------------------

rho | .6808188 .0440519 .5902914 .7616701

---------------------------------------------------------------------------------

LR test of sigma_u=0: chibar2(01) = 168.31 Prob >= chibar2 = 0.000

. testparm time#restricted

( 1) [ln_Il6]3.time#1.restricted = 0

( 2) [ln_Il6]6.time#1.restricted = 0

( 3) [ln_Il6]24.time#1.restricted = 0

chi2( 3) = 6.50

Prob > chi2 = 0.0895

. xttobit ln_Il6 i.time##restricted if per_protocol==1, ll(ln(1.60001)) nolog ///

> vce(bootstrap, reps(500) seed(010967))

(running xttobit on estimation sample)

Random-effects tobit regression Number of obs = 318

Uncensored = 311

Limits: Lower = ln(1.60001) Left-censored = 7

Upper = +inf Right-censored = 0

Replications = 500

Group variable: RecordID Number of groups = 89

Random effects u_i ~ Gaussian Obs per group:

min = 1

avg = 3.6

max = 4

Integration method: mvaghermite Integration pts. = 12

Wald chi2(7) = 157.27

Log likelihood = -641.9077 Prob > chi2 = 0.0000

(Replications based on 89 clusters in RecordID)

---------------------------------------------------------------------------------

| Observed Bootstrap Normal-based

ln_Il6 | coefficient std. err. z P>|z| [95% conf. interval]

----------------+----------------------------------------------------------------

time |

3 | -.4959235 .2456755 -2.02 0.044 -.9774387 -.0144083

6 | -1.179262 .2703207 -4.36 0.000 -1.70908 -.6494427

24 | -3.31638 .3713414 -8.93 0.000 -4.044195 -2.588564

|

restricted |

Restricted | -.465789 .6137934 -0.76 0.448 -1.668802 .737224

|

time#restricted |

3#Restricted | -.7933655 .3982539 -1.99 0.046 -1.573929 -.0128022

6#Restricted | .0398196 .3428296 0.12 0.908 -.632114 .7117532

24#Restricted | .2632742 .5316839 0.50 0.620 -.7788071 1.305356

|

_cons | 7.931596 .4435061 17.88 0.000 7.06234 8.800852

----------------+----------------------------------------------------------------

/sigma_u | 1.995991 .1624054 12.29 0.000 1.677682 2.3143

/sigma_e | 1.366663 .1302237 10.49 0.000 1.111429 1.621897

----------------+----------------------------------------------------------------

rho | .6808188 .0519214 .5734315 .7748342

---------------------------------------------------------------------------------

LR test of sigma_u=0: chibar2(01) = 168.31 Prob >= chibar2 = 0.000

. testparm time#restricted

( 1) [ln_Il6]3.time#1.restricted = 0

( 2) [ln_Il6]6.time#1.restricted = 0

( 3) [ln_Il6]24.time#1.restricted = 0

chi2( 3) = 8.98

Prob > chi2 = 0.0296

. xttobit ln_Il6 i.time##restricted if pscore~=. & per_protocol==1, ll(ln(1.60001)) nolog ////

> vce(bootstrap, reps(500) seed(010967))

(running xttobit on estimation sample)

Random-effects tobit regression Number of obs = 302

Uncensored = 295

Limits: Lower = ln(1.60001) Left-censored = 7

Upper = +inf Right-censored = 0

Replications = 500

Group variable: RecordID Number of groups = 84

Random effects u_i ~ Gaussian Obs per group:

min = 1

avg = 3.6

max = 4

Integration method: mvaghermite Integration pts. = 12

Wald chi2(7) = 146.14

Log likelihood = -614.4619 Prob > chi2 = 0.0000

(Replications based on 84 clusters in RecordID)

---------------------------------------------------------------------------------

| Observed Bootstrap Normal-based

ln_Il6 | coefficient std. err. z P>|z| [95% conf. interval]

----------------+----------------------------------------------------------------

time |

3 | -.4362846 .2561595 -1.70 0.089 -.9383479 .0657788

6 | -1.117504 .268677 -4.16 0.000 -1.644101 -.5909066

24 | -3.265849 .3710349 -8.80 0.000 -3.993064 -2.538634

|

restricted |

Restricted | -.3800654 .598966 -0.63 0.526 -1.554017 .7938863

|

time#restricted |

3#Restricted | -.8861925 .4207093 -2.11 0.035 -1.710768 -.0616174

6#Restricted | -.0231639 .3438572 -0.07 0.946 -.6971117 .6507839

24#Restricted | .2802223 .5379316 0.52 0.602 -.7741043 1.334549

|

_cons | 7.883901 .4558949 17.29 0.000 6.990363 8.777438

----------------+----------------------------------------------------------------

/sigma_u | 2.046322 .1609637 12.71 0.000 1.730839 2.361805

/sigma_e | 1.387856 .1393197 9.96 0.000 1.114794 1.660917

----------------+----------------------------------------------------------------

rho | .6849399 .0562621 .5679535 .7858049

---------------------------------------------------------------------------------

LR test of sigma_u=0: chibar2(01) = 161.85 Prob >= chibar2 = 0.000

. testparm time#restricted

( 1) [ln_Il6]3.time#1.restricted = 0

( 2) [ln_Il6]6.time#1.restricted = 0

( 3) [ln_Il6]24.time#1.restricted = 0

chi2( 3) = 9.17

Prob > chi2 = 0.0271

. xttobit ln_Il6 i.time##restricted pscore if per_protocol==1, ll(ln(1.60001)) nolog ///

> vce(bootstrap, reps(500) seed(010967))

(running xttobit on estimation sample)

Random-effects tobit regression Number of obs = 302

Uncensored = 295

Limits: Lower = ln(1.60001) Left-censored = 7

Upper = +inf Right-censored = 0

Replications = 500

Group variable: RecordID Number of groups = 84

Random effects u_i ~ Gaussian Obs per group:

min = 1

avg = 3.6

max = 4

Integration method: mvaghermite Integration pts. = 12

Wald chi2(8) = 146.72

Log likelihood = -612.93766 Prob > chi2 = 0.0000

(Replications based on 84 clusters in RecordID)

---------------------------------------------------------------------------------

| Observed Bootstrap Normal-based

ln_Il6 | coefficient std. err. z P>|z| [95% conf. interval]

----------------+----------------------------------------------------------------

time |

3 | -.4317967 .2567436 -1.68 0.093 -.9350049 .0714115

6 | -1.109392 .2688742 -4.13 0.000 -1.636375 -.5824078

24 | -3.264794 .3713785 -8.79 0.000 -3.992683 -2.536906

|

restricted |

Restricted | -.5416345 .5927078 -0.91 0.361 -1.70332 .6200516

|

time#restricted |

3#Restricted | -.8838692 .4199808 -2.10 0.035 -1.707016 -.060722

6#Restricted | -.0349008 .3432004 -0.10 0.919 -.7075613 .6377597

24#Restricted | .2791145 .5384835 0.52 0.604 -.7762936 1.334523

|

pscore | 4.342526 2.410045 1.80 0.072 -.3810765 9.066128

_cons | 5.644501 1.363006 4.14 0.000 2.973059 8.315944

----------------+----------------------------------------------------------------

/sigma_u | 2.004458 .1573813 12.74 0.000 1.695996 2.312919

/sigma_e | 1.387764 .13923 9.97 0.000 1.114878 1.66065

----------------+----------------------------------------------------------------

rho | .6759802 .0578134 .5561701 .7798566

---------------------------------------------------------------------------------

LR test of sigma_u=0: chibar2(01) = 156.88 Prob >= chibar2 = 0.000

. testparm time#restricted

( 1) [ln_Il6]3.time#1.restricted = 0

( 2) [ln_Il6]6.time#1.restricted = 0

( 3) [ln_Il6]24.time#1.restricted = 0

chi2( 3) = 8.98

Prob > chi2 = 0.0296

# IL10 (censored values)

. bysort restricted:tabstat Il10Tn ln_Il10 if per_protocol==1, ///

> s(n mean sd median min max p25 p75) by(time) col(stats) long f(%6.0g)

-> restricted = Standard

time Variable | N Mean SD p50 Min Max p25 p75

----------------+-------------------------------------------------------------------------

0 Il10Tn | 37 284.7 1476 .13 .13 9011 .13 55

ln_Il10 | 37 .9598 3.366 -2.04 -2.04 9.106 -2.04 4.007

----------------+-------------------------------------------------------------------------

3 Il10Tn | 38 173.7 628.1 10.84 .13 3494 .13 49.89

ln_Il10 | 38 1.533 3.203 2.382 -2.04 8.159 -2.04 3.91

----------------+-------------------------------------------------------------------------

6 Il10Tn | 34 41.78 66.9 13.66 .13 248 .13 39.13

ln_Il10 | 34 1.697 2.79 2.614 -2.04 5.513 -2.04 3.667

----------------+-------------------------------------------------------------------------

24 Il10Tn | 34 3.315 8.987 .13 .13 44.35 .13 .13

ln_Il10 | 34 -1.089 1.843 -2.04 -2.04 3.792 -2.04 -2.04

----------------+-------------------------------------------------------------------------

Total Il10Tn |143 130.6 817.9 .71 .13 9011 .13 34.09

ln_Il10 |143 .8002 3.056 -.3425 -2.04 9.106 -2.04 3.529

------------------------------------------------------------------------------------------

-> restricted = Restricted

time Variable | N Mean SD p50 Min Max p25 p75

-----------------+--------------------------------------------------------------------------

0 Il10Tn | 44 212.9 723.1 .13 .13 4294 .13 57.08

ln_Il10 | 44 1.122 3.531 -2.04 -2.04 8.365 -2.04 4.041

-----------------+--------------------------------------------------------------------------

3 Il10Tn | 40 120.5 494 .13 .13 3101 .13 35.07

ln_Il10 | 40 .4348 3.266 -2.04 -2.04 8.039 -2.04 3.553

-----------------+--------------------------------------------------------------------------

6 Il10Tn | 38 87.73 227.7 7.27 .13 1309 .13 50.74

ln_Il10 | 38 1.662 3.027 1.98 -2.04 7.177 -2.04 3.927

-----------------+--------------------------------------------------------------------------

24 Il10Tn | 43 10.8 36.9 .13 .13 195.3 .13 .13

ln_Il10 | 43 -.842 2.263 -2.04 -2.04 5.275 -2.04 -2.04

-----------------+--------------------------------------------------------------------------

Total Il10Tn | 165 109 461.2 .13 .13 4294 .13 31.71

ln_Il10 | 165 .5679 3.173 -2.04 -2.04 8.365 -2.04 3.457

--------------------------------------------------------------------------------------------

. xttobit ln_Il10 i.time##restricted if per_protocol==1, ll(ln(0.130001)) nolog

Random-effects tobit regression Number of obs = 308

Uncensored = 144

Limits: Lower = ln(0.130001) Left-censored = 164

Upper = +inf Right-censored = 0

Group variable: RecordID Number of groups = 89

Random effects u_i ~ Gaussian Obs per group:

min = 1

avg = 3.5

max = 4

Integration method: mvaghermite Integration pts. = 12

Wald chi2(7) = 60.58

Log likelihood = -531.63944 Prob > chi2 = 0.0000

---------------------------------------------------------------------------------

ln_Il10 | Coefficient Std. err. z P>|z| [95% conf. interval]

----------------+----------------------------------------------------------------

time |

3 | 1.024617 .9781135 1.05 0.295 -.8924504 2.941684

6 | 1.626181 1.008188 1.61 0.107 -.3498313 3.602193

24 | -4.412731 1.147661 -3.84 0.000 -6.662104 -2.163357

|

restricted |

Restricted | -.0333262 1.297852 -0.03 0.980 -2.57707 2.510418

|

time#restricted |

3#Restricted | -2.249183 1.397542 -1.61 0.108 -4.988316 .4899491

6#Restricted | -.2214214 1.384181 -0.16 0.873 -2.934367 2.491524

24#Restricted | -.1085958 1.557368 -0.07 0.944 -3.16098 2.943788

|

_cons | -.839648 .9616696 -0.87 0.383 -2.724486 1.04519

----------------+----------------------------------------------------------------

/sigma_u | 3.965136 .4968396 7.98 0.000 2.991348 4.938924

/sigma_e | 3.721949 .2866596 12.98 0.000 3.160107 4.283792

----------------+----------------------------------------------------------------

rho | .5316041 .0717642 .391897 .6674867

---------------------------------------------------------------------------------

LR test of sigma_u=0: chibar2(01) = 53.36 Prob >= chibar2 = 0.000

. testparm time#restricted

( 1) [ln_Il10]3.time#1.restricted = 0

( 2) [ln_Il10]6.time#1.restricted = 0

( 3) [ln_Il10]24.time#1.restricted = 0

chi2( 3) = 3.40

Prob > chi2 = 0.3336

. xttobit ln_Il10 i.time##restricted if per_protocol==1, ll(ln(0.130001)) nolog ///

> vce(bootstrap, reps(500) seed(010967))

(running xttobit on estimation sample)

Random-effects tobit regression Number of obs = 308

Uncensored = 144

Limits: Lower = ln(0.130001) Left-censored = 164

Upper = +inf Right-censored = 0

Replications = 500

Group variable: RecordID Number of groups = 89

Random effects u_i ~ Gaussian Obs per group:

min = 1

avg = 3.5

max = 4

Integration method: mvaghermite Integration pts. = 12

Wald chi2(7) = 66.21

Log likelihood = -531.63944 Prob > chi2 = 0.0000

(Replications based on 89 clusters in RecordID)

---------------------------------------------------------------------------------

| Observed Bootstrap Normal-based

ln_Il10 | coefficient std. err. z P>|z| [95% conf. interval]

----------------+----------------------------------------------------------------

time |

3 | 1.024617 .8787427 1.17 0.244 -.6976873 2.746921

6 | 1.626181 1.027316 1.58 0.113 -.3873213 3.639683

24 | -4.412731 1.480958 -2.98 0.003 -7.315355 -1.510106

|

restricted |

Restricted | -.0333262 1.349314 -0.02 0.980 -2.677934 2.611282

|

time#restricted |

3#Restricted | -2.249183 1.253168 -1.79 0.073 -4.705347 .2069801

6#Restricted | -.2214214 1.307853 -0.17 0.866 -2.784765 2.341922

24#Restricted | -.1085958 1.973399 -0.06 0.956 -3.976386 3.759194

|

_cons | -.839648 1.030589 -0.81 0.415 -2.859566 1.18027

----------------+----------------------------------------------------------------

/sigma_u | 3.965136 .4404333 9.00 0.000 3.101902 4.828369

/sigma_e | 3.721949 .2703381 13.77 0.000 3.192096 4.251802

----------------+----------------------------------------------------------------

rho | .5316041 .0723431 .3908013 .6685224

---------------------------------------------------------------------------------

LR test of sigma_u=0: chibar2(01) = 53.36 Prob >= chibar2 = 0.000

. testparm time#restricted

( 1) [ln_Il10]3.time#1.restricted = 0

( 2) [ln_Il10]6.time#1.restricted = 0

( 3) [ln_Il10]24.time#1.restricted = 0

chi2( 3) = 4.64

Prob > chi2 = 0.2005

. xttobit ln_Il10 i.time##restricted if pscore~=. & per_protocol==1, ll(ln(0.130001)) nolog ///

> vce(bootstrap, reps(500) seed(010967))

(running xttobit on estimation sample)

Random-effects tobit regression Number of obs = 292

Uncensored = 140

Limits: Lower = ln(0.130001) Left-censored = 152

Upper = +inf Right-censored = 0

Replications = 500

Group variable: RecordID Number of groups = 84

Random effects u_i ~ Gaussian Obs per group:

min = 1

avg = 3.5

max = 4

Integration method: mvaghermite Integration pts. = 12

Wald chi2(7) = 63.83

Log likelihood = -513.19056 Prob > chi2 = 0.0000

(Replications based on 84 clusters in RecordID)

---------------------------------------------------------------------------------

| Observed Bootstrap Normal-based

ln_Il10 | coefficient std. err. z P>|z| [95% conf. interval]

----------------+----------------------------------------------------------------

time |

3 | 1.024696 .8744652 1.17 0.241 -.6892249 2.738616

6 | 1.302738 1.028853 1.27 0.205 -.7137768 3.319253

24 | -4.383879 1.537119 -2.85 0.004 -7.396576 -1.371182

|

restricted |

Restricted | -.019842 1.318493 -0.02 0.988 -2.604041 2.564357

|

time#restricted |

3#Restricted | -2.330195 1.321697 -1.76 0.078 -4.920674 .2602834

6#Restricted | -.0464456 1.373499 -0.03 0.973 -2.738454 2.645563

24#Restricted | -.0277931 1.983982 -0.01 0.989 -3.916326 3.86074

|

_cons | -.6321121 1.016937 -0.62 0.534 -2.625272 1.361048

----------------+----------------------------------------------------------------

/sigma_u | 3.94729 .4455931 8.86 0.000 3.073943 4.820636

/sigma_e | 3.67808 .2548149 14.43 0.000 3.178652 4.177508

----------------+----------------------------------------------------------------

rho | .5352606 .0710059 .3967713 .6695657

---------------------------------------------------------------------------------

LR test of sigma_u=0: chibar2(01) = 51.73 Prob >= chibar2 = 0.000

. testparm time#restricted

( 1) [ln_Il10]3.time#1.restricted = 0

( 2) [ln_Il10]6.time#1.restricted = 0

( 3) [ln_Il10]24.time#1.restricted = 0

chi2( 3) = 5.66

Prob > chi2 = 0.1293

. xttobit ln_Il10 i.time##restricted pscore if per_protocol==1, ll(ln(0.130001)) nolog ///

> vce(bootstrap, reps(500) seed(010967))

(running xttobit on estimation sample)

Random-effects tobit regression Number of obs = 292

Uncensored = 140

Limits: Lower = ln(0.130001) Left-censored = 152

Upper = +inf Right-censored = 0

Replications = 500

Group variable: RecordID Number of groups = 84

Random effects u_i ~ Gaussian Obs per group:

min = 1

avg = 3.5

max = 4

Integration method: mvaghermite Integration pts. = 12

Wald chi2(8) = 64.45

Log likelihood = -512.05027 Prob > chi2 = 0.0000

(Replications based on 84 clusters in RecordID)

---------------------------------------------------------------------------------

| Observed Bootstrap Normal-based

ln_Il10 | coefficient std. err. z P>|z| [95% conf. interval]

----------------+----------------------------------------------------------------

time |

3 | 1.043856 .8708357 1.20 0.231 -.6629504 2.750663

6 | 1.327412 1.020899 1.30 0.194 -.6735138 3.328339

24 | -4.3643 1.533936 -2.85 0.004 -7.370759 -1.357841

|

restricted |

Restricted | -.319416 1.327684 -0.24 0.810 -2.921628 2.282796

|

time#restricted |

3#Restricted | -2.336884 1.319605 -1.77 0.077 -4.923262 .2494936

6#Restricted | -.0907238 1.371976 -0.07 0.947 -2.779747 2.5983

24#Restricted | -.0528914 1.986845 -0.03 0.979 -3.947037 3.841254

|

pscore | 8.086079 6.254627 1.29 0.196 -4.172764 20.34492

_cons | -4.819379 3.697868 -1.30 0.192 -12.06707 2.42831

----------------+----------------------------------------------------------------

/sigma_u | 3.898893 .4323111 9.02 0.000 3.051579 4.746208

/sigma_e | 3.674861 .2546255 14.43 0.000 3.175804 4.173918

----------------+----------------------------------------------------------------

rho | .5295542 .0707196 .3919479 .6636861

---------------------------------------------------------------------------------

LR test of sigma_u=0: chibar2(01) = 50.47 Prob >= chibar2 = 0.000

. testparm time#restricted

( 1) [ln_Il10]3.time#1.restricted = 0

( 2) [ln_Il10]6.time#1.restricted = 0

( 3) [ln_Il10]24.time#1.restricted = 0

chi2( 3) = 5.58

Prob > chi2 = 0.1341

. xttobit ln_Il10 i.time i.restricted if per_protocol==1, ll(ln(0.130001)) nolog ///

> vce(bootstrap, reps(500) seed(010967))

(running xttobit on estimation sample)

Random-effects tobit regression Number of obs = 308

Uncensored = 144

Limits: Lower = ln(0.130001) Left-censored = 164

Upper = +inf Right-censored = 0

Replications = 500

Group variable: RecordID Number of groups = 89

Random effects u_i ~ Gaussian Obs per group:

min = 1

avg = 3.5

max = 4

Integration method: mvaghermite Integration pts. = 12

Wald chi2(4) = 54.08

Log likelihood = -533.34511 Prob > chi2 = 0.0000

(Replications based on 89 clusters in RecordID)

------------------------------------------------------------------------------

| Observed Bootstrap Normal-based

ln_Il10 | coefficient std. err. z P>|z| [95% conf. interval]

-------------+----------------------------------------------------------------

time |

3 | -.0499521 .6486385 -0.08 0.939 -1.32126 1.221356

6 | 1.510951 .6699975 2.26 0.024 .1977805 2.824122

24 | -4.47934 .9782268 -4.58 0.000 -6.39663 -2.562051

|

restricted |

Restricted | -.7084443 1.049554 -0.67 0.500 -2.765533 1.348644

_cons | -.5053812 .866413 -0.58 0.560 -2.20352 1.192757

-------------+----------------------------------------------------------------

/sigma_u | 3.964273 .4441634 8.93 0.000 3.093729 4.834818

/sigma_e | 3.763231 .2699912 13.94 0.000 3.234058 4.292404

-------------+----------------------------------------------------------------

rho | .5259989 .0731235 .3840742 .6646652

------------------------------------------------------------------------------

LR test of sigma_u=0: chibar2(01) = 52.31 Prob >= chibar2 = 0.000

. xttobit ln_Il10 i.time i.restricted pscore if per_protocol==1, ll(ln(0.130001)) nolog ///

> vce(bootstrap, reps(500) seed(010967))

(running xttobit on estimation sample)

Random-effects tobit regression Number of obs = 292

Uncensored = 140

Limits: Lower = ln(0.130001) Left-censored = 152

Upper = +inf Right-censored = 0

Replications = 500

Group variable: RecordID Number of groups = 84

Random effects u_i ~ Gaussian Obs per group:

min = 1

avg = 3.5

max = 4

Integration method: mvaghermite Integration pts. = 12

Wald chi2(5) = 47.71

Log likelihood = -513.9537 Prob > chi2 = 0.0000

(Replications based on 84 clusters in RecordID)

------------------------------------------------------------------------------

| Observed Bootstrap Normal-based

ln_Il10 | coefficient std. err. z P>|z| [95% conf. interval]

-------------+----------------------------------------------------------------

time |

3 | -.0379075 .6472771 -0.06 0.953 -1.306547 1.230732

6 | 1.288561 .6655999 1.94 0.053 -.0159909 2.593113

24 | -4.394971 .982644 -4.47 0.000 -6.320918 -2.469024

|

restricted |

Restricted | -.9740753 .9654203 -1.01 0.313 -2.866264 .9181137

pscore | 8.19262 6.255416 1.31 0.190 -4.06777 20.45301

_cons | -4.562553 3.562702 -1.28 0.200 -11.54532 2.420215

-------------+----------------------------------------------------------------

/sigma_u | 3.89574 .437218 8.91 0.000 3.038808 4.752671

/sigma_e | 3.72296 .2570169 14.49 0.000 3.219217 4.226704

-------------+----------------------------------------------------------------

rho | .5226666 .0715708 .3838665 .6587435

------------------------------------------------------------------------------

LR test of sigma_u=0: chibar2(01) = 49.26 Prob >= chibar2 = 0.000

# VEGFR1 (censored values)

. bysort restricted:tabstat VEGFR1Tn ln_VEGFR1 if per_protocol==1, ///

> s(n mean sd median min max p25 p75) by(time) col(stats) long f(%7.0g)

-> restricted = Standard

time Variable | N Mean SD p50 Min Max p25 p75

-----------------+--------------------------------------------------------------------------

0 VEGFR1Tn | 38 2851 5214.3 512.85 125 24001 125 2555.7

ln_VEGFR1 | 38 6.562 1.7089 6.24 4.8283 10.086 4.8283 7.8461

-----------------+--------------------------------------------------------------------------

3 VEGFR1Tn | 39 2270.1 4468.6 652.5 125 24001 125 2347.5

ln_VEGFR1 | 39 6.431 1.6316 6.4808 4.8283 10.086 4.8283 7.7611

-----------------+--------------------------------------------------------------------------

6 VEGFR1Tn | 35 2484.2 4676.7 372.6 125 24001 125 2993.1

ln_VEGFR1 | 35 6.4392 1.6887 5.9205 4.8283 10.086 4.8283 8.0041

-----------------+--------------------------------------------------------------------------

24 VEGFR1Tn | 32 3442 5206.4 1278.2 125 24001 792.9 3739.5

ln_VEGFR1 | 32 7.2254 1.4484 7.1531 4.8283 10.086 6.6753 8.2254

-----------------+--------------------------------------------------------------------------

Total VEGFR1Tn | 144 2735.9 4858.7 776.1 125 24001 125 2771.1

ln_VEGFR1 | 144 6.6441 1.6419 6.6543 4.8283 10.086 4.8283 7.927

--------------------------------------------------------------------------------------------

-> restricted = Restricted

time Variable | N Mean SD p50 Min Max p25 p75

------------------+-------------------------------------------------------------------------

0 VEGFR1Tn | 45 1991 4039.4 728.1 125 24001 125 1955.7

ln_VEGFR1 | 45 6.3607 1.5806 6.5904 4.8283 10.086 4.8283 7.5785

------------------+-------------------------------------------------------------------------

3 VEGFR1Tn | 42 2279.3 5347.6 385.5 125 24001 125 1399.8

ln_VEGFR1 | 42 6.2693 1.5869 5.9543 4.8283 10.086 4.8283 7.2441

------------------+-------------------------------------------------------------------------

6 VEGFR1Tn | 41 2133.9 4664.2 320.4 125 24001 125 1264.5

ln_VEGFR1 | 41 6.1912 1.608 5.7696 4.8283 10.086 4.8283 7.1424

------------------+-------------------------------------------------------------------------

24 VEGFR1Tn | 41 3189.5 5459.6 986.4 125 24001 333 2944.8

ln_VEGFR1 | 41 6.9699 1.5701 6.8941 4.8283 10.086 5.8081 7.9878

------------------+-------------------------------------------------------------------------

Total VEGFR1Tn |169 2388.1 4873.1 620.7 125 24001 125 1903.5

ln_VEGFR1 |169 6.4446 1.6013 6.4308 4.8283 10.086 4.8283 7.5514

--------------------------------------------------------------------------------------------

. xttobit ln_VEGFR1 i.time##restricted if per_protocol==1, ll(ln(125.0001)) ul(ln(24000)) nolog

Random-effects tobit regression Number of obs = 313

Uncensored = 186

Limits: Lower = ln(125.0001) Left-censored = 117

Upper = ln(24000) Right-censored = 10

Group variable: RecordID Number of groups = 89

Random effects u_i ~ Gaussian Obs per group:

min = 1

avg = 3.5

max = 4

Integration method: mvaghermite Integration pts. = 12

Wald chi2(7) = 33.30

Log likelihood = -441.1832 Prob > chi2 = 0.0000

---------------------------------------------------------------------------------

ln_VEGFR1 | Coefficient Std. err. z P>|z| [95% conf. interval]

----------------+----------------------------------------------------------------

time |

3 | -.2664364 .2689572 -0.99 0.322 -.7935829 .2607101

6 | -.3316354 .2768075 -1.20 0.231 -.8741681 .2108974

24 | .7424095 .2817583 2.63 0.008 .1901734 1.294646

|

restricted |

Restricted | -.3468469 .5325722 -0.65 0.515 -1.390669 .6969755

|

time#restricted |

3#Restricted | .1663491 .3753683 0.44 0.658 -.5693592 .9020573

6#Restricted | .1641641 .3817484 0.43 0.667 -.584049 .9123772

24#Restricted | .0030201 .3801927 0.01 0.994 -.7421439 .748184

|

_cons | 6.133511 .3895755 15.74 0.000 5.369957 6.897065

----------------+----------------------------------------------------------------

/sigma_u | 2.132889 .2084114 10.23 0.000 1.724411 2.541368

/sigma_e | 1.033689 .0683513 15.12 0.000 .8997232 1.167655

----------------+----------------------------------------------------------------

rho | .8097962 .0348545 .7342018 .8704921

---------------------------------------------------------------------------------

LR test of sigma_u=0: chibar2(01) = 206.72 Prob >= chibar2 = 0.000

. testparm time#restricted

( 1) [ln_VEGFR1]3.time#1.restricted = 0

( 2) [ln_VEGFR1]6.time#1.restricted = 0

( 3) [ln_VEGFR1]24.time#1.restricted = 0

chi2( 3) = 0.37

Prob > chi2 = 0.9470

. xttobit ln_VEGFR1 i.time##restricted if pscore~=. & per_protocol==1, ll(ln(125.0001)) ul(ln(24000)) nolog

Random-effects tobit regression Number of obs = 297

Uncensored = 176

Limits: Lower = ln(125.0001) Left-censored = 111

Upper = ln(24000) Right-censored = 10

Group variable: RecordID Number of groups = 84

Random effects u_i ~ Gaussian Obs per group:

min = 1

avg = 3.5

max = 4

Integration method: mvaghermite Integration pts. = 12

Wald chi2(7) = 31.39

Log likelihood = -420.34976 Prob > chi2 = 0.0001

---------------------------------------------------------------------------------

ln_VEGFR1 | Coefficient Std. err. z P>|z| [95% conf. interval]

----------------+----------------------------------------------------------------

time |

3 | -.2834542 .2768327 -1.02 0.306 -.8260364 .259128

6 | -.3133768 .2849054 -1.10 0.271 -.8717811 .2450276

24 | .7692173 .2909075 2.64 0.008 .199049 1.339386

|

restricted |

Restricted | -.2422169 .555032 -0.44 0.663 -1.33006 .8456258

|

time#restricted |

3#Restricted | .1802043 .3884538 0.46 0.643 -.5811512 .9415598

6#Restricted | .0520684 .3937234 0.13 0.895 -.7196153 .8237521

24#Restricted | -.0750278 .3944254 -0.19 0.849 -.8480874 .6980318

|

_cons | 6.107325 .4045798 15.10 0.000 5.314363 6.900287

----------------+----------------------------------------------------------------

/sigma_u | 2.169569 .2178953 9.96 0.000 1.742502 2.596636

/sigma_e | 1.044088 .0710189 14.70 0.000 .9048941 1.183283

----------------+----------------------------------------------------------------

rho | .8119558 .0355094 .7346757 .8735298

---------------------------------------------------------------------------------

LR test of sigma_u=0: chibar2(01) = 197.29 Prob >= chibar2 = 0.000

. testparm time#restricted

( 1) [ln_VEGFR1]3.time#1.restricted = 0

( 2) [ln_VEGFR1]6.time#1.restricted = 0

( 3) [ln_VEGFR1]24.time#1.restricted = 0

chi2( 3) = 0.44

Prob > chi2 = 0.9324

. xttobit ln_VEGFR1 i.time##restricted if per_protocol==1, ll(ln(125.0001)) ul(ln(24000)) nolog ///

> vce(bootstrap, reps(500) seed(010967))

(running xttobit on estimation sample)

Random-effects tobit regression Number of obs = 313

Uncensored = 186

Limits: Lower = ln(125.0001) Left-censored = 117

Upper = ln(24000) Right-censored = 10

Replications = 500

Group variable: RecordID Number of groups = 89

Random effects u_i ~ Gaussian Obs per group:

min = 1

avg = 3.5

max = 4

Integration method: mvaghermite Integration pts. = 12

Wald chi2(7) = 24.06

Log likelihood = -441.1832 Prob > chi2 = 0.0011

(Replications based on 89 clusters in RecordID)

---------------------------------------------------------------------------------

| Observed Bootstrap Normal-based

ln_VEGFR1 | coefficient std. err. z P>|z| [95% conf. interval]

----------------+----------------------------------------------------------------

time |

3 | -.2664364 .2399864 -1.11 0.267 -.7368011 .2039282

6 | -.3316354 .2110579 -1.57 0.116 -.7453012 .0820305

24 | .7424095 .2913058 2.55 0.011 .1714605 1.313358

|

restricted |

Restricted | -.3468469 .5592761 -0.62 0.535 -1.443008 .7493141

|

time#restricted |

3#Restricted | .1663491 .3806015 0.44 0.662 -.5796162 .9123144

6#Restricted | .1641641 .3439693 0.48 0.633 -.5100032 .8383315

24#Restricted | .0030201 .3754752 0.01 0.994 -.7328978 .7389379

|

_cons | 6.133511 .435187 14.09 0.000 5.28056 6.986462

----------------+----------------------------------------------------------------

/sigma_u | 2.132889 .2178465 9.79 0.000 1.705918 2.559861

/sigma_e | 1.033689 .1014612 10.19 0.000 .8348291 1.23255

----------------+----------------------------------------------------------------

rho | .8097962 .0435374 .7132484 .8832501

---------------------------------------------------------------------------------

LR test of sigma_u=0: chibar2(01) = 206.72 Prob >= chibar2 = 0.000

. testparm time#restricted

( 1) [ln_VEGFR1]3.time#1.restricted = 0

( 2) [ln_VEGFR1]6.time#1.restricted = 0

( 3) [ln_VEGFR1]24.time#1.restricted = 0

chi2( 3) = 0.35

Prob > chi2 = 0.9513

. xttobit ln_VEGFR1 i.time##restricted pscore if per_protocol==1, ll(ln(125.0001)) ul(ln(24000)) nolog

Random-effects tobit regression Number of obs = 297

Uncensored = 176

Limits: Lower = ln(125.0001) Left-censored = 111

Upper = ln(24000) Right-censored = 10

Group variable: RecordID Number of groups = 84

Random effects u_i ~ Gaussian Obs per group:

min = 1

avg = 3.5

max = 4

Integration method: mvaghermite Integration pts. = 12

Wald chi2(8) = 36.84

Log likelihood = -417.56162 Prob > chi2 = 0.0000

---------------------------------------------------------------------------------

ln_VEGFR1 | Coefficient Std. err. z P>|z| [95% conf. interval]

----------------+----------------------------------------------------------------

time |

3 | -.2810491 .276463 -1.02 0.309 -.8229067 .2608085

6 | -.3166984 .2847016 -1.11 0.266 -.8747033 .2413065

24 | .7709503 .2906634 2.65 0.008 .2012605 1.34064

|

restricted |

Restricted | -.0127536 .547765 -0.02 0.981 -1.086353 1.060846

|

time#restricted |

3#Restricted | .1768026 .388034 0.46 0.649 -.5837301 .9373353

6#Restricted | .058629 .3933803 0.15 0.882 -.7123823 .8296403

24#Restricted | -.0780928 .3940409 -0.20 0.843 -.8503989 .6942133

|

pscore | -6.107198 2.561699 -2.38 0.017 -11.12803 -1.086361

_cons | 9.260917 1.374319 6.74 0.000 6.567301 11.95453

----------------+----------------------------------------------------------------

/sigma_u | 2.089419 .2103674 9.93 0.000 1.677106 2.501732

/sigma_e | 1.043385 .070895 14.72 0.000 .9044331 1.182336

----------------+----------------------------------------------------------------

rho | .8004057 .0372242 .7197682 .8652012

---------------------------------------------------------------------------------

LR test of sigma_u=0: chibar2(01) = 189.57 Prob >= chibar2 = 0.000

. testparm time#restricted

( 1) [ln_VEGFR1]3.time#1.restricted = 0

( 2) [ln_VEGFR1]6.time#1.restricted = 0

( 3) [ln_VEGFR1]24.time#1.restricted = 0

chi2( 3) = 0.44

Prob > chi2 = 0.9323

. xttobit ln_VEGFR1 i.time i.restricted if per_protocol==1, ll(ln(125.0001)) ul(ln(24000)) nolog

Random-effects tobit regression Number of obs = 313

Uncensored = 186

Limits: Lower = ln(125.0001) Left-censored = 117

Upper = ln(24000) Right-censored = 10

Group variable: RecordID Number of groups = 89

Random effects u_i ~ Gaussian Obs per group:

min = 1

avg = 3.5

max = 4

Integration method: mvaghermite Integration pts. = 12

Wald chi2(4) = 32.92

Log likelihood = -441.36673 Prob > chi2 = 0.0000

------------------------------------------------------------------------------

ln_VEGFR1 | Coefficient Std. err. z P>|z| [95% conf. interval]

-------------+----------------------------------------------------------------

time |

3 | -.1812949 .1876985 -0.97 0.334 -.5491772 .1865875

6 | -.246483 .1907362 -1.29 0.196 -.6203191 .127353

24 | .7426252 .1901488 3.91 0.000 .3699404 1.11531

|

restricted |

Restricted | -.2685263 .4822945 -0.56 0.578 -1.213806 .6767535

_cons | 6.092439 .3709969 16.42 0.000 5.365298 6.81958

-------------+----------------------------------------------------------------

/sigma_u | 2.132409 .2084078 10.23 0.000 1.723937 2.540881

/sigma_e | 1.034577 .0684111 15.12 0.000 .9004936 1.16866

-------------+----------------------------------------------------------------

rho | .8094622 .0349092 .7337579 .8702586

------------------------------------------------------------------------------

LR test of sigma_u=0: chibar2(01) = 206.49 Prob >= chibar2 = 0.000

. xttobit ln_VEGFR1 i.time i.restricted pscore if per_protocol==1, ll(ln(125.0001)) ul(ln(24000)) nolog

Random-effects tobit regression Number of obs = 297

Uncensored = 176

Limits: Lower = ln(125.0001) Left-censored = 111

Upper = ln(24000) Right-censored = 10

Group variable: RecordID Number of groups = 84

Random effects u_i ~ Gaussian Obs per group:

min = 1

avg = 3.5

max = 4

Integration method: mvaghermite Integration pts. = 12

Wald chi2(5) = 36.41

Log likelihood = -417.78069 Prob > chi2 = 0.0000

------------------------------------------------------------------------------

ln_VEGFR1 | Coefficient Std. err. z P>|z| [95% conf. interval]

-------------+----------------------------------------------------------------

time |

3 | -.1932611 .1940496 -1.00 0.319 -.5735913 .1870692

6 | -.2878203 .1966004 -1.46 0.143 -.67315 .0975094

24 | .7267078 .1972462 3.68 0.000 .3401123 1.113303

|

restricted |

Restricted | .0247109 .4964783 0.05 0.960 -.9483687 .9977906

pscore | -6.107506 2.561874 -2.38 0.017 -11.12869 -1.086326

_cons | 9.241565 1.368976 6.75 0.000 6.558422 11.92471

-------------+----------------------------------------------------------------

/sigma_u | 2.089634 .2104491 9.93 0.000 1.677161 2.502107

/sigma_e | 1.044168 .0709597 14.71 0.000 .9050892 1.183246

-------------+----------------------------------------------------------------

rho | .8001989 .0372695 .7194667 .8650744

------------------------------------------------------------------------------

LR test of sigma_u=0: chibar2(01) = 189.30 Prob >= chibar2 = 0.000

# Heparan Sulphate

. bysort restricted:tabstat HeparanSulfateT ln_HepSulfate if per_protocol==1, ///

> s(n mean sd median min max p25 p75) by(time) col(stats) long f(%7.5g)

-> restricted = Standard

time Variable | N Mean SD p50 Min Max p25 p75

--------------------+------------------------------------------------------------------------

0 HeparanSul~T | 39 2653 1767.9 2247 476.65 7108 1457.5 3649.6

ln_HepSulf~e | 39 7.6763 .66147 7.7174 6.1668 8.869 7.2845 8.2024

--------------------+------------------------------------------------------------------------

3 HeparanSul~T | 39 2768.5 1706.1 2219.5 574.88 7019.2 1353 4242.4

ln_HepSulf~e | 39 7.7228 .67132 7.705 6.3542 8.8564 7.2101 8.3529

--------------------+------------------------------------------------------------------------

6 HeparanSul~T | 35 2768.8 1781.3 2064.8 435.6 7331.2 1456 4048

ln_HepSulf~e | 35 7.7191 .67743 7.6328 6.0767 8.8999 7.2834 8.306

--------------------+------------------------------------------------------------------------

24 HeparanSul~T | 35 2640.1 1651 2224 756.88 6971.2 1209.6 3644.8

ln_HepSulf~e | 35 7.6896 .63089 7.7071 6.6292 8.8495 7.098 8.2011

--------------------+------------------------------------------------------------------------

Total HeparanSul~T | 148 2707.8 1711.3 2217.8 435.6 7331.2 1385.7 3722

ln_HepSulf~e | 148 7.7018 .65437 7.7042 6.0767 8.8999 7.2339 8.222

---------------------------------------------------------------------------------------------

-> restricted = Restricted

time Variable | N Mean SD p50 Min Max p25 p75

--------------------+------------------------------------------------------------------------

0 HeparanSul~T | 45 2988.1 2092.8 2231.5 524.64 9008 1601.5 3850.4

ln_HepSulf~e | 45 7.7746 .693 7.7104 6.2627 9.1059 7.3787 8.2559

--------------------+------------------------------------------------------------------------

3 HeparanSul~T | 43 2787.5 1799 2112 730 8544 1479.2 3980.8

ln_HepSulf~e | 43 7.7485 .61146 7.6554 6.593 9.053 7.2993 8.2892

--------------------+------------------------------------------------------------------------

6 HeparanSul~T | 41 2979.9 2260.9 2009.6 482.88 7652.8 1138 4536.8

ln_HepSulf~e | 41 7.7008 .80486 7.6057 6.1798 8.9428 7.037 8.42

--------------------+------------------------------------------------------------------------

24 HeparanSul~T | 44 2993.7 1905.1 2500 657.5 8288 1492.8 4118.8

ln_HepSulf~e | 44 7.8114 .63746 7.8228 6.4884 9.0226 7.3084 8.3224

--------------------+------------------------------------------------------------------------

Total HeparanSul~T | 173 2937.7 2003.6 2226.4 482.88 9008 1453.6 3945

ln_HepSulf~e | 173 7.76 .6841 7.7081 6.1798 9.1059 7.2818 8.2802

---------------------------------------------------------------------------------------------

. mixed ln_HepSulfate i.time##restricted if per_protocol==1 ||RecordID:, mle

Mixed-effects ML regression Number of obs = 321

Group variable: RecordID Number of groups = 89

Obs per group:

min = 1

avg = 3.6

max = 4

Wald chi2(7) = 1.62

Log likelihood = -203.81507 Prob > chi2 = 0.9778

---------------------------------------------------------------------------------

ln_HepSulfate | Coefficient Std. err. z P>|z| [95% conf. interval]

----------------+----------------------------------------------------------------

time |

3 | .0440292 .0734022 0.60 0.549 -.0998365 .187895

6 | .0049328 .0762902 0.06 0.948 -.1445934 .1544589

24 | .0015363 .0768402 0.02 0.984 -.1490676 .1521403

|

restricted |

Restricted | .1005079 .142273 0.71 0.480 -.1783422 .3793579

|

time#restricted |

3#Restricted | -.0757979 .1012757 -0.75 0.454 -.2742945 .1226988

6#Restricted | -.0724509 .1039159 -0.70 0.486 -.2761222 .1312204

24#Restricted | -.0242332 .1034623 -0.23 0.815 -.2270155 .1785491

|

_cons | 7.676715 .1043509 73.57 0.000 7.472191 7.881239

---------------------------------------------------------------------------------

------------------------------------------------------------------------------

Random-effects parameters | Estimate Std. err. [95% conf. interval]

-----------------------------+------------------------------------------------

RecordID: Identity |

var(_cons) | .3342162 .0545845 .2426658 .4603058

-----------------------------+------------------------------------------------

var(Residual) | .1040695 .0096471 .0867797 .1248042

------------------------------------------------------------------------------

LR test vs. linear model: chibar2(01) = 244.01 Prob >= chibar2 = 0.0000

. testparm time#restricted

( 1) [ln_HepSulfate]3.time#1.restricted = 0

( 2) [ln_HepSulfate]6.time#1.restricted = 0

( 3) [ln_HepSulfate]24.time#1.restricted = 0

chi2( 3) = 0.78

Prob > chi2 = 0.8535

. mixed ln_HepSulfate i.time##restricted if pscore~=. & per_protocol==1 ||RecordID:, mle

Mixed-effects ML regression Number of obs = 305

Group variable: RecordID Number of groups = 84

Obs per group:

min = 1

avg = 3.6

max = 4

Wald chi2(7) = 1.72

Log likelihood = -194.2692 Prob > chi2 = 0.9737

---------------------------------------------------------------------------------

ln_HepSulfate | Coefficient Std. err. z P>|z| [95% conf. interval]

----------------+----------------------------------------------------------------

time |

3 | .0550814 .0744512 0.74 0.459 -.0908402 .2010031

6 | .003612 .0774669 0.05 0.963 -.1482203 .1554443

24 | .0044201 .0782015 0.06 0.955 -.148852 .1576922

|

restricted |

Restricted | .0854993 .1469986 0.58 0.561 -.2026126 .3736112

|

time#restricted |

3#Restricted | -.0836714 .1038797 -0.81 0.421 -.2872719 .119929

6#Restricted | -.0726622 .1062213 -0.68 0.494 -.2808521 .1355277

24#Restricted | -.0407457 .1062252 -0.38 0.701 -.2489433 .1674519

|

_cons | 7.700238 .1071569 71.86 0.000 7.490214 7.910262

---------------------------------------------------------------------------------

------------------------------------------------------------------------------

Random-effects parameters | Estimate Std. err. [95% conf. interval]

-----------------------------+------------------------------------------------

RecordID: Identity |

var(_cons) | .3399517 .0569895 .2447495 .4721856

-----------------------------+------------------------------------------------

var(Residual) | .1042917 .0099059 .0865766 .1256318

------------------------------------------------------------------------------

LR test vs. linear model: chibar2(01) = 234.97 Prob >= chibar2 = 0.0000

. testparm time#restricted

( 1) [ln_HepSulfate]3.time#1.restricted = 0

( 2) [ln_HepSulfate]6.time#1.restricted = 0

( 3) [ln_HepSulfate]24.time#1.restricted = 0

chi2( 3) = 0.77

Prob > chi2 = 0.8557

. mixed ln_HepSulfate i.time##restricted pscore if per_protocol==1 ||RecordID:, mle

Mixed-effects ML regression Number of obs = 305

Group variable: RecordID Number of groups = 84

Obs per group:

min = 1

avg = 3.6

max = 4

Wald chi2(8) = 15.29

Log likelihood = -187.99961 Prob > chi2 = 0.0537

---------------------------------------------------------------------------------

ln_HepSulfate | Coefficient Std. err. z P>|z| [95% conf. interval]

----------------+----------------------------------------------------------------

time |

3 | .0568434 .0744926 0.76 0.445 -.0891594 .2028461

6 | .0078766 .0774949 0.10 0.919 -.1440106 .1597639

24 | .0048069 .0782236 0.06 0.951 -.1485086 .1581224

|

restricted |

Restricted | -.0034469 .1403001 -0.02 0.980 -.27843 .2715362

|

time#restricted |

3#Restricted | -.083567 .1039225 -0.80 0.421 -.2872513 .1201173

6#Restricted | -.0782214 .1062642 -0.74 0.462 -.2864954 .1300527

24#Restricted | -.0400724 .1062531 -0.38 0.706 -.2483246 .1681799

|

pscore | 2.391863 .6490833 3.68 0.000 1.119683 3.664043

_cons | 6.46651 .3495571 18.50 0.000 5.781391 7.15163

---------------------------------------------------------------------------------

------------------------------------------------------------------------------

Random-effects parameters | Estimate Std. err. [95% conf. interval]

-----------------------------+------------------------------------------------

RecordID: Identity |

var(_cons) | .2874754 .0491072 .2056822 .4017952

-----------------------------+------------------------------------------------

var(Residual) | .1044249 .0099308 .0866671 .1258211

------------------------------------------------------------------------------

LR test vs. linear model: chibar2(01) = 201.94 Prob >= chibar2 = 0.0000

. testparm time#restricted

( 1) [ln_HepSulfate]3.time#1.restricted = 0

( 2) [ln_HepSulfate]6.time#1.restricted = 0

( 3) [ln_HepSulfate]24.time#1.restricted = 0

chi2( 3) = 0.82

Prob > chi2 = 0.8441

. mixed ln_HepSulfate i.time i.restricted if per_protocol==1||RecordID:, mle

Mixed-effects ML regression Number of obs = 321

Group variable: RecordID Number of groups = 89

Obs per group:

min = 1

avg = 3.6

max = 4

Wald chi2(4) = 0.84

Log likelihood = -204.20608 Prob > chi2 = 0.9335

-------------------------------------------------------------------------------

ln_HepSulfate | Coefficient Std. err. z P>|z| [95% conf. interval]

--------------+----------------------------------------------------------------

time |

3 | .0041201 .0506449 0.08 0.935 -.0951421 .1033824

6 | -.0338006 .0518721 -0.65 0.515 -.135468 .0678669

24 | -.0110981 .05152 -0.22 0.829 -.1120754 .0898791

|

restricted |

Restricted | .0585691 .1285961 0.46 0.649 -.1934746 .3106129

_cons | 7.699055 .0991771 77.63 0.000 7.504672 7.893439

-------------------------------------------------------------------------------

------------------------------------------------------------------------------

Random-effects parameters | Estimate Std. err. [95% conf. interval]

-----------------------------+------------------------------------------------

RecordID: Identity |

var(_cons) | .3345111 .0546354 .242876 .4607194

-----------------------------+------------------------------------------------

var(Residual) | .1043785 .0096753 .0870381 .1251736

------------------------------------------------------------------------------

LR test vs. linear model: chibar2(01) = 243.78 Prob >= chibar2 = 0.0000

. mixed ln_HepSulfate i.time i.restricted pscore if per_protocol==1 ||RecordID:, mle

Mixed-effects ML regression Number of obs = 305

Group variable: RecordID Number of groups = 84

Obs per group:

min = 1

avg = 3.6

max = 4

Wald chi2(5) = 14.45

Log likelihood = -188.41026 Prob > chi2 = 0.0130

-------------------------------------------------------------------------------

ln_HepSulfate | Coefficient Std. err. z P>|z| [95% conf. interval]

--------------+----------------------------------------------------------------

time |

3 | .0137602 .0520254 0.26 0.791 -.0882078 .1157281

6 | -.033364 .0531028 -0.63 0.530 -.1374436 .0707157

24 | -.0160971 .0530128 -0.30 0.761 -.1200002 .087806

|

restricted |

Restricted | -.0526386 .1258348 -0.42 0.676 -.2992701 .193993

pscore | 2.387769 .6493782 3.68 0.000 1.115011 3.660527

_cons | 6.494441 .34805 18.66 0.000 5.812275 7.176606

-------------------------------------------------------------------------------

------------------------------------------------------------------------------

Random-effects parameters | Estimate Std. err. [95% conf. interval]

-----------------------------+------------------------------------------------

RecordID: Identity |

var(_cons) | .2877342 .0491514 .2058672 .4021571

-----------------------------+------------------------------------------------

var(Residual) | .1047687 .0099627 .0869538 .1262334

------------------------------------------------------------------------------

LR test vs. linear model: chibar2(01) = 201.83 Prob >= chibar2 = 0.0000

# Syn 4 (censored values)

T0 n=4 Below T3 n=2 Below T6 n=4 Below T24 n=5 Below

None above limit.

. bysort restricted:tabstat Syn4 ln_Syn4 if per_protocol==1, ///

> s(n mean sd median min max p25 p75) by(time) col(stats) long f(%7.4g)

-> restricted = Standard

time Variable | N Mean SD p50 Min Max p25 p75

-----------------+--------------------------------------------------------------------------

0 Syn4 | 39 2360 3774 807.2 113.8 19405 435.2 2137

ln_Syn4 | 39 6.999 1.217 6.694 4.735 9.873 6.076 7.667

-----------------+--------------------------------------------------------------------------

3 Syn4 | 39 3022 5083 1285 259.9 22680 485.5 2548

ln_Syn4 | 39 7.197 1.179 7.159 5.56 10.03 6.185 7.843

-----------------+--------------------------------------------------------------------------

6 Syn4 | 35 2241 3811 690.9 113.8 16010 401.3 1628

ln_Syn4 | 35 6.857 1.199 6.538 4.735 9.681 5.995 7.395

-----------------+--------------------------------------------------------------------------

24 Syn4 | 35 2326 4682 559.3 113.8 24000 270.9 1329

ln_Syn4 | 35 6.605 1.412 6.327 4.735 10.09 5.602 7.192

-----------------+--------------------------------------------------------------------------

Total Syn4 | 148 2498 4346 792.4 113.8 24000 413.9 2048

ln_Syn4 | 148 6.925 1.258 6.675 4.735 10.09 6.026 7.624

--------------------------------------------------------------------------------------------

-> restricted = Restricted

time Variable | N Mean SD p50 Min Max p25 p75

----------------+-------------------------------------------------------------------------

0 Syn4 | 45 2656 6088 866.8 113.8 39325 483.6 2038

ln_Syn4 | 45 6.964 1.224 6.765 4.735 10.58 6.181 7.62

----------------+-------------------------------------------------------------------------

3 Syn4 | 43 3455 11903 798 113.8 78250 419.1 1808

ln_Syn4 | 43 6.896 1.273 6.682 4.735 11.27 6.038 7.5

----------------+-------------------------------------------------------------------------

6 Syn4 | 41 1953 4581 574.6 113.8 29000 361.1 1885

ln_Syn4 | 41 6.67 1.206 6.354 4.735 10.28 5.889 7.542

----------------+-------------------------------------------------------------------------

24 Syn4 | 44 2856 8195 724.3 113.8 52150 321 1594

ln_Syn4 | 44 6.765 1.314 6.583 4.735 10.86 5.771 7.371

----------------+-------------------------------------------------------------------------

Total Syn4 |173 2739 8126 790.2 113.8 78250 387.4 1768

ln_Syn4 |173 6.827 1.25 6.672 4.735 11.27 5.959 7.478

------------------------------------------------------------------------------------------

. xttobit ln_Syn4 i.time##restricted if per_protocol==1, ll(ln(113.85)) nolog

Random-effects tobit regression Number of obs = 321

Uncensored = 321

. xttobit ln_Syn4 i.time##restricted if per_protocol==1, ll(ln(113.86)) nolog

Random-effects tobit regression Number of obs = 321

Uncensored = 306

Limits: Lower = ln(113.86) Left-censored = 15

Upper = +inf Right-censored = 0

Group variable: RecordID Number of groups = 89

Random effects u_i ~ Gaussian Obs per group:

min = 1

avg = 3.6

max = 4

Integration method: mvaghermite Integration pts. = 12

Wald chi2(7) = 36.47

Log likelihood = -380.68052 Prob > chi2 = 0.0000

---------------------------------------------------------------------------------

ln_Syn4 | Coefficient Std. err. z P>|z| [95% conf. interval]

----------------+----------------------------------------------------------------

time |

3 | .1841803 .1214728 1.52 0.129 -.0539019 .4222625

6 | -.1936798 .1266419 -1.53 0.126 -.4418933 .0545338

24 | -.4715004 .1285237 -3.67 0.000 -.7234021 -.2195986

|

restricted |

Restricted | -.0018748 .2735454 -0.01 0.995 -.5380139 .5342642

|

time#restricted |

3#Restricted | -.3086078 .1682767 -1.83 0.067 -.638424 .0212083

6#Restricted | -.0744509 .1731065 -0.43 0.667 -.4137334 .2648317

24#Restricted | .1843142 .1727985 1.07 0.286 -.1543647 .5229931

|

_cons | 6.978623 .2005488 34.80 0.000 6.585554 7.371691

----------------+----------------------------------------------------------------

/sigma_u | 1.15765 .0931697 12.43 0.000 .975041 1.340259

/sigma_e | .5327365 .0255529 20.85 0.000 .4826539 .5828192

----------------+----------------------------------------------------------------

rho | .8252372 .0272712 .7666865 .8734893

---------------------------------------------------------------------------------

LR test of sigma_u=0: chibar2(01) = 300.91 Prob >= chibar2 = 0.000

. testparm time#restricted

( 1) [ln_Syn4]3.time#1.restricted = 0

( 2) [ln_Syn4]6.time#1.restricted = 0

( 3) [ln_Syn4]24.time#1.restricted = 0

chi2( 3) = 8.30

Prob > chi2 = 0.0402

. xttobit ln_Syn4 i.time##restricted if per_protocol==1, ll(ln(113.86)) nolog ///

> vce(bootstrap, reps(500) seed(010967))

(running xttobit on estimation sample)

Random-effects tobit regression Number of obs = 321

Uncensored = 306

Limits: Lower = ln(113.86) Left-censored = 15

Upper = +inf Right-censored = 0

Replications = 500

Group variable: RecordID Number of groups = 89

Random effects u_i ~ Gaussian Obs per group:

min = 1

avg = 3.6

max = 4

Integration method: mvaghermite Integration pts. = 12

Wald chi2(7) = 27.67

Log likelihood = -380.68052 Prob > chi2 = 0.0003

(Replications based on 89 clusters in RecordID)

---------------------------------------------------------------------------------

| Observed Bootstrap Normal-based

ln_Syn4 | coefficient std. err. z P>|z| [95% conf. interval]

----------------+----------------------------------------------------------------

time |

3 | .1841803 .1093737 1.68 0.092 -.0301883 .3985489

6 | -.1936798 .1093966 -1.77 0.077 -.4080931 .0207335

24 | -.4715004 .1654788 -2.85 0.004 -.7958329 -.1471678

|

restricted |

Restricted | -.0018748 .2704773 -0.01 0.994 -.5320006 .528251

|

time#restricted |

3#Restricted | -.3086078 .1404496 -2.20 0.028 -.5838839 -.0333318

6#Restricted | -.0744509 .1757935 -0.42 0.672 -.4189999 .2700981

24#Restricted | .1843142 .201962 0.91 0.361 -.211524 .5801524

|

_cons | 6.978623 .1955539 35.69 0.000 6.595344 7.361901

----------------+----------------------------------------------------------------

/sigma_u | 1.15765 .1203822 9.62 0.000 .9217055 1.393595

/sigma_e | .5327365 .0376569 14.15 0.000 .4589304 .6065427

----------------+----------------------------------------------------------------

rho | .8252372 .0363107 .7451124 .8872116

---------------------------------------------------------------------------------

LR test of sigma_u=0: chibar2(01) = 300.91 Prob >= chibar2 = 0.000

. testparm time#restricted

( 1) [ln_Syn4]3.time#1.restricted = 0

( 2) [ln_Syn4]6.time#1.restricted = 0

( 3) [ln_Syn4]24.time#1.restricted = 0

chi2( 3) = 9.62

Prob > chi2 = 0.0221

. xttobit ln_Syn4 b24.time##restricted if per_protocol==1, ll(ln(113.86)) nolog ///

> vce(bootstrap, reps(500) seed(010967))

(running xttobit on estimation sample)

Random-effects tobit regression Number of obs = 321

Uncensored = 306

Limits: Lower = ln(113.86) Left-censored = 15

Upper = +inf Right-censored = 0

Replications = 500

Group variable: RecordID Number of groups = 89

Random effects u_i ~ Gaussian Obs per group:

min = 1

avg = 3.6

max = 4

Integration method: mvaghermite Integration pts. = 12

Wald chi2(7) = 27.67

Log likelihood = -380.68052 Prob > chi2 = 0.0003

(Replications based on 89 clusters in RecordID)

---------------------------------------------------------------------------------

| Observed Bootstrap Normal-based

ln_Syn4 | coefficient std. err. z P>|z| [95% conf. interval]

----------------+----------------------------------------------------------------

time |

0 | .4715004 .1654788 2.85 0.004 .1471678 .7958329

3 | .6556807 .1544248 4.25 0.000 .3530136 .9583477

6 | .2778206 .1253653 2.22 0.027 .032109 .5235322

|

restricted |

Restricted | .1824394 .2998892 0.61 0.543 -.4053327 .7702114

|

time#restricted |

0#Restricted | -.1843142 .201962 -0.91 0.361 -.5801524 .211524

3#Restricted | -.492922 .1854786 -2.66 0.008 -.8564533 -.1293907

6#Restricted | -.258765 .1800671 -1.44 0.151 -.61169 .0941599

|

_cons | 6.507122 .2356655 27.61 0.000 6.045226 6.969018

----------------+----------------------------------------------------------------

/sigma_u | 1.15765 .1203822 9.62 0.000 .9217055 1.393595

/sigma_e | .5327365 .0376569 14.15 0.000 .4589304 .6065427

----------------+----------------------------------------------------------------

rho | .8252372 .0363107 .7451124 .8872116

---------------------------------------------------------------------------------

LR test of sigma_u=0: chibar2(01) = 300.91 Prob >= chibar2 = 0.000

. xttobit ln_Syn4 i.time##restricted if pscore~=. & per_protocol==1, ll(ln(113.86)) nolog ///

> vce(bootstrap, reps(500) seed(010967))

(running xttobit on estimation sample)

Random-effects tobit regression Number of obs = 305

Uncensored = 290

Limits: Lower = ln(113.86) Left-censored = 15

Upper = +inf Right-censored = 0

Replications = 500

Group variable: RecordID Number of groups = 84

Random effects u_i ~ Gaussian Obs per group:

min = 1

avg = 3.6

max = 4

Integration method: mvaghermite Integration pts. = 12

Wald chi2(7) = 31.27

Log likelihood = -361.30696 Prob > chi2 = 0.0001

(Replications based on 84 clusters in RecordID)

---------------------------------------------------------------------------------

| Observed Bootstrap Normal-based

ln_Syn4 | coefficient std. err. z P>|z| [95% conf. interval]

----------------+----------------------------------------------------------------

time |

3 | .1742581 .1073204 1.62 0.104 -.036086 .3846022

6 | -.2014507 .1030103 -1.96 0.051 -.4033471 .0004457

24 | -.4981105 .1679001 -2.97 0.003 -.8271887 -.1690323

|

restricted |

Restricted | -.0206252 .2895143 -0.07 0.943 -.5880629 .5468124

|

time#restricted |

3#Restricted | -.2642518 .1428958 -1.85 0.064 -.5443224 .0158188

6#Restricted | -.0708965 .1685497 -0.42 0.674 -.4012479 .2594549

24#Restricted | .2265177 .2001496 1.13 0.258 -.1657684 .6188038

|

_cons | 6.994894 .2024176 34.56 0.000 6.598163 7.391625

----------------+----------------------------------------------------------------

/sigma_u | 1.189363 .1249699 9.52 0.000 .9444262 1.434299

/sigma_e | .5275776 .0413469 12.76 0.000 .4465392 .6086159

----------------+----------------------------------------------------------------

rho | .8355871 .0370087 .7528854 .8978447

---------------------------------------------------------------------------------

LR test of sigma_u=0: chibar2(01) = 297.57 Prob >= chibar2 = 0.000

. testparm time#restricted

( 1) [ln_Syn4]3.time#1.restricted = 0

( 2) [ln_Syn4]6.time#1.restricted = 0

( 3) [ln_Syn4]24.time#1.restricted = 0

chi2( 3) = 7.59

Prob > chi2 = 0.055

. xttobit ln_Syn4 i.time##restricted pscore if per_protocol==1, ll(ln(113.86)) nolog ///

> vce(bootstrap, reps(500) seed(010967))

(running xttobit on estimation sample)

Random-effects tobit regression Number of obs = 305

Uncensored = 290

Limits: Lower = ln(113.86) Left-censored = 15

Upper = +inf Right-censored = 0

Replications = 500

Group variable: RecordID Number of groups = 84

Random effects u_i ~ Gaussian Obs per group:

min = 1

avg = 3.6

max = 4

Integration method: mvaghermite Integration pts. = 12

Wald chi2(8) = 35.29

Log likelihood = -360.56289 Prob > chi2 = 0.0000

(Replications based on 84 clusters in RecordID)

---------------------------------------------------------------------------------

| Observed Bootstrap Normal-based

ln_Syn4 | coefficient std. err. z P>|z| [95% conf. interval]

----------------+----------------------------------------------------------------

time |

3 | .1735073 .1073633 1.62 0.106 -.0369208 .3839355

6 | -.2026952 .1029622 -1.97 0.049 -.4044974 -.000893

24 | -.4976121 .1678253 -2.97 0.003 -.8265436 -.1686806

|

restricted |

Restricted | .0434458 .2991765 0.15 0.885 -.5429294 .629821

|

time#restricted |

3#Restricted | -.2640758 .1429143 -1.85 0.065 -.5441826 .0160311

6#Restricted | -.0690931 .1684042 -0.41 0.682 -.3991593 .2609732

24#Restricted | .2262762 .2000437 1.13 0.258 -.1658023 .6183547

|

pscore | -1.710752 1.840356 -0.93 0.353 -5.317785 1.89628

_cons | 7.877087 1.014068 7.77 0.000 5.88955 9.864623

----------------+----------------------------------------------------------------

/sigma_u | 1.178207 .1168161 10.09 0.000 .9492513 1.407162

/sigma_e | .5273764 .0413129 12.77 0.000 .4464045 .6083483

----------------+----------------------------------------------------------------

rho | .8330874 .0357266 .753784 .8936967

---------------------------------------------------------------------------------

LR test of sigma_u=0: chibar2(01) = 295.80 Prob >= chibar2 = 0.000

. testparm time#restricted

( 1) [ln_Syn4]3.time#1.restricted = 0

( 2) [ln_Syn4]6.time#1.restricted = 0

( 3) [ln_Syn4]24.time#1.restricted = 0

chi2( 3) = 7.58

Prob > chi2 = 0.0555
