# Supplementary material for: Effect of intravenous fluid volume on biomarkers of endothelial glycocalyx shedding and inflammation during initial resuscitation of sepsis
Source: Intensive Care Med Exp. 2023 Apr 17;11:21. doi: 10.1186/s40635-023-00508-4 (PMC10106534; doi:10.1186/s40635-023-00508-4)
Supplement: Supplementary file 2 — Additional file 2. Analysis of biomarkers from refresh RCT. [file 40635_2023_508_MOESM2_ESM.docx]

# Analysis of Biomarkers from Refresh RCT

There were 4 patients with no blood samples, 3 of which were in the standard group and 1 in the restricted group.

. tab restricted if time==.

restricted | Freq. Percent Cum.

------------+-----------------------------------

0 | 3 75.00 75.00

1 | 1 25.00 100.00

------------+-----------------------------------

Total | 4 100.00

. tab restricted if time~=. & rec_num==1

restricted | Freq. Percent Cum.

------------+-----------------------------------

0 | 46 48.42 48.42

1 | 49 51.58 100.00

------------+-----------------------------------

Total | 95 100.00

There are some additional missing for individual biomarkers.

# About the output.

For each biomarker the following format has been used:

- Summary statistics for the raw data and the log transformed data by time and group
- Graphical display of the above in the form of box and whisker plots by time and group
- Statistical modelling: All variables have been log transformed for analysis. There were 4 variables (ProANP, ICAM, E-selectin, VCAM) where all values were within the assay limits. For these variables, linear mixed models have been performed on the log transformed variable. These are presented first in the document. For the remaining variables, random effects Tobit regression was used. For each biomarker the following analyses were run:
  - A model with the interaction of group and time. This is the primary question of interest – whether the change over time in the biomarker differs significantly between the two groups.
  - An overall test of the interaction term has been added after the model above to provide some protection from false positives that may arise from only considering the pairwise comparisons and also to check for significance of pairwise comparisons that are not directly displayed in the output.
  - If a significant or close to significant p value was detected for the interaction term, the model was bootstrapped to investigate sensitivity to sampling variation. If the study is being written up as a pilot this may not be warranted.
  - A model with time and group (no interaction) is then run. This only addresses whether there was a difference in the biomarker between groups across all values (all times combined) or if there is a difference over time in the whole sample (two groups combined).
  - A propensity score has been generated using Age WeightInkg SBP Lactate i.Sex CharlsonComorbidityScore and SOFA_PEAK. This resulting variable has a slight loss of sample (n=5) due to missingness on Lactate (4 of the 5 from GCU, 2 from the standard group, 3 from restricted). Each of the two models described above were subsequently adjusted for the propensity score. For most models this has no impact on significance. Where there may be some effect, the model was run on the sample with data for the propensity score, without the propensity score included and then again with the propensity score included. This allows the separation of the effect of adjusting for the score and the effect of the loss of sample.

# Summary of Results

| Biomarker | P value for interaction term | Bootstrapped | Pscore effect^*^ |
| --- | --- | --- | --- |
| Ln_ProANP | 0.31 |  | Significantly associated.  Does not impact on intervention effects |
| Ln_ICAM | 0.31 |  | Not significantly associated. |
| Ln_VCAM | 0.26 |  | Not significantly associated. |
| Ln_Eselectin | 0.73 |  | Significantly associated.  Does not impact on intervention effects |
| Ln_NGAL | 0.15 |  | Significantly associated.  Does not impact on intervention effects |
| Ln_Resistin | 0.43 |  | Significantly associated.  Does not impact on intervention effects |
| Ln_Syn1 | 0.92 |  | Not significantly associated. |
| **Ln_Hyaluronan** | **0.04** | 0.053 | Not significantly associated. |
| **Ln_Il6** | **0.07** | 0.059 | Not significantly associated (but very close) |
| Ln_Il10 | 0.22 |  | Not significantly associated. |
| Ln_VEGFR1 | 0.96 |  | Significantly associated.  Does not impact on intervention effects |
| Ln_HepSulfate | 0.72 |  | Significantly associated  Does not impact on intervention effects |
| **Ln_Syn4** | 0.04 | **0.012** | Not significantly associated. |

^*^Significantly associated (or not) is with respect to the outcome i.e.biomarker

For Hyaluronan, the significance is due to differences in the change in ln_Hyaluronan from T3 to T24 and T6 to T24 between the two groups

For IL6, the almost significance is due to difference in the change in Ln_Il6 from T0 to T3 between the two groups (pairwise p value=0.03). T3 to T24 pairwise comparison also <0.05.

Adjustment for the propensity score yielded nothing useful but does result in loss of sample. This analysis should be seen as a robust check that additional adjustment for patient characteristics does not alter the findings – that the randomisation was sufficient.

# ProANP (No censoring)

. bysort restricted:tabstat proANPTn ln_proANP, ///

> s(n mean sd median min max p25 p75) by(time) col(stats) long

-> restricted = Standard

time variable | N mean sd p50 min max p25 p75

-----------------+--------------------------------------------------------------------------

0 proANPTn | 44 34485.93 26864.68 25900 2068 142700 17515 45747.5

ln_proANP | 44 10.18757 .7642188 10.16198 7.634337 11.8685 9.77081 10.72783

-----------------+--------------------------------------------------------------------------

3 proANPTn | 44 30855.5 22773.28 23530 4488 108700 13535 44120

ln_proANP | 44 10.07797 .7465791 10.06602 8.409163 11.59635 9.512338 10.69278

-----------------+--------------------------------------------------------------------------

6 proANPTn | 39 25028 18536.29 19170 5084 74720 12640 30410

ln_proANP | 39 9.899231 .677393 9.861102 8.533854 11.2215 9.444622 10.32253

-----------------+--------------------------------------------------------------------------

24 proANPTn | 39 34655.26 21233.28 32000 4800 84430 18490 55390

ln_proANP | 39 10.22683 .7394902 10.37349 8.476371 11.34368 9.824986 10.92215

-----------------+--------------------------------------------------------------------------

Total proANPTn | 166 31341.39 22823.64 23470 2068 142700 14430 42320

ln_proANP | 166 10.1 .7379566 10.06348 7.634337 11.8685 9.577065 10.65302

--------------------------------------------------------------------------------------------

-> restricted = Restricted

time variable | N mean sd p50 min max p25 p75

-----------------+-------------------------------------------------------------------------

0 proANPTn | 46 40728.11 28790.36 32750 5992 135250 20200 57590

ln_proANP | 46 10.39035 .6872017 10.3966 8.69818 11.81488 9.913438 10.9611

-----------------+-------------------------------------------------------------------------

3 proANPTn | 43 34322.65 30502.68 22580 2731 137900 14000 45690

ln_proANP | 43 10.11557 .8265064 10.02482 7.912423 11.83428 9.546813 10.72963

-----------------+-------------------------------------------------------------------------

6 proANPTn | 42 26901.48 21132.65 22860 4397 86370 11410 34530

ln_proANP | 42 9.893546 .8149732 10.03698 8.388678 11.3664 9.342245 10.44958

-----------------+-------------------------------------------------------------------------

24 proANPTn | 45 32266.64 18332.68 30110 2616 71400 15890 47480

ln_proANP | 45 10.18726 .6895061 10.31261 7.869402 11.17605 9.673446 10.76806

-----------------+-------------------------------------------------------------------------

Total proANPTn |176 33700.16 25500.44 26795 2616 137900 15040 46535

ln_proANP |176 10.15274 .7695025 10.19596 7.869402 11.83428 9.618351 10.74796

-------------------------------------------------------------------------------------------

. mixed ln_proANP i.time##restricted ||RecordID:, mle

Mixed-effects ML regression Number of obs = **342**

Group variable: RecordID Number of groups = **95**

Obs per group:

min = 1

avg = 3.6

max = 4

Wald chi2(7) = 47.28

Log likelihood = -292.1386 Prob > chi2 = 0.0000

---------------------------------------------------------------------------------

ln_proANP | Coef. Std. Err. z P>|z| [95% Conf. Interval]

----------------+----------------------------------------------------------------

time |

3 | -.1306434 .0915101 -1.43 0.153 -.3099999 .0487131

6 | -.324538 .0954025 -3.40 0.001 -.5115234 -.1375527

24 | .0128847 .0959459 0.13 0.893 -.1751659 .2009353

|

1.restricted | .1977152 .1524338 1.30 0.195 -.1010495 .4964799

|

time#restricted |

3 1 | -.1555023 .1298145 -1.20 0.231 -.409934 .0989295

6 1 | -.1906708 .1328038 -1.44 0.151 -.4509614 .0696198

24 1 | -.22994 .1320106 -1.74 0.082 -.488676 .028796

|

_cons | 10.20969 .1092161 93.48 0.000 9.99563 10.42375

---------------------------------------------------------------------------------

------------------------------------------------------------------------------

Random-effects Parameters | Estimate Std. Err. [95% Conf. Interval]

-----------------------------+------------------------------------------------

RecordID: Identity |

var(_cons) | .3536806 .0590843 .2549255 .4906922

-----------------------------+------------------------------------------------

var(Residual) | .1827973 .0164201 .1532883 .2179869

------------------------------------------------------------------------------

LR test vs. linear model: chibar2(01) = 176.85 Prob >= chibar2 = 0.0000

. testparm time#restricted

( 1) [ln_proANP]3.time#1.restricted = 0

( 2) [ln_proANP]6.time#1.restricted = 0

( 3) [ln_proANP]24.time#1.restricted = 0

chi2( 3) = 3.56

Prob > chi2 = 0.3134

**No evidence of an intervention effect was detected for ProANP (p=0.31)**

Including the propensity score (pscore) as a covariate adjustment (which results in a loss of sample).

. mixed ln_proANP i.time##restricted pscore ||RecordID:, mle

Mixed-effects ML regression Number of obs = 326

Group variable: RecordID Number of groups = 90

Obs per group:

min = 1

avg = 3.6

max = 4

Wald chi2(8) = 64.60

Log likelihood = -271.60501 Prob > chi2 = 0.0000

---------------------------------------------------------------------------------

ln_proANP | Coef. Std. Err. z P>|z| [95% Conf. Interval]

----------------+----------------------------------------------------------------

time |

3 | -.1324968 .0931474 -1.42 0.155 -.3150624 .0500688

6 | -.3170797 .0971796 -3.26 0.001 -.5075481 -.1266112

24 | .0018084 .0979856 0.02 0.985 -.1902398 .1938567

|

restricted |

Restricted | .1205533 .1491491 0.81 0.419 -.1717736 .4128801

|

time#restricted |

3#Restricted | -.1828544 .1337514 -1.37 0.172 -.4450023 .0792935

6#Restricted | -.2161531 .1362935 -1.59 0.113 -.4832835 .0509772

24#Restricted | -.2752081 .1360765 -2.02 0.043 -.5419132 -.008503

|

pscore | 2.39596 .5612044 4.27 0.000 1.296019 3.4959

_cons | 9.056309 .2915132 31.07 0.000 8.484953 9.627664

---------------------------------------------------------------------------------

------------------------------------------------------------------------------

Random-effects Parameters | Estimate Std. Err. [95% Conf. Interval]

-----------------------------+------------------------------------------------

RecordID: Identity |

var(_cons) | .2854844 .0508879 .2013051 .4048648

-----------------------------+------------------------------------------------

var(Residual) | .1851166 .0170483 .1545446 .2217363

------------------------------------------------------------------------------

LR test vs. linear model: chibar2(01) = 134.71 Prob >= chibar2 = 0.0000

. testparm time#restricted

( 1) [ln_proANP]3.time#1.restricted = 0

( 2) [ln_proANP]6.time#1.restricted = 0

( 3) [ln_proANP]24.time#1.restricted = 0

chi2( 3) = 4.66

Prob > chi2 = 0.1987

The p value is slightly smaller but still not significant. The smaller p value is generated from the change in sample (analysis not shown).

. mixed ln_proANP i.time i.restricted ||RecordID:, mle

Computing standard errors:

Mixed-effects ML regression Number of obs = 342

Group variable: RecordID Number of groups = 95

Obs per group:

min = 1

avg = 3.6

max = 4

Wald chi2(4) = 43.12

Log likelihood = -293.90538 Prob > chi2 = 0.0000

------------------------------------------------------------------------------

ln_proANP | Coef. Std. Err. z P>|z| [95% Conf. Interval]

-------------+----------------------------------------------------------------

time |

3 | -.2092244 .0653488 -3.20 0.001 -.3373058 -.081143

6 | -.4230442 .0668204 -6.33 0.000 -.5540098 -.2920786

24 | -.1078508 .0663391 -1.63 0.104 -.2378732 .0221715

|

1.restricted | .0572289 .1312011 0.44 0.663 -.1999206 .3143784

_cons | 10.2814 .1019174 100.88 0.000 10.08165 10.48116

------------------------------------------------------------------------------

------------------------------------------------------------------------------

Random-effects Parameters | Estimate Std. Err. [95% Conf. Interval]

-----------------------------+------------------------------------------------

RecordID: Identity |

var(_cons) | .3533988 .0591485 .2545653 .4906039

-----------------------------+------------------------------------------------

var(Residual) | .1853482 .0166485 .1554287 .2210272

------------------------------------------------------------------------------

LR test vs. linear model: chibar2(01) = 174.71 Prob >= chibar2 = 0.0000

Ln_ProANP is varying significantly over time. No difference in the average ln_ProANP (across all time periods) was detected between the two groups.

. mixed ln_proANP i.time i.restricted pscore ||RecordID:, mle

Mixed-effects ML regression Number of obs = 326

Group variable: RecordID Number of groups = 90

Obs per group:

min = 1

avg = 3.6

max = 4

Wald chi2(5) = 59.12

Log likelihood = -273.91198 Prob > chi2 = 0.0000

------------------------------------------------------------------------------

ln_proANP | Coef. Std. Err. z P>|z| [95% Conf. Interval]

-------------+----------------------------------------------------------------

time |

3 | -.222736 .0674786 -3.30 0.001 -.3549916 -.0904805

6 | -.4265609 .0687699 -6.20 0.000 -.5613474 -.2917744

24 | -.1399342 .068619 -2.04 0.041 -.2744249 -.0054434

|

restricted |

Restricted | -.042941 .1261836 -0.34 0.734 -.2902563 .2043743

pscore | 2.39245 .5614081 4.26 0.000 1.29211 3.49279

_cons | 9.139294 .2887903 31.65 0.000 8.573275 9.705313

------------------------------------------------------------------------------

------------------------------------------------------------------------------

Random-effects Parameters | Estimate Std. Err. [95% Conf. Interval]

-----------------------------+------------------------------------------------

RecordID: Identity |

var(_cons) | .2848896 .0509443 .2006613 .404473

-----------------------------+------------------------------------------------

var(Residual) | .1886779 .0173744 .1575209 .2259977

------------------------------------------------------------------------------

LR test vs. linear model: chibar2(01) = 132.25 Prob >= chibar2 = 0.0000

Adjusting for the propensity score has made no substantive alteration to the conclusion

# ICAM

. bysort restricted:tabstat ICAMTn ln_ICAM, ///

> s(n mean sd median min max p25 p75) by(time) col(stats) long

-> restricted = Standard

time variable | N mean sd p50 min max p25 p75

----------------+--------------------------------------------------------------------------

0 ICAMTn | 44 1070.543 953.6027 681.241 99.95471 4152.3 456.0436 1272.107

ln_ICAM | 44 6.604019 .9078751 6.523763 4.604717 8.331418 6.122284 7.147039

----------------+--------------------------------------------------------------------------

3 ICAMTn | 44 912.4125 748.8956 608.8663 87.05 2855.245 451.2675 1070.662

ln_ICAM | 44 6.513314 .8059637 6.411583 4.466483 7.956913 6.111347 6.975377

----------------+--------------------------------------------------------------------------

6 ICAMTn | 39 1331.896 1855.153 811.22 183.54 10537.6 479.86 1362.28

ln_ICAM | 39 6.735667 .8895939 6.698539 5.212433 9.262705 6.173494 7.216915

----------------+--------------------------------------------------------------------------

24 ICAMTn | 39 1075.052 1088.326 598.54 111.95 4093.88 360.86 1363.09

ln_ICAM | 39 6.536676 .9687664 6.394494 4.718052 8.317248 5.88849 7.217509

----------------+--------------------------------------------------------------------------

Total ICAMTn | 166 1091.09 1212.819 698.1327 87.05 10537.6 434.235 1339.142

ln_ICAM | 166 6.595085 .8886872 6.548384 4.466483 9.262705 6.073586 7.199784

-------------------------------------------------------------------------------------------

-> restricted = Restricted

time variable | N mean sd p50 min max p25 p75

---------------+--------------------------------------------------------------------------

0 ICAMTn | 46 1178.812 949.4425 856.7862 102.742 4252.149 615.74 1476.743

ln_ICAM | 46 6.794959 .768743 6.753117 4.632221 8.35518 6.422825 7.297594

---------------+--------------------------------------------------------------------------

3 ICAMTn | 44 1004.609 948.183 633.5316 182.9275 4518.37 440.8116 1063.963

ln_ICAM | 44 6.595921 .7771678 6.451308 5.20909 8.415907 6.086327 6.969323

---------------+--------------------------------------------------------------------------

6 ICAMTn | 42 1401.286 1381.567 1150.884 286.6437 8571.6 637.23 1617.49

ln_ICAM | 42 6.981108 .6862845 7.047968 5.65824 9.05621 6.45713 7.388631

---------------+--------------------------------------------------------------------------

24 ICAMTn | 45 1190.826 1614.08 690.71 93.58 6490.38 326.71 986.99

ln_ICAM | 45 6.501526 1.032698 6.53772 4.538816 8.778076 5.789073 6.89466

---------------+--------------------------------------------------------------------------

Total ICAMTn | 177 1191.352 1251.149 786.18 93.58 8571.6 509.854 1446.36

ln_ICAM | 177 6.71505 .8418824 6.667186 4.538816 9.05621 6.234124 7.276805

. mixed ln_ICAM i.time##restricted ||RecordID:, mle

Computing standard errors:

Mixed-effects ML regression Number of obs = 343

Group variable: RecordID Number of groups = 95

Obs per group:

min = 1

avg = 3.6

max = 4

Wald chi2(7) = 23.02

Log likelihood = -378.38054 Prob > chi2 = 0.0017

---------------------------------------------------------------------------------

ln_ICAM | Coef. Std. Err. z P>|z| [95% Conf. Interval]

----------------+----------------------------------------------------------------

time |

3 | -.1001755 .1254824 -0.80 0.425 -.3461164 .1457655

6 | .137375 .1306466 1.05 0.293 -.1186876 .3934375

24 | -.0449901 .1312499 -0.34 0.732 -.3022353 .212255

|

restricted |

Restricted | .1991152 .1768771 1.13 0.260 -.1475575 .545788

|

time#restricted |

3#Restricted | -.0931678 .1771581 -0.53 0.599 -.4403914 .2540557

6#Restricted | .0727286 .1818358 0.40 0.689 -.283663 .4291201

24#Restricted | -.2584969 .1806792 -1.43 0.153 -.6126216 .0956279

|

_cons | 6.587981 .1266304 52.03 0.000 6.33979 6.836172

---------------------------------------------------------------------------------

------------------------------------------------------------------------------

Random-effects Parameters | Estimate Std. Err. [95% Conf. Interval]

-----------------------------+------------------------------------------------

RecordID: Identity |

var(_cons) | .3718956 .0683527 .2594025 .5331726

-----------------------------+------------------------------------------------

var(Residual) | .3440511 .0307812 .2887145 .4099939

------------------------------------------------------------------------------

LR test vs. linear model: chibar2(01) = 105.27 Prob >= chibar2 = 0.0000

. testparm time#restricted

( 1) [ln_ICAM]3.time#1.restricted = 0

( 2) [ln_ICAM]6.time#1.restricted = 0

( 3) [ln_ICAM]24.time#1.restricted = 0

chi2( 3) = 3.59

Prob > chi2 = 0.3097

Adjusting for pscore:

. mixed ln_ICAM i.time##restricted pscore ||RecordID:, mle

Mixed-effects ML regression Number of obs = 327

Group variable: RecordID Number of groups = 90

Obs per group:

min = 1

avg = 3.6

max = 4

Wald chi2(8) = 22.50

Log likelihood = -365.09217 Prob > chi2 = 0.0041

---------------------------------------------------------------------------------

ln_ICAM | Coef. Std. Err. z P>|z| [95% Conf. Interval]

----------------+----------------------------------------------------------------

time |

3 | -.0870803 .1294171 -0.67 0.501 -.3407331 .1665724

6 | .1538689 .1348885 1.14 0.254 -.1105077 .4182454

24 | -.0330583 .1359924 -0.24 0.808 -.2995985 .2334818

|

restricted |

Restricted | .1636988 .1851728 0.88 0.377 -.1992331 .5266308

|

time#restricted |

3#Restricted | -.0825705 .1848967 -0.45 0.655 -.4449614 .2798205

6#Restricted | .0559489 .1891499 0.30 0.767 -.314778 .4266759

24#Restricted | -.2738079 .1888623 -1.45 0.147 -.6439712 .0963554

|

pscore | .8588279 .6605046 1.30 0.194 -.4357373 2.153393

_cons | 6.155644 .345607 17.81 0.000 5.478267 6.833021

---------------------------------------------------------------------------------

------------------------------------------------------------------------------

Random-effects Parameters | Estimate Std. Err. [95% Conf. Interval]

-----------------------------+------------------------------------------------

RecordID: Identity |

var(_cons) | .3669073 .0699655 .252488 .5331775

-----------------------------+------------------------------------------------

var(Residual) | .3576094 .0327443 .298861 .4279062

------------------------------------------------------------------------------

LR test vs. linear model: chibar2(01) = 95.29 Prob >= chibar2 = 0.0000

. testparm time#restricted

( 1) [ln_ICAM]3.time#1.restricted = 0

( 2) [ln_ICAM]6.time#1.restricted = 0

( 3) [ln_ICAM]24.time#1.restricted = 0

chi2( 3) = 3.34

Prob > chi2 = 0.3415

The pscore variable is not significantly associated with ln_ICAM so the loss of sample results in a slightly higher p value.

. mixed ln_ICAM i.time i.restricted ||RecordID:, mle

Mixed-effects ML regression Number of obs = 343

Group variable: RecordID Number of groups = 95

Obs per group:

min = 1

avg = 3.6

max = 4

Wald chi2(4) = 19.15

Log likelihood = -380.15847 Prob > chi2 = 0.0007

------------------------------------------------------------------------------

ln_ICAM | Coef. Std. Err. z P>|z| [95% Conf. Interval]

-------------+----------------------------------------------------------------

time |

3 | -.14717 .0892575 -1.65 0.099 -.3221114 .0277715

6 | .1745773 .0915644 1.91 0.057 -.0048855 .3540402

24 | -.1820933 .0908721 -2.00 0.045 -.3601994 -.0039872

|

restricted |

Restricted | .1277744 .1409048 0.91 0.365 -.148394 .4039428

_cons | 6.624573 .1143979 57.91 0.000 6.400357 6.848789

------------------------------------------------------------------------------

------------------------------------------------------------------------------

Random-effects Parameters | Estimate Std. Err. [95% Conf. Interval]

-----------------------------+------------------------------------------------

RecordID: Identity |

var(_cons) | .3689333 .0681035 .2569329 .5297562

-----------------------------+------------------------------------------------

var(Residual) | .3494156 .031252 .2932311 .4163653

------------------------------------------------------------------------------

LR test vs. linear model: chibar2(01) = 103.05 Prob >= chibar2 = 0.0000

. mixed ln_ICAM i.time i.restricted pscore ||RecordID:, mle

Mixed-effects ML regression Number of obs = 327

Group variable: RecordID Number of groups = 90

Obs per group:

min = 1

avg = 3.6

max = 4

Wald chi2(5) = 18.92

Log likelihood = -366.75088 Prob > chi2 = 0.0020

------------------------------------------------------------------------------

ln_ICAM | Coef. Std. Err. z P>|z| [95% Conf. Interval]

-------------+----------------------------------------------------------------

time |

3 | -.1279111 .0931215 -1.37 0.170 -.310426 .0546038

6 | .182579 .0952454 1.92 0.055 -.0040985 .3692566

24 | -.1760773 .0950424 -1.85 0.064 -.362357 .0102024

|

restricted |

Restricted | .0882885 .1481932 0.60 0.551 -.2021647 .3787418

pscore | .8805294 .6593852 1.34 0.182 -.4118418 2.172901

_cons | 6.181789 .3403784 18.16 0.000 5.51466 6.848919

------------------------------------------------------------------------------

------------------------------------------------------------------------------

Random-effects Parameters | Estimate Std. Err. [95% Conf. Interval]

-----------------------------+------------------------------------------------

RecordID: Identity |

var(_cons) | .3641153 .0697614 .2501243 .5300562

-----------------------------+------------------------------------------------

var(Residual) | .3630137 .0332337 .3033864 .4343601

------------------------------------------------------------------------------

LR test vs. linear model: chibar2(01) = 93.26 Prob >= chibar2 = 0.0000

# VCAM

. bysort restricted: tabstat VCAMTn ln_VCAM, ///

> s(n mean sd median min max p25 p75) by(time) col(stats) long

-> restricted = Standard

time variable | N mean sd p50 min max p25 p75

---------------+--------------------------------------------------------------------------

0 VCAMTn | 44 3347.944 2509.362 3075.428 153.732 11527.32 1210.772 4844.592

ln_VCAM | 44 7.81113 .8657168 8.030795 5.035211 9.352475 7.098776 8.485601

---------------+--------------------------------------------------------------------------

3 VCAMTn | 44 2781.526 2905.384 1745.262 499.92 15414.4 923.2938 3826.935

ln_VCAM | 44 7.528852 .8828268 7.464597 6.214448 9.643058 6.827929 8.249764

---------------+--------------------------------------------------------------------------

6 VCAMTn | 39 3913.901 3936.816 2235.21 512.36 16201.56 1680.84 4765.01

ln_VCAM | 39 7.898227 .8426952 7.71209 6.239028 9.692863 7.427049 8.469055

---------------+--------------------------------------------------------------------------

24 VCAMTn | 39 2664.442 3158.358 1354.57 161.31 13573.82 920.25 3618.52

ln_VCAM | 39 7.37727 1.002769 7.211239 5.083328 9.515898 6.824646 8.19382

---------------+--------------------------------------------------------------------------

Total VCAMTn | 166 3170.193 3153.955 1924.69 153.732 16201.56 1052.342 3902.76

ln_VCAM | 166 7.654841 .9146104 7.56252 5.035211 9.692863 6.958774 8.26944

------------------------------------------------------------------------------------------

-> restricted = Restricted

time variable | N mean sd p50 min max p25 p75

---------------+--------------------------------------------------------------------------

0 VCAMTn | 46 3751.263 2712.992 3066.33 98.276 12039.82 1858.77 4780.522

ln_VCAM | 46 7.920961 .9259488 8.028208 4.58778 9.395975 7.52767 8.472305

---------------+--------------------------------------------------------------------------

3 VCAMTn | 44 2750.948 2153.723 2006.753 419.89 8683.32 1071.023 4196.398

ln_VCAM | 44 7.593754 .8567596 7.603785 6.039993 9.06916 6.975978 8.338664

---------------+--------------------------------------------------------------------------

6 VCAMTn | 42 4580.95 3361.392 3549.085 774.68 13344.67 2345.6 5830.51

ln_VCAM | 42 8.167645 .7542785 8.174376 6.65245 9.498873 7.760296 8.670859

---------------+--------------------------------------------------------------------------

24 VCAMTn | 45 2785.146 4209.453 1176.2 196.76 20071.1 570.77 2718.82

ln_VCAM | 45 7.220264 1.158012 7.070044 5.281985 9.907036 6.346986 7.907953

---------------+--------------------------------------------------------------------------

Total VCAMTn | 177 3453.849 3262.103 2513.45 98.276 20071.1 1173.055 4406.68

ln_VCAM | 177 7.720013 .9962689 7.829412 4.58778 9.907036 7.067367 8.390877

------------------------------------------------------------------------------------------

. mixed ln_VCAM i.time##restricted ||RecordID:, mle

Mixed-effects ML regression Number of obs = 343

Group variable: RecordID Number of groups = 95

Obs per group:

min = 1

avg = 3.6

max = 4

Wald chi2(7) = 52.73

Log likelihood = -432.53206 Prob > chi2 = 0.0000

---------------------------------------------------------------------------------

ln_VCAM | Coef. Std. Err. z P>|z| [95% Conf. Interval]

----------------+----------------------------------------------------------------

time |

3 | -.2955892 .1578944 -1.87 0.061 -.6050565 .0138781

6 | .0768408 .164039 0.47 0.639 -.2446696 .3983513

24 | -.4197595 .164567 -2.55 0.011 -.742305 -.0972141

|

restricted |

Restricted | .1172638 .1913034 0.61 0.540 -.257684 .4922116

|

time#restricted |

3#Restricted | -.0215603 .2227027 -0.10 0.923 -.4580496 .4149289

6#Restricted | .1713787 .228373 0.75 0.453 -.2762241 .6189814

24#Restricted | -.2900036 .2267322 -1.28 0.201 -.7343905 .1543832

|

_cons | 7.798731 .136852 56.99 0.000 7.530506 8.066956

---------------------------------------------------------------------------------

------------------------------------------------------------------------------

Random-effects Parameters | Estimate Std. Err. [95% Conf. Interval]

-----------------------------+------------------------------------------------

RecordID: Identity |

var(_cons) | .2843482 .0660217 .1803904 .4482162

-----------------------------+------------------------------------------------

var(Residual) | .5456084 .0490977 .4573872 .6508458

------------------------------------------------------------------------------

LR test vs. linear model: chibar2(01) = 42.87 Prob >= chibar2 = 0.0000

. testparm time#restricted

( 1) [ln_VCAM]3.time#1.restricted = 0

( 2) [ln_VCAM]6.time#1.restricted = 0

( 3) [ln_VCAM]24.time#1.restricted = 0

chi2( 3) = 4.01

Prob > chi2 = 0.2600

mixed ln_VCAM i.time##restricted pscore ||RecordID:, mle

Mixed-effects ML regression Number of obs = 327

Group variable: RecordID Number of groups = 90

Obs per group:

min = 1

avg = 3.6

max = 4

Wald chi2(8) = 46.23

Log likelihood = -416.51382 Prob > chi2 = 0.0000

---------------------------------------------------------------------------------

ln_VCAM | Coef. Std. Err. z P>|z| [95% Conf. Interval]

----------------+----------------------------------------------------------------

time |

3 | -.2845518 .1617097 -1.76 0.078 -.601497 .0323934

6 | .0912699 .1682192 0.54 0.587 -.2384336 .4209735

24 | -.4017802 .169552 -2.37 0.018 -.7340961 -.0694643

|

restricted |

Restricted | .0628982 .2009508 0.31 0.754 -.3309582 .4567546

|

time#restricted |

3#Restricted | .001406 .2308001 0.01 0.995 -.450954 .4537659

6#Restricted | .1504044 .2359687 0.64 0.524 -.3120858 .6128946

24#Restricted | -.2824244 .2355139 -1.20 0.230 -.7440231 .1791743

|

pscore | .5972615 .6482307 0.92 0.357 -.6732473 1.86777

_cons | 7.516705 .3445581 21.82 0.000 6.841383 8.192026

---------------------------------------------------------------------------------

------------------------------------------------------------------------------

Random-effects Parameters | Estimate Std. Err. [95% Conf. Interval]

-----------------------------+------------------------------------------------

RecordID: Identity |

var(_cons) | .2933609 .0694935 .184401 .4667036

-----------------------------+------------------------------------------------

var(Residual) | .5591697 .0514751 .4668584 .6697335

------------------------------------------------------------------------------

LR test vs. linear model: chibar2(01) = 41.42 Prob >= chibar2 = 0.0000

. testparm time#restricted

( 1) [ln_VCAM]3.time#1.restricted = 0

( 2) [ln_VCAM]6.time#1.restricted = 0

( 3) [ln_VCAM]24.time#1.restricted = 0

chi2( 3) = 3.36

Prob > chi2 = 0.3398

mixed ln_VCAM i.time i.restricted ||RecordID:, mle

Mixed-effects ML regression Number of obs = 343

Group variable: RecordID Number of groups = 95

Obs per group:

min = 1

avg = 3.6

max = 4

Wald chi2(4) = 47.89

Log likelihood = -434.51831 Prob > chi2 = 0.0000

------------------------------------------------------------------------------

ln_VCAM | Coef. Std. Err. z P>|z| [95% Conf. Interval]

-------------+----------------------------------------------------------------

time |

3 | -.3066574 .1123392 -2.73 0.006 -.5268382 -.0864765

6 | .165074 .1151387 1.43 0.152 -.0605936 .3907417

24 | -.5745775 .1141827 -5.03 0.000 -.7983716 -.3507835

|

restricted |

Restricted | .0797195 .1360687 0.59 0.558 -.1869702 .3464092

_cons | 7.818175 .1186189 65.91 0.000 7.585687 8.050664

------------------------------------------------------------------------------

------------------------------------------------------------------------------

Random-effects Parameters | Estimate Std. Err. [95% Conf. Interval]

-----------------------------+------------------------------------------------

RecordID: Identity |

var(_cons) | .2792584 .0655524 .1762777 .4423999

-----------------------------+------------------------------------------------

var(Residual) | .555501 .049943 .4657539 .6625416

------------------------------------------------------------------------------

LR test vs. linear model: chibar2(01) = 41.21 Prob >= chibar2 = 0.0000

. mixed ln_VCAM i.time i.restricted pscore ||RecordID:, mle

Mixed-effects ML regression Number of obs = 327

Group variable: RecordID Number of groups = 90

Obs per group:

min = 1

avg = 3.6

max = 4

Wald chi2(5) = 42.25

Log likelihood = -418.17759 Prob > chi2 = 0.0000

------------------------------------------------------------------------------

ln_VCAM | Coef. Std. Err. z P>|z| [95% Conf. Interval]

-------------+----------------------------------------------------------------

time |

3 | -.2841591 .1162783 -2.44 0.015 -.5120604 -.0562579

6 | .1680934 .1188455 1.41 0.157 -.0648394 .4010263

24 | -.5507236 .1185498 -4.65 0.000 -.783077 -.3183702

|

restricted |

Restricted | .0289759 .145366 0.20 0.842 -.2559363 .3138881

pscore | .6251786 .6465483 0.97 0.334 -.6420327 1.89239

_cons | 7.519105 .3364043 22.35 0.000 6.859764 8.178445

------------------------------------------------------------------------------

------------------------------------------------------------------------------

Random-effects Parameters | Estimate Std. Err. [95% Conf. Interval]

-----------------------------+------------------------------------------------

RecordID: Identity |

var(_cons) | .289049 .069146 .1808623 .46195

-----------------------------+------------------------------------------------

var(Residual) | .5679423 .0522547 .4742285 .6801752

------------------------------------------------------------------------------

LR test vs. linear model: chibar2(01) = 40.02 Prob >= chibar2 = 0.0000

# E-selectin

.

. bysort restricted: tabstat EselectinTn ln_Eselectin, ///

> s(n mean sd median min max p25 p75) by(time) col(stats) long

-> restricted = Standard

time variable | N mean sd p50 min max p25 p75

-------------------+--------------------------------------------------------------------------

0 EselectinTn | 44 49481.31 47033.67 29660.28 4276.98 173431.8 11582.83 87468.3

ln_Eselectin | 44 10.32436 1.04826 10.29756 8.361002 12.06354 9.35493 11.36694

-------------------+--------------------------------------------------------------------------

3 EselectinTn | 43 43662.84 38380.57 30932.33 3079.69 152063.3 16393.73 59561.01

ln_Eselectin | 43 10.27208 .9854581 10.33956 8.032584 11.93205 9.704655 10.99476

-------------------+--------------------------------------------------------------------------

6 EselectinTn | 39 46997.56 40430.18 33938.34 6761.25 151415.8 12013.12 74509.13

ln_Eselectin | 39 10.35186 .9538203 10.4323 8.818963 11.92778 9.393755 11.21868

-------------------+--------------------------------------------------------------------------

24 EselectinTn | 39 36726.23 27223.25 29876.71 5602.95 119418.1 14952.59 57357.36

ln_Eselectin | 39 10.22579 .7979845 10.30483 8.631048 11.69039 9.612639 10.95706

-------------------+--------------------------------------------------------------------------

Total EselectinTn | 165 44363.07 39120.49 29876.71 3079.69 173431.8 14075.05 65621.7

ln_Eselectin | 165 10.29394 .947333 10.30483 8.032584 12.06354 9.552158 11.09166

----------------------------------------------------------------------------------------------

-> restricted = Restricted

time variable | N mean sd p50 min max p25 p75

-------------------+--------------------------------------------------------------------------

0 EselectinTn | 47 61821.28 71638.2 32285.01 3347.52 317128.1 13527.9 104306.2

ln_Eselectin | 47 10.43025 1.147073 10.38236 8.115975 12.66706 9.512509 11.55509

-------------------+--------------------------------------------------------------------------

3 EselectinTn | 41 55268.2 60757.3 31425.51 4473.5 308372.4 11821.12 89099.6

ln_Eselectin | 41 10.37495 1.088818 10.35538 8.405927 12.63906 9.377643 11.39751

-------------------+--------------------------------------------------------------------------

6 EselectinTn | 42 55525.41 55017.82 26599.1 3966.48 185488.6 9587.07 111197.2

ln_Eselectin | 42 10.34026 1.161242 10.18623 8.285634 12.13075 9.168171 11.61906

-------------------+--------------------------------------------------------------------------

24 EselectinTn | 45 45026.49 48116.99 22668.49 4660.06 219418.5 11330.45 66169.96

ln_Eselectin | 45 10.20163 1.034232 10.02873 8.446784 12.29874 9.335249 11.09998

-------------------+--------------------------------------------------------------------------

Total EselectinTn | 175 54456.32 59515.93 29886.93 3347.52 317128.1 12043.41 86600.8

ln_Eselectin | 175 10.33691 1.102743 10.30518 8.115975 12.66706 9.396273 11.36906

----------------------------------------------------------------------------------------------

. mixed ln_Eselectin i.time##restricted ||RecordID:, mle

Mixed-effects ML regression Number of obs = 340

Group variable: RecordID Number of groups = 95

Obs per group:

min = 1

avg = 3.6

max = 4

Wald chi2(7) = 13.18

Log likelihood = -303.38448 Prob > chi2 = 0.0679

---------------------------------------------------------------------------------

ln_Eselectin | Coef. Std. Err. z P>|z| [95% Conf. Interval]

----------------+----------------------------------------------------------------

time |

3 | -.0690907 .0824213 -0.84 0.402 -.2306335 .092452

6 | .0247303 .0853205 0.29 0.772 -.1424949 .1919555

24 | -.1601723 .0859313 -1.86 0.062 -.3285946 .0082499

|

restricted |

Restricted | .1372985 .213327 0.64 0.520 -.2808147 .5554118

|

time#restricted |

3#Restricted | -.044828 .1171618 -0.38 0.702 -.274461 .184805

6#Restricted | -.1334087 .1184324 -1.13 0.260 -.3655319 .0987145

24#Restricted | -.0609726 .1174685 -0.52 0.604 -.2912067 .1692615

|

_cons | 10.30292 .1532581 67.23 0.000 10.00254 10.6033

---------------------------------------------------------------------------------

------------------------------------------------------------------------------

Random-effects Parameters | Estimate Std. Err. [95% Conf. Interval]

-----------------------------+------------------------------------------------

RecordID: Identity |

var(_cons) | .9250291 .1408371 .6863705 1.246672

-----------------------------+------------------------------------------------

var(Residual) | .1449483 .0131042 .1214114 .1730481

------------------------------------------------------------------------------

LR test vs. linear model: chibar2(01) = 374.80 Prob >= chibar2 = 0.0000

. testparm time#restricted

( 1) [ln_Eselectin]3.time#1.restricted = 0

( 2) [ln_Eselectin]6.time#1.restricted = 0

( 3) [ln_Eselectin]24.time#1.restricted = 0

chi2( 3) = 1.30

Prob > chi2 = 0.7284

. mixed ln_Eselectin i.time##restricted pscore ||RecordID:, mle

Mixed-effects ML regression Number of obs = 324

Group variable: RecordID Number of groups = 90

Obs per group:

min = 1

avg = 3.6

max = 4

Wald chi2(8) = 16.26

Log likelihood = -284.91416 Prob > chi2 = 0.0387

---------------------------------------------------------------------------------

ln_Eselectin | Coef. Std. Err. z P>|z| [95% Conf. Interval]

----------------+----------------------------------------------------------------

time |

3 | -.0609735 .0839642 -0.73 0.468 -.2255404 .1035934

6 | .0346955 .0869915 0.40 0.690 -.1358047 .2051957

24 | -.1447819 .0877199 -1.65 0.099 -.3167097 .027146

|

restricted |

Restricted | .0697398 .2124817 0.33 0.743 -.3467166 .4861962

|

time#restricted |

3#Restricted | -.0442014 .1209165 -0.37 0.715 -.2811934 .1927906

6#Restricted | -.1291039 .1216252 -1.06 0.288 -.3674849 .1092771

24#Restricted | -.0617858 .1210755 -0.51 0.610 -.2990893 .1755178

|

pscore | 2.032377 .8930012 2.28 0.023 .2821271 3.782627

_cons | 9.289028 .45791 20.29 0.000 8.391541 10.18652

---------------------------------------------------------------------------------

------------------------------------------------------------------------------

Random-effects Parameters | Estimate Std. Err. [95% Conf. Interval]

-----------------------------+------------------------------------------------

RecordID: Identity |

var(_cons) | .8150258 .1283948 .5985185 1.109852

-----------------------------+------------------------------------------------

var(Residual) | .146942 .0135991 .1225658 .1761662

------------------------------------------------------------------------------

LR test vs. linear model: chibar2(01) = 330.81 Prob >= chibar2 = 0.0000

. testparm time#restricted

( 1) [ln_Eselectin]3.time#1.restricted = 0

( 2) [ln_Eselectin]6.time#1.restricted = 0

( 3) [ln_Eselectin]24.time#1.restricted = 0

chi2( 3) = 1.16

Prob > chi2 = 0.7631

. mixed ln_Eselectin i.time i.restricted ||RecordID:, mle

Mixed-effects ML regression Number of obs = 340

Group variable: RecordID Number of groups = 95

Obs per group:

min = 1

avg = 3.6

max = 4

Wald chi2(4) = 11.81

Log likelihood = -304.03427 Prob > chi2 = 0.0188

------------------------------------------------------------------------------

ln_Eselectin | Coef. Std. Err. z P>|z| [95% Conf. Interval]

-------------+----------------------------------------------------------------

time |

3 | -.0927794 .0587033 -1.58 0.114 -.2078357 .0222769

6 | -.0445627 .059322 -0.75 0.453 -.1608317 .0717063

24 | -.1926185 .0587317 -3.28 0.001 -.3077306 -.0775064

|

restricted |

Restricted | .0806598 .2020999 0.40 0.690 -.3154487 .4767683

_cons | 10.3322 .1491664 69.27 0.000 10.03984 10.62457

------------------------------------------------------------------------------

------------------------------------------------------------------------------

Random-effects Parameters | Estimate Std. Err. [95% Conf. Interval]

-----------------------------+------------------------------------------------

RecordID: Identity |

var(_cons) | .9250619 .1408786 .6863419 1.246812

-----------------------------+------------------------------------------------

var(Residual) | .145704 .0131726 .1220442 .1739506

------------------------------------------------------------------------------

LR test vs. linear model: chibar2(01) = 373.80 Prob >= chibar2 = 0.0000

. mixed ln_Eselectin i.time i.restricted pscore ||RecordID:, mle

Mixed-effects ML regression Number of obs = 324

Group variable: RecordID Number of groups = 90

Obs per group:

min = 1

avg = 3.6

max = 4

Wald chi2(5) = 15.06

Log likelihood = -285.49177 Prob > chi2 = 0.0101

------------------------------------------------------------------------------

ln_Eselectin | Coef. Std. Err. z P>|z| [95% Conf. Interval]

-------------+----------------------------------------------------------------

time |

3 | -.0838571 .0605372 -1.39 0.166 -.2025078 .0347936

6 | -.0314428 .0609387 -0.52 0.606 -.1508805 .0879949

24 | -.1770178 .0605994 -2.92 0.003 -.2957903 -.0582453

|

restricted |

Restricted | .0143954 .2006195 0.07 0.943 -.3788116 .4076023

pscore | 2.023833 .8929753 2.27 0.023 .2736336 3.774032

_cons | 9.321384 .4565046 20.42 0.000 8.426651 10.21612

------------------------------------------------------------------------------

------------------------------------------------------------------------------

Random-effects Parameters | Estimate Std. Err. [95% Conf. Interval]

-----------------------------+------------------------------------------------

RecordID: Identity |

var(_cons) | .8149116 .1284117 .5983844 1.10979

-----------------------------+------------------------------------------------

var(Residual) | .1476627 .0136659 .1231668 .1770304

------------------------------------------------------------------------------

LR test vs. linear model: chibar2(01) = 329.95 Prob >= chibar2 = 0.0000

# NGAL (censored values)

N=2 values below assay limit at T0, both in standard group.

*Note: Mean should not be reported for variables with censored data.*

. bysort restricted:tabstat NGALTn ln_NGAL, ///

> s(n mean sd median min max p25 p75) by(time) col(stats) long

-> restricted = Standard

time variable | N mean sd p50 min max p25 p75

---------------+-------------------------------------------------------------------------

0 NGALTn | 44 500.8092 630.7681 286.35 .078 3392 137.5 517.1

ln_NGAL | 44 5.402982 1.990467 5.656284 -2.551046 8.129175 4.923624 6.248203

---------------+-------------------------------------------------------------------------

3 NGALTn | 44 395.2427 462.4407 262 53.05 2763 155 468.75

ln_NGAL | 44 5.574103 .8871835 5.567187 3.971235 7.924072 5.04249 6.149761

---------------+-------------------------------------------------------------------------

6 NGALTn | 38 358.9292 279.3044 283 86.14 1551 145.4 493.3

ln_NGAL | 38 5.635105 .7187144 5.645433 4.455974 7.346655 4.979488 6.201118

---------------+-------------------------------------------------------------------------

24 NGALTn | 38 369.3279 366.4599 229.8 71.77 1689.7 174.4 378.72

ln_NGAL | 38 5.603435 .7382187 5.437203 4.273467 7.432306 5.161352 5.936797

---------------+-------------------------------------------------------------------------

Total NGALTn |164 409.1467 461.3495 261.3 .078 3392 161.2 486.8

ln_NGAL |164 5.549123 1.22561 5.56566 -2.551046 8.129175 5.082641 6.187767

-----------------------------------------------------------------------------------------

-> restricted = Restricted

time variable | N mean sd p50 min max p25 p75

---------------+-------------------------------------------------------------------------

0 NGALTn | 46 538.5878 641.2056 354.5 70.9 3756 158.3 661.3

ln_NGAL | 46 5.824384 .9545971 5.864041 4.261271 8.23111 5.064492 6.494207

---------------+-------------------------------------------------------------------------

3 NGALTn | 43 397.0626 405.0819 241.6 52.28 1848 136.8 517.3

ln_NGAL | 43 5.56615 .924491 5.487283 3.956614 7.521859 4.91852 6.248623

---------------+-------------------------------------------------------------------------

6 NGALTn | 42 614.8912 816.9829 341.25 37.36 3605 148 594

ln_NGAL | 42 5.809241 1.101595 5.832448 3.620601 8.190077 4.997212 6.386879

---------------+-------------------------------------------------------------------------

24 NGALTn | 45 431.5949 447.3749 281.7 41.56 2021 151.1 540.8

ln_NGAL | 45 5.645558 .9360525 5.640842 3.727138 7.611348 5.017942 6.293049

---------------+-------------------------------------------------------------------------

Total NGALTn |176 494.8632 599.2855 310.1 37.36 3756 147.48 568.7

ln_NGAL |176 5.711956 .9777561 5.736827 3.620601 8.23111 4.993686 6.343353

-----------------------------------------------------------------------------------------

. xttobit ln_NGAL i.time##restricted, ll(ln(0.0781))

Random-effects tobit regression Number of obs = 340

Uncensored = 338

Limits: lower = ln(0.0781) Left-censored = 2

upper = +inf Right-censored = 0

Group variable: RecordID Number of groups = 95

Random effects u_i ~ Gaussian Obs per group:

min = 1

avg = 3.6

max = 4

Integration method: mvaghermite Integration pts. = 12

Wald chi2(7) = 8.33

Log likelihood = -419.71872 Prob > chi2 = 0.3047

---------------------------------------------------------------------------------

ln_NGAL | Coef. Std. Err. z P>|z| [95% Conf. Interval]

----------------+----------------------------------------------------------------

time |

3 | .1734417 .1277492 1.36 0.175 -.0769421 .4238254

6 | .2070696 .1344327 1.54 0.123 -.0564136 .4705529

24 | .1342677 .1352973 0.99 0.321 -.1309102 .3994456

|

restricted |

Restricted | .4502126 .2352029 1.91 0.056 -.0107766 .9112017

|

time#restricted |

3#Restricted | -.3744592 .1812039 -2.07 0.039 -.7296122 -.0193061

6#Restricted | -.2508776 .1862499 -1.35 0.178 -.6159207 .1141655

24#Restricted | -.354018 .1853009 -1.91 0.056 -.717201 .0091651

|

_cons | 5.378484 .1685951 31.90 0.000 5.048043 5.708924

----------------+----------------------------------------------------------------

/sigma_u | .9627218 .0789969 12.19 0.000 .8078907 1.117553

/sigma_e | .5966346 .0272483 21.90 0.000 .5432288 .6500404

----------------+----------------------------------------------------------------

rho | .7225046 .0388471 .6417431 .7931712

---------------------------------------------------------------------------------

LR test of sigma_u=0: chibar2(01) = 195.58 Prob >= chibar2 = 0.000

. testparm time#restricted

( 1) [ln_NGAL]3.time#1.restricted = 0

( 2) [ln_NGAL]6.time#1.restricted = 0

( 3) [ln_NGAL]24.time#1.restricted = 0

chi2( 3) = 5.38

Prob > chi2 = 0.1457

xttobit ln_NGAL i.time##restricted pscore, ll(ln(0.0781)) nolog

Random-effects tobit regression Number of obs = 324

Uncensored = 322

Limits: lower = ln(0.0781) Left-censored = 2

upper = +inf Right-censored = 0

Group variable: RecordID Number of groups = 90

Random effects u_i ~ Gaussian Obs per group:

min = 1

avg = 3.6

max = 4

Integration method: mvaghermite Integration pts. = 12

Wald chi2(8) = 18.44

Log likelihood = -400.8179 Prob > chi2 = 0.0182

---------------------------------------------------------------------------------

ln_NGAL | Coef. Std. Err. z P>|z| [95% Conf. Interval]

----------------+----------------------------------------------------------------

time |

3 | .1934373 .1319432 1.47 0.143 -.0651667 .4520412

6 | .2260516 .1390354 1.63 0.104 -.0464527 .4985559

24 | .1637626 .1402556 1.17 0.243 -.1111335 .4386586

|

restricted |

Restricted | .3238603 .2388527 1.36 0.175 -.1442824 .792003

|

time#restricted |

3#Restricted | -.3821207 .1894696 -2.02 0.044 -.7534743 -.010767

6#Restricted | -.2613865 .1940035 -1.35 0.178 -.6416263 .1188533

24#Restricted | -.3850452 .1938437 -1.99 0.047 -.7649718 -.0051186

|

pscore | 2.972433 .9374034 3.17 0.002 1.135156 4.80971

_cons | 3.940047 .4843748 8.13 0.000 2.99069 4.889404

----------------+----------------------------------------------------------------

/sigma_u | .9150779 .0787021 11.63 0.000 .7608246 1.069331

/sigma_e | .6091564 .0285321 21.35 0.000 .5532345 .6650782

----------------+----------------------------------------------------------------

rho | .6929332 .0432029 .6037878 .7719247

---------------------------------------------------------------------------------

LR test of sigma_u=0: chibar2(01) = 162.51 Prob >= chibar2 = 0.000

. testparm time#restricted

( 1) [ln_NGAL]3.time#1.restricted = 0

( 2) [ln_NGAL]6.time#1.restricted = 0

( 3) [ln_NGAL]24.time#1.restricted = 0

chi2( 3) = 5.44

Prob > chi2 = 0.1426

. xttobit ln_NGAL i.time i.restricted , ll(ln(0.0781)) nolog

Random-effects tobit regression Number of obs = 340

Uncensored = 338

Limits: lower = ln(0.0781) Left-censored = 2

upper = +inf Right-censored = 0

Group variable: RecordID Number of groups = 95

Random effects u_i ~ Gaussian Obs per group:

min = 1

avg = 3.6

max = 4

Integration method: mvaghermite Integration pts. = 12

Wald chi2(4) = 2.90

Log likelihood = -422.38402 Prob > chi2 = 0.5748

------------------------------------------------------------------------------

ln_NGAL | Coef. Std. Err. z P>|z| [95% Conf. Interval]

-------------+----------------------------------------------------------------

time |

3 | -.0139641 .0915463 -0.15 0.879 -.1933915 .1654633

6 | .0781749 .094022 0.83 0.406 -.1061048 .2624547

24 | -.0509527 .0933971 -0.55 0.585 -.2340076 .1321022

|

restricted |

Restricted | .2095277 .2088702 1.00 0.316 -.1998503 .6189057

_cons | 5.501199 .1595579 34.48 0.000 5.188472 5.813927

-------------+----------------------------------------------------------------

/sigma_u | .9623692 .0791617 12.16 0.000 .8072151 1.117523

/sigma_e | .602961 .0275385 21.90 0.000 .5489865 .6569355

-------------+----------------------------------------------------------------

rho | .7181071 .0393164 .636476 .7897055

------------------------------------------------------------------------------

LR test of sigma_u=0: chibar2(01) = 192.32 Prob >= chibar2 = 0.000

. xttobit ln_NGAL i.time i.restricted pscore, ll(ln(0.0781)) nolog

Random-effects tobit regression Number of obs = 324

Uncensored = 322

Limits: lower = ln(0.0781) Left-censored = 2

upper = +inf Right-censored = 0

Group variable: RecordID Number of groups = 90

Random effects u_i ~ Gaussian Obs per group:

min = 1

avg = 3.6

max = 4

Integration method: mvaghermite Integration pts. = 12

Wald chi2(5) = 12.94

Log likelihood = -403.50811 Prob > chi2 = 0.0240

------------------------------------------------------------------------------

ln_NGAL | Coef. Std. Err. z P>|z| [95% Conf. Interval]

-------------+----------------------------------------------------------------

time |

3 | .0066167 .0957182 0.07 0.945 -.1809875 .1942209

6 | .0946223 .0980132 0.97 0.334 -.0974801 .2867246

24 | -.0340309 .0978596 -0.35 0.728 -.2258322 .1577704

|

restricted |

Restricted | .0725452 .2109187 0.34 0.731 -.3408478 .4859382

pscore | 2.974341 .9384853 3.17 0.002 1.134944 4.813739

_cons | 4.063787 .4814391 8.44 0.000 3.120184 5.00739

-------------+----------------------------------------------------------------

/sigma_u | .9151915 .0789524 11.59 0.000 .7604476 1.069935

/sigma_e | .6158543 .0288505 21.35 0.000 .5593084 .6724002

-------------+----------------------------------------------------------------

rho | .6883133 .0436935 .5982829 .7682955

------------------------------------------------------------------------------

LR test of sigma_u=0: chibar2(01) = 159.47 Prob >= chibar2 = 0.000

# Resistin (Censored values)

N=5 below assay limit , N=5 above limit:

T0: n=2 below

T3: n=1 below n=4 above

T6: n=1 below n=1 above

T24: n=1 below

. bysort restricted:tabstat ResistinTn ln_Resistin, ///

> s(n mean sd median min max p25 p75) by(time) col(stats) long

-> restricted = Standard

time variable | N mean sd p50 min max p25 p75

------------------+-------------------------------------------------------------------------

0 ResistinTn | 44 139.663 135.5117 103.5 .00312 658.2 54.785 145.75

ln_Resistin | 44 4.379256 1.777039 4.639437 -5.769922 6.489509 4.003383 4.981426

------------------+-------------------------------------------------------------------------

3 ResistinTn | 44 197.4673 326.6554 117.7 .00312 1600 59.63 164.4

ln_Resistin | 44 4.520946 1.844421 4.768126 -5.769922 7.377759 4.08759 5.102205

------------------+-------------------------------------------------------------------------

6 ResistinTn | 39 136.9273 142.2846 100.8 .00312 729.3 55.65 151.5

ln_Resistin | 39 4.358997 1.832793 4.613138 -5.769922 6.592085 4.019082 5.020586

------------------+-------------------------------------------------------------------------

24 ResistinTn | 39 128.0419 157.5135 73.72 .00312 810.5 35.01 162.3

ln_Resistin | 39 4.161445 1.867763 4.300274 -5.769922 6.697651 3.555634 5.089447

------------------+-------------------------------------------------------------------------

Total ResistinTn |166 151.6116 209.1668 109.6 .00312 1600 48.68 156.5

ln_Resistin |166 4.36088 1.817381 4.696827 -5.769922 7.377759 3.885268 5.053056

--------------------------------------------------------------------------------------------

-> restricted = Restricted

time variable | N mean sd p50 min max p25 p75

------------------+--------------------------------------------------------------------------

0 ResistinTn | 46 121.5222 204.9924 72.635 .00312 1350 30.81 122.1

ln_Resistin | 46 4.05401 1.774747 4.285203 -5.769922 7.20786 3.427839 4.804841

------------------+--------------------------------------------------------------------------

3 ResistinTn | 44 177.4314 323.9039 104.155 16.81 1600 41.39 171.35

ln_Resistin | 44 4.518049 1.049493 4.641484 2.821974 7.377759 3.722912 5.143606

------------------+--------------------------------------------------------------------------

6 ResistinTn | 41 153.9963 253.1218 106.9 10.82 1600 38.12 188.6

ln_Resistin | 41 4.440625 1.07022 4.671894 2.381396 7.377759 3.640739 5.239628

------------------+--------------------------------------------------------------------------

24 ResistinTn | 45 132.92 165.4365 90.1 13.68 811.9 36.87 139.2

ln_Resistin | 45 4.337727 1.067772 4.50092 2.615935 6.699377 3.607398 4.935912

------------------+--------------------------------------------------------------------------

Total ResistinTn | 176 145.9787 242.0236 89.965 .00312 1600 37.755 159.95

ln_Resistin | 176 4.332625 1.288728 4.49942 -5.769922 7.377759 3.631097 5.074753

---------------------------------------------------------------------------------------------

. xttobit ln_Resistin i.time##restricted, ll(ln(0.00312001)) ul(ln(1600)) nolog

Random-effects tobit regression Number of obs = 342

Uncensored = 332

Limits: lower = ln(0.00312001) Left-censored = 5

upper = ln(1600) Right-censored = 5

Group variable: RecordID Number of groups = 95

Random effects u_i ~ Gaussian Obs per group:

min = 1

avg = 3.6

max = 4

Integration method: mvaghermite Integration pts. = 12

Wald chi2(7) = 9.97

Log likelihood = -567.26442 Prob > chi2 = 0.1901

---------------------------------------------------------------------------------

ln_Resistin | Coef. Std. Err. z P>|z| [95% Conf. Interval]

----------------+----------------------------------------------------------------

time |

3 | .1654624 .2097154 0.79 0.430 -.2455723 .576497

6 | .0011784 .2179301 0.01 0.996 -.4259567 .4283135

24 | -.2142402 .2191066 -0.98 0.328 -.6436813 .215201

|

restricted |

Restricted | -.300447 .3275638 -0.92 0.359 -.9424602 .3415661

|

time#restricted |

3#Restricted | .3730013 .2963007 1.26 0.208 -.2077373 .95374

6#Restricted | .3611925 .3047738 1.19 0.236 -.2361531 .9585381

24#Restricted | .45535 .3014643 1.51 0.131 -.1355092 1.046209

|

_cons | 4.362111 .2346277 18.59 0.000 3.90225 4.821973

----------------+----------------------------------------------------------------

/sigma_u | 1.230998 .105155 11.71 0.000 1.024898 1.437098

/sigma_e | .976713 .0446167 21.89 0.000 .889266 1.06416

----------------+----------------------------------------------------------------

rho | .6136722 .0471377 .5189232 .7020671

---------------------------------------------------------------------------------

LR test of sigma_u=0: chibar2(01) = 155.75 Prob >= chibar2 = 0.000

. testparm time#restricted

( 1) [ln_Resistin]3.time#1.restricted = 0

( 2) [ln_Resistin]6.time#1.restricted = 0

( 3) [ln_Resistin]24.time#1.restricted = 0

chi2( 3) = 2.78

Prob > chi2 = 0.4266

. xttobit ln_Resistin i.time##restricted pscore, ll(ln(0.00312001)) ul(ln(1600)) nolog

Random-effects tobit regression Number of obs = 326

Uncensored = 316

Limits: lower = ln(0.00312001) Left-censored = 5

upper = ln(1600) Right-censored = 5

Group variable: RecordID Number of groups = 90

Random effects u_i ~ Gaussian Obs per group:

min = 1

avg = 3.6

max = 4

Integration method: mvaghermite Integration pts. = 12

Wald chi2(8) = 26.35

Log likelihood = -539.77389 Prob > chi2 = 0.0009

---------------------------------------------------------------------------------

ln_Resistin | Coef. Std. Err. z P>|z| [95% Conf. Interval]

----------------+----------------------------------------------------------------

time |

3 | .173081 .2169705 0.80 0.425 -.2521735 .5983354

6 | .0205957 .2256184 0.09 0.927 -.4216083 .4627996

24 | -.1910173 .2274852 -0.84 0.401 -.6368801 .2548455

|

restricted |

Restricted | -.5417737 .329061 -1.65 0.100 -1.186721 .103174

|

time#restricted |

3#Restricted | .4052449 .3102487 1.31 0.191 -.2028314 1.013321

6#Restricted | .3723234 .3179891 1.17 0.242 -.2509237 .9955706

24#Restricted | .4703051 .3158636 1.49 0.137 -.1487761 1.089386

|

pscore | 4.998482 1.21246 4.12 0.000 2.622105 7.37486

_cons | 1.933961 .6315686 3.06 0.002 .6961095 3.171813

----------------+----------------------------------------------------------------

/sigma_u | 1.136221 .1031073 11.02 0.000 .9341339 1.338307

/sigma_e | .9988826 .0467576 21.36 0.000 .9072393 1.090526

----------------+----------------------------------------------------------------

rho | .5640588 .0518475 .4614475 .6625101

---------------------------------------------------------------------------------

LR test of sigma_u=0: chibar2(01) = 122.44 Prob >= chibar2 = 0.000

. testparm time#restricted

( 1) [ln_Resistin]3.time#1.restricted = 0

( 2) [ln_Resistin]6.time#1.restricted = 0

( 3) [ln_Resistin]24.time#1.restricted = 0

chi2( 3) = 2.78

Prob > chi2 = 0.4263

. xttobit ln_Resistin i.time i.restricted , ll(ln(0.00312001)) ul(ln(1600)) nolog

Random-effects tobit regression Number of obs = 342

Uncensored = 332

Limits: lower = ln(0.00312001) Left-censored = 5

upper = ln(1600) Right-censored = 5

Group variable: RecordID Number of groups = 95

Random effects u_i ~ Gaussian Obs per group:

min = 1

avg = 3.6

max = 4

Integration method: mvaghermite Integration pts. = 12

Wald chi2(4) = 7.12

Log likelihood = -568.64832 Prob > chi2 = 0.1296

------------------------------------------------------------------------------

ln_Resistin | Coef. Std. Err. z P>|z| [95% Conf. Interval]

-------------+----------------------------------------------------------------

time |

3 | .3540304 .148938 2.38 0.017 .0621174 .6459435

6 | .1870043 .1531436 1.22 0.222 -.1131516 .4871602

24 | .0242665 .1512516 0.16 0.873 -.2721811 .3207141

|

restricted |

Restricted | -.0096261 .2754938 -0.03 0.972 -.549584 .5303318

_cons | 4.21364 .2167733 19.44 0.000 3.788772 4.638508

-------------+----------------------------------------------------------------

/sigma_u | 1.230828 .1053168 11.69 0.000 1.024411 1.437246

/sigma_e | .9819602 .0448574 21.89 0.000 .8940413 1.069879

-------------+----------------------------------------------------------------

rho | .6110633 .0473307 .5160021 .6998819

------------------------------------------------------------------------------

LR test of sigma_u=0: chibar2(01) = 154.26 Prob >= chibar2 = 0.000

. xttobit ln_Resistin i.time i.restricted pscore, ll(ln(0.00312001)) ul(ln(1600)) nolog

Random-effects tobit regression Number of obs = 326

Uncensored = 316

Limits: lower = ln(0.00312001) Left-censored = 5

upper = ln(1600) Right-censored = 5

Group variable: RecordID Number of groups = 90

Random effects u_i ~ Gaussian Obs per group:

min = 1

avg = 3.6

max = 4

Integration method: mvaghermite Integration pts. = 12

Wald chi2(5) = 23.50

Log likelihood = -541.15799 Prob > chi2 = 0.0003

------------------------------------------------------------------------------

ln_Resistin | Coef. Std. Err. z P>|z| [95% Conf. Interval]

-------------+----------------------------------------------------------------

time |

3 | .3731838 .1559786 2.39 0.017 .0674713 .6788963

6 | .2083369 .1598762 1.30 0.193 -.1050147 .5216885

24 | .0503625 .1586724 0.32 0.751 -.2606298 .3613548

|

restricted |

Restricted | -.2379565 .2723003 -0.87 0.382 -.7716552 .2957422

pscore | 5.001238 1.212692 4.12 0.000 2.624406 7.378071

_cons | 1.781458 .6247085 2.85 0.004 .5570517 3.005864

-------------+----------------------------------------------------------------

/sigma_u | 1.13542 .1032542 11.00 0.000 .9330451 1.337794

/sigma_e | 1.004613 .0470251 21.36 0.000 .9124455 1.09678

-------------+----------------------------------------------------------------

rho | .5608962 .0520348 .4580215 .6597947

------------------------------------------------------------------------------

LR test of sigma_u=0: chibar2(01) = 121.01 Prob >= chibar2 = 0.000

# Syn-1

T0 n=1 below, T24 n=1 below. (T3 & T6 no censoring)

.

. bysort restricted:tabstat Syn1Tn ln_Syn1, ///

> s(n mean sd median min max p25 p75) by(time) col(stats) long

-> restricted = Standard

time variable | N mean sd p50 min max p25 p75

---------------+--------------------------------------------------------------------------

0 Syn1Tn | 44 19054.61 26707.77 7018.5 125 112800 5017 20245

ln_Syn1 | 44 9.175513 1.218563 8.856192 4.828314 11.63337 8.520562 9.91318

---------------+--------------------------------------------------------------------------

3 Syn1Tn | 44 19299.05 32357.55 6900 2030 160200 3895.5 18395

ln_Syn1 | 44 9.141363 1.071065 8.835694 7.615791 11.98418 8.267572 9.819808

---------------+--------------------------------------------------------------------------

6 Syn1Tn | 38 17021.03 23192.46 5939.5 2400 83260 3940 17340

ln_Syn1 | 38 9.110046 1.051717 8.689157 7.783224 11.32972 8.278936 9.760771

---------------+--------------------------------------------------------------------------

24 Syn1Tn | 39 17889.23 28017.61 7681 125 141000 5060 12960

ln_Syn1 | 39 9.094888 1.197965 8.946505 4.828314 11.85651 8.529121 9.469623

---------------+--------------------------------------------------------------------------

Total Syn1Tn | 165 18376 27669.85 7124 125 160200 4608 17910

ln_Syn1 | 165 9.132272 1.128346 8.871224 4.828314 11.98418 8.435549 9.793115

------------------------------------------------------------------------------------------

-> restricted = Restricted

time variable | N mean sd p50 min max p25 p75

---------------+--------------------------------------------------------------------------

0 Syn1Tn | 46 19543.37 36457.98 5494 1305 171100 3255 16870

ln_Syn1 | 46 8.965577 1.232086 8.611003 7.173958 12.05 8.087948 9.733293

---------------+--------------------------------------------------------------------------

3 Syn1Tn | 44 20635.23 37833.84 5925 1709 154400 2880.5 15105

ln_Syn1 | 44 8.951675 1.248398 8.686905 7.443664 11.9473 7.965696 9.622244

---------------+--------------------------------------------------------------------------

6 Syn1Tn | 41 18760.15 41320.19 5073 1592 186300 2861 10880

ln_Syn1 | 41 8.831703 1.184667 8.531688 7.372746 12.13511 7.958927 9.294682

---------------+--------------------------------------------------------------------------

24 Syn1Tn | 44 25044.61 46833.7 6962 1360 226800 4772.5 15520

ln_Syn1 | 44 9.192314 1.223774 8.848127 7.21524 12.33182 8.470367 9.649257

---------------+--------------------------------------------------------------------------

Total Syn1Tn | 175 21017.57 40471.55 5837 1305 226800 3422 14870

ln_Syn1 | 175 8.987725 1.219529 8.671972 7.173958 12.33182 8.13798 9.607101

------------------------------------------------------------------------------------------

. xttobit ln_Syn1 i.time##restricted, ll(ln(125.0001)) nolog

Random-effects tobit regression Number of obs = 340

Uncensored = 338

Limits: lower = ln(125.0001) Left-censored = 2

upper = +inf Right-censored = 0

Group variable: RecordID Number of groups = 95

Random effects u_i ~ Gaussian Obs per group:

min = 1

avg = 3.6

max = 4

Integration method: mvaghermite Integration pts. = 12

Wald chi2(7) = 5.52

Log likelihood = -342.23397 Prob > chi2 = 0.5962

---------------------------------------------------------------------------------

ln_Syn1 | Coef. Std. Err. z P>|z| [95% Conf. Interval]

----------------+----------------------------------------------------------------

time |

3 | -.0427184 .0897929 -0.48 0.634 -.2187093 .1332725

6 | -.0972075 .0946073 -1.03 0.304 -.2826344 .0882194

24 | .0077195 .0944911 0.08 0.935 -.1774796 .1929186

|

restricted |

Restricted | -.1340735 .2433862 -0.55 0.582 -.6111017 .3429547

|

time#restricted |

3#Restricted | -.0149329 .126928 -0.12 0.906 -.2637072 .2338413

6#Restricted | .0082777 .1315707 0.06 0.950 -.2495961 .2661515

24#Restricted | .0718855 .1303772 0.55 0.581 -.1836491 .32742

|

_cons | 9.145298 .1746372 52.37 0.000 8.803016 9.487581

----------------+----------------------------------------------------------------

/sigma_u | 1.101937 .083456 13.20 0.000 .9383661 1.265508

/sigma_e | .4191371 .0190632 21.99 0.000 .3817738 .4565003

----------------+----------------------------------------------------------------

rho | .8736091 .019721 .830791 .9082312

---------------------------------------------------------------------------------

LR test of sigma_u=0: chibar2(01) = 391.32 Prob >= chibar2 = 0.000

. testparm time#restricted

( 1) [ln_Syn1]3.time#1.restricted = 0

( 2) [ln_Syn1]6.time#1.restricted = 0

( 3) [ln_Syn1]24.time#1.restricted = 0

chi2( 3) = 0.50

Prob > chi2 = 0.9192

. xttobit ln_Syn1 i.time##restricted pscore, ll(ln(125.0001)) nolog

Random-effects tobit regression Number of obs = 324

Uncensored = 322

Limits: lower = ln(125.0001) Left-censored = 2

upper = +inf Right-censored = 0

Group variable: RecordID Number of groups = 90

Random effects u_i ~ Gaussian Obs per group:

min = 1

avg = 3.6

max = 4

Integration method: mvaghermite Integration pts. = 12

Wald chi2(8) = 7.74

Log likelihood = -331.07315 Prob > chi2 = 0.4597

---------------------------------------------------------------------------------

ln_Syn1 | Coef. Std. Err. z P>|z| [95% Conf. Interval]

----------------+----------------------------------------------------------------

time |

3 | -.049522 .0928173 -0.53 0.594 -.2314406 .1323965

6 | -.0956901 .0979259 -0.98 0.328 -.2876213 .096241

24 | .0095064 .0978918 0.10 0.923 -.182358 .2013708

|

restricted |

Restricted | -.0871016 .2568975 -0.34 0.735 -.5906114 .4164083

|

time#restricted |

3#Restricted | -.0057646 .1328017 -0.04 0.965 -.2660511 .2545219

6#Restricted | .0125784 .1371945 0.09 0.927 -.2563178 .2814747

24#Restricted | .0823968 .1364894 0.60 0.546 -.1851175 .3499112

|

pscore | -1.653662 1.088336 -1.52 0.129 -3.786762 .4794376

_cons | 9.976467 .5575282 17.89 0.000 8.883732 11.0692

----------------+----------------------------------------------------------------

/sigma_u | 1.104429 .086009 12.84 0.000 .9358548 1.273004

/sigma_e | .4282524 .0199359 21.48 0.000 .3891787 .4673261

----------------+----------------------------------------------------------------

rho | .8692953 .0208436 .8239883 .9058225

---------------------------------------------------------------------------------

LR test of sigma_u=0: chibar2(01) = 366.89 Prob >= chibar2 = 0.000

. testparm time#restricted

( 1) [ln_Syn1]3.time#1.restricted = 0

( 2) [ln_Syn1]6.time#1.restricted = 0

( 3) [ln_Syn1]24.time#1.restricted = 0

chi2( 3) = 0.52

Prob > chi2 = 0.9155

. xttobit ln_Syn1 i.time i.restricted , ll(ln(125.0001)) nolog

Random-effects tobit regression Number of obs = 340

Uncensored = 338

Limits: lower = ln(125.0001) Left-censored = 2

upper = +inf Right-censored = 0

Group variable: RecordID Number of groups = 95

Random effects u_i ~ Gaussian Obs per group:

min = 1

avg = 3.6

max = 4

Integration method: mvaghermite Integration pts. = 12

Wald chi2(4) = 5.02

Log likelihood = -342.48329 Prob > chi2 = 0.2853

------------------------------------------------------------------------------

ln_Syn1 | Coef. Std. Err. z P>|z| [95% Conf. Interval]

-------------+----------------------------------------------------------------

time |

3 | -.0498497 .0635012 -0.79 0.432 -.1743098 .0746104

6 | -.0924106 .0657806 -1.40 0.160 -.2213382 .036517

24 | .0459991 .0651212 0.71 0.480 -.0816362 .1736344

|

restricted |

Restricted | -.1181105 .2313269 -0.51 0.610 -.5715028 .3352818

_cons | 9.137007 .170398 53.62 0.000 8.803033 9.47098

-------------+----------------------------------------------------------------

/sigma_u | 1.102977 .0835143 13.21 0.000 .9392918 1.266662

/sigma_e | .4193977 .0190732 21.99 0.000 .3820149 .4567806

-------------+----------------------------------------------------------------

rho | .8736801 .0197049 .8308973 .9082746

------------------------------------------------------------------------------

LR test of sigma_u=0: chibar2(01) = 392.09 Prob >= chibar2 = 0.000

. xttobit ln_Syn1 i.time i.restricted pscore, ll(ln(125.0001)) nolog

Random-effects tobit regression Number of obs = 324

Uncensored = 322

Limits: lower = ln(125.0001) Left-censored = 2

upper = +inf Right-censored = 0

Group variable: RecordID Number of groups = 90

Random effects u_i ~ Gaussian Obs per group:

min = 1

avg = 3.6

max = 4

Integration method: mvaghermite Integration pts. = 12

Wald chi2(5) = 7.21

Log likelihood = -331.33071 Prob > chi2 = 0.2056

------------------------------------------------------------------------------

ln_Syn1 | Coef. Std. Err. z P>|z| [95% Conf. Interval]

-------------+----------------------------------------------------------------

time |

3 | -.0519461 .0664289 -0.78 0.434 -.1821444 .0782523

6 | -.0888503 .0686241 -1.29 0.195 -.223351 .0456505

24 | .052373 .0682442 0.77 0.443 -.0813833 .1861292

|

restricted |

Restricted | -.0658418 .244579 -0.27 0.788 -.5452078 .4135241

pscore | -1.653675 1.089248 -1.52 0.129 -3.788563 .4812125

_cons | 9.96604 .5564613 17.91 0.000 8.875396 11.05668

-------------+----------------------------------------------------------------

/sigma_u | 1.10543 .0860714 12.84 0.000 .9367328 1.274126

/sigma_e | .4285621 .0199489 21.48 0.000 .389463 .4676612

-------------+----------------------------------------------------------------

rho | .8693368 .0208328 .8240537 .9058456

------------------------------------------------------------------------------

LR test of sigma_u=0: chibar2(01) = 367.46 Prob >= chibar2 = 0.000

# Hyaluronan (censored values)

N=19 below limit:

T0: n=3 below

T3: n=4 below

T6: n=5 below

T24: n=7 below

. bysort restricted:tabstat HyaluronanTn ln_Hyaluronan, ///

> s(n mean sd median min max p25 p75) by(time) col(stats) long

-> restricted = Standard

time variable | N mean sd p50 min max p25 p75

-------------------+--------------------------------------------------------------------------

0 HyaluronanTn | 44 919.6863 1688.167 216.35 19.29 8705 73.01 1123.5

ln_Hyaluro~n | 44 5.589233 1.622557 5.368176 2.959587 9.071652 4.290078 7.024187

-------------------+--------------------------------------------------------------------------

3 HyaluronanTn | 44 930.0122 2700.382 144.05 17.487 13765 80.1 543.4

ln_Hyaluro~n | 44 5.330779 1.484715 4.968478 2.861458 9.529884 4.383259 6.297845

-------------------+--------------------------------------------------------------------------

6 HyaluronanTn | 39 671.8738 2095.658 136.26 22.194 12760 57.828 372.06

ln_Hyaluro~n | 39 5.113658 1.399687 4.914565 3.099822 9.45407 4.057473 5.919055

-------------------+--------------------------------------------------------------------------

24 HyaluronanTn | 39 1397.426 6422.33 124.5 .37 40120 35.556 230.34

ln_Hyaluro~n | 39 4.64359 2.247867 4.824306 -.9942523 10.59963 3.571109 5.439557

-------------------+--------------------------------------------------------------------------

Total HyaluronanTn | 166 976.4422 3635.781 139.74 .37 40120 66.66 507.9

ln_Hyaluro~n | 166 5.186827 1.730661 4.939766 -.9942523 10.59963 4.199605 6.230285

----------------------------------------------------------------------------------------------

-> restricted = Restricted

time variable | N mean sd p50 min max p25 p75

------------------+-------------------------------------------------------------------------

0 HyaluronanTn | 45 1046.664 2709.155 261 .37 17312 74.54 702

ln_Hyaluro~n | 45 5.269859 2.267081 5.56452 -.9942523 9.759155 4.311336 6.553934

------------------+-------------------------------------------------------------------------

3 HyaluronanTn | 44 508.0078 1614.518 151.85 .37 10725 40.54 377.1

ln_Hyaluro~n | 44 4.593765 2.217609 5.021752 -.9942523 9.280333 3.70148 5.93249

------------------+-------------------------------------------------------------------------

6 HyaluronanTn | 42 560.9594 2249.22 120.6 .37 14676 32.67 269.94

ln_Hyaluro~n | 42 4.322726 2.351174 4.792145 -.9942523 9.593968 3.486457 5.5982

------------------+-------------------------------------------------------------------------

24 HyaluronanTn | 45 978.4124 3217.148 172.5 .37 18485 42.762 515.16

ln_Hyaluro~n | 45 4.811197 2.374468 5.150397 -.9942523 9.824715 3.75565 6.244478

------------------+-------------------------------------------------------------------------

TotalHyaluronanTn |176 778.6424 2516.332 159.26 .37 18485 45.513 456.3

ln_Hyaluro~n |176 4.757544 2.309479 5.070538 -.9942523 9.824715 3.817935 6.123134

--------------------------------------------------------------------------------------------

. xttobit ln_Hyaluronan i.time##restricted, ll(ln(0.370001)) nolog

Random-effects tobit regression Number of obs = 342

Uncensored = 323

Limits: lower = ln(0.370001) Left-censored = 19

upper = +inf Right-censored = 0

Group variable: RecordID Number of groups = 95

Random effects u_i ~ Gaussian Obs per group:

min = 1

avg = 3.6

max = 4

Integration method: mvaghermite Integration pts. = 12

Wald chi2(7) = 28.75

Log likelihood = -636.98091 Prob > chi2 = 0.0002

---------------------------------------------------------------------------------

ln_Hyaluronan | Coef. Std. Err. z P>|z| [95% Conf. Interval]

----------------+----------------------------------------------------------------

time |

3 | -.2537176 .2498721 -1.02 0.310 -.7434578 .2360227

6 | -.375499 .2605987 -1.44 0.150 -.886263 .135265

24 | -.8948789 .2626093 -3.41 0.001 -1.409584 -.3801741

|

restricted |

Restricted | -.2813145 .4433277 -0.63 0.526 -1.150221 .5875917

|

time#restricted |

3#Restricted | -.4252979 .3554824 -1.20 0.232 -1.122031 .2714348

6#Restricted | -.5976325 .3655434 -1.63 0.102 -1.314084 .1188193

24#Restricted | .3672709 .3631548 1.01 0.312 -.3444996 1.079041

|

_cons | 5.481823 .3168923 17.30 0.000 4.860725 6.10292

----------------+----------------------------------------------------------------

/sigma_u | 1.777107 .1490687 11.92 0.000 1.484938 2.069276

/sigma_e | 1.167295 .0551237 21.18 0.000 1.059255 1.275336

----------------+----------------------------------------------------------------

rho | .6985904 .041494 .6129213 .7745035

---------------------------------------------------------------------------------

LR test of sigma_u=0: chibar2(01) = 189.92 Prob >= chibar2 = 0.000

. testparm time#restricted

( 1) [ln_Hyaluronan]3.time#1.restricted = 0

( 2) [ln_Hyaluronan]6.time#1.restricted = 0

( 3) [ln_Hyaluronan]24.time#1.restricted = 0

chi2( 3) = 8.20

Prob > chi2 = 0.0421

Although none of the pairwise comparisons displayed in the output for the interaction term are significant, the test above indicates that there is a comparison that is significant. Examination of the box plots (as well as the coefficients in the output above) suggests that T24 will be involved.

The model below changes the reference group for time to T24 so that comparisons now show T3 vs T24 and T6 vs T24 that were not visible previously.

. xttobit ln_Hyaluronan b24.time##restricted, ll(ln(0.370001)) nolog

Random-effects tobit regression Number of obs = 342

Uncensored = 323

Limits: lower = ln(0.370001) Left-censored = 19

upper = +inf Right-censored = 0

Group variable: RecordID Number of groups = 95

Random effects u_i ~ Gaussian Obs per group:

min = 1

avg = 3.6

max = 4

Integration method: mvaghermite Integration pts. = 12

Wald chi2(7) = 28.75

Log likelihood = -636.98091 Prob > chi2 = 0.0002

---------------------------------------------------------------------------------

ln_Hyaluronan | Coef. Std. Err. z P>|z| [95% Conf. Interval]

----------------+----------------------------------------------------------------

time |

0 | .8948789 .2626093 3.41 0.001 .3801741 1.409584

3 | .6411613 .2628237 2.44 0.015 .1260363 1.156286

6 | .5193799 .2697547 1.93 0.054 -.0093295 1.048089

|

restricted |

Restricted | .0859563 .4492669 0.19 0.848 -.7945907 .9665033

|

time#restricted |

0#Restricted | -.3672709 .3631548 -1.01 0.312 -1.079041 .3444996

3#Restricted | -.7925688 .3651755 -2.17 0.030 -1.5083 -.0768379

6#Restricted | -.9649034 .374218 -2.58 0.010 -1.698357 -.2314497

|

_cons | 4.586944 .3252557 14.10 0.000 3.949454 5.224433

----------------+----------------------------------------------------------------

/sigma_u | 1.777107 .1490687 11.92 0.000 1.484938 2.069276

/sigma_e | 1.167295 .0551237 21.18 0.000 1.059255 1.275336

----------------+----------------------------------------------------------------

rho | .6985904 .041494 .6129213 .7745035

---------------------------------------------------------------------------------

LR test of sigma_u=0: chibar2(01) = 189.92 Prob >= chibar2 = 0.000

The interaction term is indicating that the change between T3 and T24 is significantly different between restricted and standard groups. Similarly for T6 and T24.

Bootstrapped:

. xttobit ln_Hyaluronan b24.time##restricted, ll(ln(0.370001)) nolog ///

vce(bootstrap, reps(500) seed(010967))

Random-effects tobit regression Number of obs = 342

Uncensored = 323

Limits: lower = ln(0.370001) Left-censored = 19

upper = +inf Right-censored = 0

Group variable: RecordID Number of groups = 95

Random effects u_i ~ Gaussian Obs per group:

min = 1

avg = 3.6

max = 4

Integration method: mvaghermite Integration pts. = 12

Wald chi2(7) = 24.01

Log likelihood = -636.98091 Prob > chi2 = 0.0011

(Replications based on 95 clusters in RecordID)

---------------------------------------------------------------------------------

| Observed Bootstrap Normal-based

ln_Hyaluronan | Coef. Std. Err. z P>|z| [95% Conf. Interval]

----------------+----------------------------------------------------------------

time |

0 | .8948789 .3037506 2.95 0.003 .2995386 1.490219

3 | .6411613 .2158635 2.97 0.003 .2180766 1.064246

6 | .5193799 .2294968 2.26 0.024 .0695745 .9691853

|

restricted |

Restricted | .0859563 .5226112 0.16 0.869 -.9383428 1.110255

|

time#restricted |

0#Restricted | -.3672709 .4567732 -0.80 0.421 -1.26253 .5279882

3#Restricted | -.7925688 .3849623 -2.06 0.040 -1.547081 -.0380565

6#Restricted | -.9649034 .3615892 -2.67 0.008 -1.673605 -.2562015

|

_cons | 4.586944 .3634193 12.62 0.000 3.874655 5.299232

----------------+----------------------------------------------------------------

/sigma_u | 1.777107 .1808455 9.83 0.000 1.422656 2.131558

/sigma_e | 1.167295 .1275208 9.15 0.000 .9173593 1.417232

----------------+----------------------------------------------------------------

rho | .6985904 .0472753 .6004145 .7841487

---------------------------------------------------------------------------------

LR test of sigma_u=0: chibar2(01) = 189.92 Prob >= chibar2 = 0.000

. testparm time#restricted

( 1) [ln_Hyaluronan]0.time#1.restricted = 0

( 2) [ln_Hyaluronan]3.time#1.restricted = 0

( 3) [ln_Hyaluronan]6.time#1.restricted = 0

chi2( 3) = 7.67

Prob > chi2 = 0.0534

If the model is bootstrapped, the p value for the overall test is nudged over 0.05.

Restricting sample to those with complete data in the propensity score:

. xttobit ln_Hyaluronan b24.time##restricted if pscore~=., ll(ln(0.370001)) nolog

Random-effects tobit regression Number of obs = 326

Uncensored = 311

Limits: lower = ln(0.370001) Left-censored = 15

upper = +inf Right-censored = 0

Group variable: RecordID Number of groups = 90

Random effects u_i ~ Gaussian Obs per group:

min = 1

avg = 3.6

max = 4

Integration method: mvaghermite Integration pts. = 12

Wald chi2(7) = 29.78

Log likelihood = -598.87965 Prob > chi2 = 0.0001

---------------------------------------------------------------------------------

ln_Hyaluronan | Coef. Std. Err. z P>|z| [95% Conf. Interval]

----------------+----------------------------------------------------------------

time |

0 | .8613097 .2606402 3.30 0.001 .3504643 1.372155

3 | .600811 .2608541 2.30 0.021 .0895464 1.112076

6 | .482237 .267941 1.80 0.072 -.0429176 1.007392

|

restricted |

Restricted | -.0822547 .4387505 -0.19 0.851 -.94219 .7776805

|

time#restricted |

0#Restricted | -.1830209 .3639208 -0.50 0.615 -.8962927 .5302508

3#Restricted | -.6477429 .3659982 -1.77 0.077 -1.365086 .0696003

6#Restricted | -.8070643 .3737884 -2.16 0.031 -1.539676 -.0744525

|

_cons | 4.725516 .316255 14.94 0.000 4.105668 5.345365

----------------+----------------------------------------------------------------

/sigma_u | 1.661298 .1415337 11.74 0.000 1.383897 1.938699

/sigma_e | 1.142024 .0545775 20.92 0.000 1.035054 1.248994

----------------+----------------------------------------------------------------

rho | .6790903 .0434603 .5898835 .7589932

---------------------------------------------------------------------------------

LR test of sigma_u=0: chibar2(01) = 176.95 Prob >= chibar2 = 0.000

. testparm time#restricted

( 1) [ln_Hyaluronan]0.time#1.restricted = 0

( 2) [ln_Hyaluronan]3.time#1.restricted = 0

( 3) [ln_Hyaluronan]6.time#1.restricted = 0

chi2( 3) = 6.34

Prob > chi2 = 0.0962

The change in sample alone is responsible for the change in p value from the original model. The inclusion of the propensity score below has almost no impact on this p value.

Now adding the pscore:

. xttobit ln_Hyaluronan b24.time##restricted pscore, ll(ln(0.370001)) nolog

Random-effects tobit regression Number of obs = 326

Uncensored = 311

Limits: lower = ln(0.370001) Left-censored = 15

upper = +inf Right-censored = 0

Group variable: RecordID Number of groups = 90

Random effects u_i ~ Gaussian Obs per group:

min = 1

avg = 3.6

max = 4

Integration method: mvaghermite Integration pts. = 12

Wald chi2(8) = 32.55

Log likelihood = -597.55127 Prob > chi2 = 0.0001

---------------------------------------------------------------------------------

ln_Hyaluronan | Coef. Std. Err. z P>|z| [95% Conf. Interval]

----------------+----------------------------------------------------------------

time |

0 | .8624847 .2605078 3.31 0.001 .3518988 1.373071

3 | .6043435 .2607279 2.32 0.020 .0933262 1.115361

6 | .4898862 .2678441 1.83 0.067 -.0350786 1.014851

|

restricted |

Restricted | -.2185837 .4420217 -0.49 0.621 -1.08493 .6477628

|

time#restricted |

0#Restricted | -.1856741 .3637194 -0.51 0.610 -.8985511 .5272028

3#Restricted | -.6486078 .365778 -1.77 0.076 -1.36552 .0683039

6#Restricted | -.8170466 .3736048 -2.19 0.029 -1.549299 -.0847947

|

pscore | 2.764251 1.683769 1.64 0.101 -.535876 6.064378

_cons | 3.383105 .8756645 3.86 0.000 1.666834 5.099376

----------------+----------------------------------------------------------------

/sigma_u | 1.633982 .1396804 11.70 0.000 1.360213 1.90775

/sigma_e | 1.141517 .0545179 20.94 0.000 1.034664 1.24837

----------------+----------------------------------------------------------------

rho | .6720173 .0440717 .5817541 .7532011

---------------------------------------------------------------------------------

LR test of sigma_u=0: chibar2(01) = 173.70 Prob >= chibar2 = 0.000

. testparm time#restricted

( 1) [ln_Hyaluronan]0.time#1.restricted = 0

( 2) [ln_Hyaluronan]3.time#1.restricted = 0

( 3) [ln_Hyaluronan]6.time#1.restricted = 0

chi2( 3) = 6.45

Prob > chi2 = 0.0917

These models are the same as the previous two models but using T0 as the reference again.

. xttobit ln_Hyaluronan i.time i.restricted , ll(ln(0.370001)) nolog

Random-effects tobit regression Number of obs = 342

Uncensored = 323

Limits: lower = ln(0.370001) Left-censored = 19

upper = +inf Right-censored = 0

Group variable: RecordID Number of groups = 95

Random effects u_i ~ Gaussian Obs per group:

min = 1

avg = 3.6

max = 4

Integration method: mvaghermite Integration pts. = 12

Wald chi2(4) = 20.01

Log likelihood = -641.02551 Prob > chi2 = 0.0005

-------------------------------------------------------------------------------

ln_Hyaluronan | Coef. Std. Err. z P>|z| [95% Conf. Interval]

--------------+----------------------------------------------------------------

time |

3 | -.4635922 .1805043 -2.57 0.010 -.8173741 -.1098102

6 | -.676322 .1855509 -3.64 0.000 -1.039995 -.3126489

24 | -.6930357 .1841853 -3.76 0.000 -1.054032 -.3320391

|

restricted |

Restricted | -.436898 .3890434 -1.12 0.261 -1.199409 .325613

_cons | 5.558701 .2994921 18.56 0.000 4.971708 6.145695

--------------+----------------------------------------------------------------

/sigma_u | 1.779711 .1499837 11.87 0.000 1.485749 2.073674

/sigma_e | 1.185679 .0560143 21.17 0.000 1.075893 1.295465

--------------+----------------------------------------------------------------

rho | .6925934 .0421565 .6057077 .769828

-------------------------------------------------------------------------------

LR test of sigma_u=0: chibar2(01) = 184.96 Prob >= chibar2 = 0.000

. xttobit ln_Hyaluronan i.time i.restricted pscore, ll(ln(0.370001)) nolog

Random-effects tobit regression Number of obs = 326

Uncensored = 311

Limits: lower = ln(0.370001) Left-censored = 15

upper = +inf Right-censored = 0

Group variable: RecordID Number of groups = 90

Random effects u_i ~ Gaussian Obs per group:

min = 1

avg = 3.6

max = 4

Integration method: mvaghermite Integration pts. = 12

Wald chi2(5) = 25.53

Log likelihood = -600.73348 Prob > chi2 = 0.0001

-------------------------------------------------------------------------------

ln_Hyaluronan | Coef. Std. Err. z P>|z| [95% Conf. Interval]

--------------+----------------------------------------------------------------

time |

3 | -.4819093 .1801554 -2.67 0.007 -.8350074 -.1288112

6 | -.6873677 .1845769 -3.72 0.000 -1.049132 -.3256037

24 | -.7571549 .1842784 -4.11 0.000 -1.118334 -.3959759

|

restricted |

Restricted | -.6234139 .3784733 -1.65 0.100 -1.365208 .1183802

pscore | 2.707701 1.684618 1.61 0.108 -.5940898 6.009493

_cons | 4.382377 .8650991 5.07 0.000 2.686814 6.07794

--------------+----------------------------------------------------------------

/sigma_u | 1.632077 .140077 11.65 0.000 1.357532 1.906623

/sigma_e | 1.157362 .0552668 20.94 0.000 1.049041 1.265683

--------------+----------------------------------------------------------------

rho | .6653929 .0446846 .5740592 .7478488

-------------------------------------------------------------------------------

LR test of sigma_u=0: chibar2(01) = 169.46 Prob >= chibar2 = 0.000

# IL6 (Censored values)

N=8 below limit

T0 n=1 below, T3 n=1 below, T24 n=6 below

. bysort restricted:tabstat Il6Tn ln_Il6, ///

> s(n mean sd median min max p25 p75) by(time) col(stats) long

-> restricted = Standard

time variable | N mean sd p50 min max p25 p75

----------------+-------------------------------------------------------------------------

0 Il6Tn | 44 58797.99 164142.2 1570.997 1.6 732164.2 284.8225 21992.58

ln_Il6 | 44 7.767747 2.845673 7.344768 .4700036 13.50376 5.650722 9.982617

----------------+-------------------------------------------------------------------------

3 Il6Tn | 43 15851.18 38021.72 1060.07 13.48 162893.6 283.05 8110.44

ln_Il6 | 43 7.388583 2.283489 6.96609 2.601207 12.00085 5.645624 9.000907

----------------+-------------------------------------------------------------------------

6 Il6Tn | 39 4986.701 10504.38 797.125 10.09 53336 148.49 4523.78

ln_Il6 | 39 6.763159 2.062571 6.681012 2.311545 10.88437 5.000518 8.417103

----------------+-------------------------------------------------------------------------

24 Il6Tn | 39 687.8191 1541.489 100.71 1.6 7135.355 49.95 448.22

ln_Il6 | 39 4.78028 2.059777 4.612245 .4700036 8.872817 3.911022 6.105284

----------------+-------------------------------------------------------------------------

Total Il6Tn |165 21151.63 89498.07 710.54 1.6 732164.2 148.49 4831.725

ln_Il6 |165 6.725358 2.595047 6.566025 .4700036 13.50376 5.000518 8.482959

------------------------------------------------------------------------------------------

-> restricted = Restricted

time variable | N mean sd p50 min max p25 p75

---------------+--------------------------------------------------------------------------

0 Il6Tn | 46 50717.9 146743.2 933.705 15.57 739642.9 291.48 13104.78

ln_Il6 | 46 7.558025 2.673232 6.838717 2.745346 13.51392 5.674972 9.480732

---------------+--------------------------------------------------------------------------

3 Il6Tn | 42 13583.63 45556.83 287.765 1.6 207969.7 89.26 1814.67

ln_Il6 | 42 6.128983 2.480992 5.661972 .4700036 12.24515 4.491553 7.503659

---------------+--------------------------------------------------------------------------

6 Il6Tn | 42 12204.61 41464.17 328.105 21.97 235182.6 106.44 1662.655

ln_Il6 | 42 6.301935 2.295915 5.792376 3.089678 12.36812 4.667582 7.416171

---------------+--------------------------------------------------------------------------

24 Il6Tn | 45 555.2482 1124.001 91.67 1.6 5520.32 17 417.87

ln_Il6 | 45 4.589687 2.099269 4.518195 .4700036 8.616191 2.833213 6.035171

---------------+--------------------------------------------------------------------------

Total Il6Tn | 175 19663.52 82675.45 313.75 1.6 739642.9 88.1 1910.94

ln_Il6 | 175 6.150306 2.610447 5.748597 .4700036 13.51392 4.478473 7.555351

------------------------------------------------------------------------------------------

. xttobit ln_Il6 i.time##restricted, ll(ln(1.60001)) nolog

Random-effects tobit regression Number of obs = 340

Uncensored = 332

Limits: lower = ln(1.60001) Left-censored = 8

upper = +inf Right-censored = 0

Group variable: RecordID Number of groups = 95

Random effects u_i ~ Gaussian Obs per group:

min = 1

avg = 3.6

max = 4

Integration method: mvaghermite Integration pts. = 12

Wald chi2(7) = 240.32

Log likelihood = -685.45082 Prob > chi2 = 0.0000

---------------------------------------------------------------------------------

ln_Il6 | Coef. Std. Err. z P>|z| [95% Conf. Interval]

----------------+----------------------------------------------------------------

time |

3 | -.4156447 .2968079 -1.40 0.161 -.9973776 .1660881

6 | -.9819135 .3069118 -3.20 0.001 -1.58345 -.3803774

24 | -3.236873 .3102094 -10.43 0.000 -3.844872 -2.628874

|

restricted |

Restricted | -.1752756 .4972965 -0.35 0.724 -1.149959 .7994075

|

time#restricted |

3#Restricted | -.9147428 .4206197 -2.17 0.030 -1.739142 -.0903434

6#Restricted | -.2409219 .4266839 -0.56 0.572 -1.077207 .5953633

24#Restricted | .1432438 .4259769 0.34 0.737 -.6916555 .9781432

|

_cons | 7.721851 .356388 21.67 0.000 7.023344 8.420359

----------------+----------------------------------------------------------------

/sigma_u | 1.956632 .1637246 11.95 0.000 1.635737 2.277526

/sigma_e | 1.37246 .0632931 21.68 0.000 1.248407 1.496512

----------------+----------------------------------------------------------------

rho | .6702332 .0435402 .5811548 .7505639

---------------------------------------------------------------------------------

LR test of sigma_u=0: chibar2(01) = 174.19 Prob >= chibar2 = 0.000

. testparm time#restricted

( 1) [ln_Il6]0.time#1.restricted = 0

( 2) [ln_Il6]3.time#1.restricted = 0

( 3) [ln_Il6]6.time#1.restricted = 0

chi2( 3) = 7.18

Prob > chi2 = 0.0665

. xttobit ln_Il6 i.time##restricted, ll(ln(1.60001)) nolog ///

> vce(bootstrap, reps(500) seed(010967))

(running xttobit on estimation sample)

Random-effects tobit regression Number of obs = 340

Uncensored = 332

Limits: lower = ln(1.60001) Left-censored = 8

upper = +inf Right-censored = 0

Group variable: RecordID Number of groups = 95

Random effects u_i ~ Gaussian Obs per group:

min = 1

avg = 3.6

max = 4

Integration method: mvaghermite Integration pts. = 12

Wald chi2(7) = 164.37

Log likelihood = -685.45082 Prob > chi2 = 0.0000

(Replications based on 95 clusters in RecordID)

---------------------------------------------------------------------------------

| Observed Bootstrap Normal-based

ln_Il6 | Coef. Std. Err. z P>|z| [95% Conf. Interval]

----------------+----------------------------------------------------------------

time |

3 | -.4156447 .2424752 -1.71 0.086 -.8908874 .059598

6 | -.9819135 .2712193 -3.62 0.000 -1.513494 -.4503335

24 | -3.236873 .356955 -9.07 0.000 -3.936492 -2.537254

|

restricted |

Restricted | -.1752756 .5652944 -0.31 0.757 -1.283232 .932681

|

time#restricted |

3#Restricted | -.9147428 .3780377 -2.42 0.016 -1.655683 -.1738025

6#Restricted | -.2409219 .3383852 -0.71 0.476 -.9041447 .4223009

24#Restricted | .1432438 .5186389 0.28 0.782 -.8732697 1.159757

|

_cons | 7.721851 .4295659 17.98 0.000 6.879918 8.563785

----------------+----------------------------------------------------------------

/sigma_u | 1.956632 .1507204 12.98 0.000 1.661225 2.252038

/sigma_e | 1.37246 .1182809 11.60 0.000 1.140633 1.604286

----------------+----------------------------------------------------------------

rho | .6702332 .0517676 .5636813 .7644852

---------------------------------------------------------------------------------

LR test of sigma_u=0: chibar2(01) = 174.19 Prob >= chibar2 = 0.000

. testparm time#restricted

( 1) [ln_Il6]3.time#1.restricted = 0

( 2) [ln_Il6]6.time#1.restricted = 0

( 3) [ln_Il6]24.time#1.restricted = 0

chi2( 3) = 7.45

Prob > chi2 = 0.0589

Looking at the reduced sample of those with non missing Lactate (pscore not missing).

. xttobit ln_Il6 i.time##restricted if pscore~=., ll(ln(1.60001)) nolog

Random-effects tobit regression Number of obs = 324

Uncensored = 316

Limits: lower = ln(1.60001) Left-censored = 8

upper = +inf Right-censored = 0

Group variable: RecordID Number of groups = 90

Random effects u_i ~ Gaussian Obs per group:

min = 1

avg = 3.6

max = 4

Integration method: mvaghermite Integration pts. = 12

Wald chi2(7) = 215.76

Log likelihood = -657.91586 Prob > chi2 = 0.0000

---------------------------------------------------------------------------------

ln_Il6 | Coef. Std. Err. z P>|z| [95% Conf. Interval]

----------------+----------------------------------------------------------------

time |

3 | -.3612325 .3045978 -1.19 0.236 -.9582332 .2357682

6 | -.9222312 .3152451 -2.93 0.003 -1.5401 -.3043621

24 | -3.191859 .3195852 -9.99 0.000 -3.818235 -2.565484

|

restricted |

Restricted | -.083436 .5197212 -0.16 0.872 -1.102071 .9351988

|

time#restricted |

3#Restricted | -1.00432 .4371608 -2.30 0.022 -1.86114 -.1475008

6#Restricted | -.306718 .4415079 -0.69 0.487 -1.172058 .5586216

24#Restricted | .1612721 .4429098 0.36 0.716 -.7068152 1.029359

|

_cons | 7.672765 .3694846 20.77 0.000 6.948588 8.396941

----------------+----------------------------------------------------------------

/sigma_u | 2.001815 .1716447 11.66 0.000 1.665397 2.338232

/sigma_e | 1.391837 .0657876 21.16 0.000 1.262896 1.520779

----------------+----------------------------------------------------------------

rho | .6741156 .044332 .5832281 .7556738

---------------------------------------------------------------------------------

LR test of sigma_u=0: chibar2(01) = 167.89 Prob >= chibar2 = 0.000

. testparm time#restricted

( 1) [ln_Il6]0.time#1.restricted = 0

( 2) [ln_Il6]3.time#1.restricted = 0

( 3) [ln_Il6]6.time#1.restricted = 0

chi2( 3) = 8.02

Prob > chi2 = 0.0456

Comparing the model above to the model below illustrates that the propensity score is not doing anything other than excluding 5 patients. Even though this reduced sample produces a significant p value, it should not be used – these models are included only to illustrate that adjustment for characteristics that may vary between groups has no impact.

. xttobit ln_Il6 i.time##restricted pscore, ll(ln(1.60001)) nolog

Random-effects tobit regression Number of obs = 324

Uncensored = 316

Limits: lower = ln(1.60001) Left-censored = 8

upper = +inf Right-censored = 0

Group variable: RecordID Number of groups = 90

Random effects u_i ~ Gaussian Obs per group:

min = 1

avg = 3.6

max = 4

Integration method: mvaghermite Integration pts. = 12

Wald chi2(8) = 219.44

Log likelihood = -656.05906 Prob > chi2 = 0.0000

---------------------------------------------------------------------------------

ln_Il6 | Coef. Std. Err. z P>|z| [95% Conf. Interval]

----------------+----------------------------------------------------------------

time |

3 | -.3573806 .3045847 -1.17 0.241 -.9543555 .2395944

6 | -.9119885 .3152535 -2.89 0.004 -1.529874 -.294103

24 | -3.191966 .3195404 -9.99 0.000 -3.818254 -2.565678

|

restricted |

Restricted | -.2809418 .5216557 -0.54 0.590 -1.303368 .7414847

|

time#restricted |

3#Restricted | -1.000671 .4371087 -2.29 0.022 -1.857388 -.1439532

6#Restricted | -.3205587 .4415354 -0.73 0.468 -1.185952 .5448347

24#Restricted | .1624251 .4428568 0.37 0.714 -.7055583 1.030409

|

pscore | 3.932916 2.01999 1.95 0.052 -.0261919 7.892024

_cons | 5.764662 1.045452 5.51 0.000 3.715614 7.81371

----------------+----------------------------------------------------------------

/sigma_u | 1.954978 .1686666 11.59 0.000 1.624398 2.285559

/sigma_e | 1.391869 .065788 21.16 0.000 1.262927 1.520811

----------------+----------------------------------------------------------------

rho | .6636188 .0453152 .5710159 .7472173

---------------------------------------------------------------------------------

LR test of sigma_u=0: chibar2(01) = 161.66 Prob >= chibar2 = 0.000

. testparm time#restricted

( 1) [ln_Il6]3.time#1.restricted = 0

( 2) [ln_Il6]6.time#1.restricted = 0

( 3) [ln_Il6]24.time#1.restricted = 0

chi2( 3) = 7.98

Prob > chi2 = 0.0464

. xttobit ln_Il6 i.time i.restricted , ll(ln(1.60001)) nolog

Random-effects tobit regression Number of obs = 340

Uncensored = 332

Limits: lower = ln(1.60001) Left-censored = 8

upper = +inf Right-censored = 0

Group variable: RecordID Number of groups = 95

Random effects u_i ~ Gaussian Obs per group:

min = 1

avg = 3.6

max = 4

Integration method: mvaghermite Integration pts. = 12

Wald chi2(4) = 227.05

Log likelihood = -688.99641 Prob > chi2 = 0.0000

------------------------------------------------------------------------------

ln_Il6 | Coef. Std. Err. z P>|z| [95% Conf. Interval]

-------------+----------------------------------------------------------------

time |

3 | -.8658483 .2130672 -4.06 0.000 -1.283452 -.4482443

6 | -1.102218 .2160848 -5.10 0.000 -1.525736 -.6786995

24 | -3.150607 .2154793 -14.62 0.000 -3.572938 -2.728275

|

restricted |

Restricted | -.4225529 .4316401 -0.98 0.328 -1.268552 .4234461

_cons | 7.846294 .3343167 23.47 0.000 7.191046 8.501543

-------------+----------------------------------------------------------------

/sigma_u | 1.95836 .1645468 11.90 0.000 1.635854 2.280866

/sigma_e | 1.391157 .06418 21.68 0.000 1.265366 1.516947

-------------+----------------------------------------------------------------

rho | .6646182 .0440968 .5745503 .746088

------------------------------------------------------------------------------

LR test of sigma_u=0: chibar2(01) = 170.05 Prob >= chibar2 = 0.000

. xttobit ln_Il6 i.time i.restricted pscore, ll(ln(1.60001)) nolog

Random-effects tobit regression Number of obs = 324

Uncensored = 316

Limits: lower = ln(1.60001) Left-censored = 8

upper = +inf Right-censored = 0

Group variable: RecordID Number of groups = 90

Random effects u_i ~ Gaussian Obs per group:

min = 1

avg = 3.6

max = 4

Integration method: mvaghermite Integration pts. = 12

Wald chi2(5) = 204.99

Log likelihood = -659.99335 Prob > chi2 = 0.0000

------------------------------------------------------------------------------

ln_Il6 | Coef. Std. Err. z P>|z| [95% Conf. Interval]

-------------+----------------------------------------------------------------

time |

3 | -.8372254 .2218957 -3.77 0.000 -1.272133 -.4023179

6 | -1.069259 .2242203 -4.77 0.000 -1.508722 -.6297948

24 | -3.095064 .2248064 -13.77 0.000 -3.535676 -2.654452

|

restricted |

Restricted | -.5677709 .45492 -1.25 0.212 -1.459398 .3238559

pscore | 3.96018 2.024062 1.96 0.050 -.0069087 7.927269

_cons | 5.893703 1.039613 5.67 0.000 3.856099 7.931307

-------------+----------------------------------------------------------------

/sigma_u | 1.955417 .1695724 11.53 0.000 1.623061 2.287773

/sigma_e | 1.414378 .0668785 21.15 0.000 1.283299 1.545458

-------------+----------------------------------------------------------------

rho | .6565205 .0460149 .5626914 .741564

------------------------------------------------------------------------------

LR test of sigma_u=0: chibar2(01) = 156.91 Prob >= chibar2 = 0.000

IL10 (Censored values)

N=170 (~50% of observations) below limit:

T0 n=43 below, T3 n=40 below, T6 n=24 below, T24 n=63 below

. bysort restricted:tabstat Il10Tn ln_Il10, ///

> s(n mean sd median min max p25 p75) by(time) col(stats) long

-> restricted = Standard

time variable | N mean sd p50 min max p25 p75

-----------------+--------------------------------------------------------------------------

0 Il10Tn | 42 254.1887 1386.005 3.535 .13 9010.99 .13 54.47

ln_Il10 | 42 1.100269 3.277735 1.258727 -2.040221 9.1062 -2.040221 3.99765

-----------------+--------------------------------------------------------------------------

3 Il10Tn | 43 159.3186 591.243 11.34 .13 3494.4 .13 67.67

ln_Il10 | 43 1.635797 3.141024 2.428336 -2.040221 8.158917 -2.040221 4.214643

-----------------+--------------------------------------------------------------------------

6 Il10Tn | 37 42.17135 64.50862 15.33 .13 247.97 .13 39.13

ln_Il10 | 37 1.858204 2.73171 2.729812 -2.040221 5.513308 -2.040221 3.666889

-----------------+--------------------------------------------------------------------------

24 Il10Tn | 38 2.979342 8.544538 .13 .13 44.35 .13 .13

ln_Il10 | 38 -1.188926 1.765425 -2.040221 -2.040221 3.792113 -2.040221 -2.040221

-----------------+--------------------------------------------------------------------------

Total Il10Tn | 160 120.0011 773.6342 3.525 .13 9010.99 .13 35.735

ln_Il10 | 160 .8757808 3.033853 1.25971 -2.040221 9.1062 -2.040221 3.576114

--------------------------------------------------------------------------------------------

-> restricted = Restricted

time variable | N mean sd p50 min max p25 p75

-----------------+--------------------------------------------------------------------------

0 Il10Tn | 45 265.3056 796.5031 .13 .13 4294.343 .13 62.04

ln_Il10 | 45 1.271466 3.631743 -2.040221 -2.040221 8.365054 -2.040221 4.127779

-----------------+--------------------------------------------------------------------------

3 Il10Tn | 41 131.5156 492.8781 .13 .13 3100.59 .13 38.43

ln_Il10 | 41 .5790645 3.354349 -2.040221 -2.040221 8.039348 -2.040221 3.648839

-----------------+--------------------------------------------------------------------------

6 Il10Tn | 39 97.28038 232.4902 7.87 .13 1308.79 .13 84.45

ln_Il10 | 39 1.776817 3.071732 2.063058 -2.040221 7.176858 -2.040221 4.43616

-----------------+--------------------------------------------------------------------------

24 Il10Tn | 44 12.06364 37.4236 .13 .13 195.32 .13 4.74

ln_Il10 | 44 -.7274724 2.361809 -2.040221 -2.040221 5.274639 -2.040221 .0975778

-----------------+--------------------------------------------------------------------------

Total Il10Tn | 169 128.1398 495.3522 .13 .13 4294.343 .13 38.43

ln_Il10 | 169 .6996728 3.254111 -2.040221 -2.040221 8.365054 -2.040221 3.648839

--------------------------------------------------------------------------------------------

. xttobit ln_Il10 i.time##restricted, ll(ln(0.130001)) nolog

Random-effects tobit regression Number of obs = 329

Uncensored = 159

Limits: lower = ln(0.130001) Left-censored = 170

upper = +inf Right-censored = 0

Group variable: RecordID Number of groups = 95

Random effects u_i ~ Gaussian Obs per group:

min = 1

avg = 3.5

max = 4

Integration method: mvaghermite Integration pts. = 12

Wald chi2(7) = 72.27

Log likelihood = -575.07846 Prob > chi2 = 0.0000

---------------------------------------------------------------------------------

ln_Il10 | Coef. Std. Err. z P>|z| [95% Conf. Interval]

----------------+----------------------------------------------------------------

time |

3 | .8938704 .8624168 1.04 0.300 -.7964355 2.584176

6 | 1.422752 .8993305 1.58 0.114 -.339903 3.185408

24 | -4.901041 1.043708 -4.70 0.000 -6.94667 -2.855411

|

restricted |

Restricted | -.1049736 1.208908 -0.09 0.931 -2.47439 2.264443

|

time#restricted |

3#Restricted | -2.093796 1.268206 -1.65 0.099 -4.579433 .3918415

6#Restricted | -.1505489 1.263189 -0.12 0.905 -2.626354 2.325256

24#Restricted | .5166278 1.427783 0.36 0.717 -2.281775 3.31503

|

_cons | -.4784288 .8710766 -0.55 0.583 -2.185708 1.22885

----------------+----------------------------------------------------------------

/sigma_u | 3.960545 .4652706 8.51 0.000 3.048632 4.872459

/sigma_e | 3.531778 .2580936 13.68 0.000 3.025924 4.037632

----------------+----------------------------------------------------------------

rho | .5570407 .066811 .4253751 .6826429

---------------------------------------------------------------------------------

LR test of sigma_u=0: chibar2(01) = 64.96 Prob >= chibar2 = 0.000

. testparm time#restricted

( 1) [ln_Il10]0.time#1.restricted = 0

( 2) [ln_Il10]3.time#1.restricted = 0

( 3) [ln_Il10]6.time#1.restricted = 0

chi2( 3) = 4.37

Prob > chi2 = 0.2243

xttobit ln_Il10 i.time##restricted pscore, ll(ln(0.130001)) nolog

Random-effects tobit regression Number of obs = 313

Uncensored = 155

Limits: lower = ln(0.130001) Left-censored = 158

upper = +inf Right-censored = 0

Group variable: RecordID Number of groups = 90

Random effects u_i ~ Gaussian Obs per group:

min = 1

avg = 3.5

max = 4

Integration method: mvaghermite Integration pts. = 12

Wald chi2(8) = 69.65

Log likelihood = -555.49225 Prob > chi2 = 0.0000

---------------------------------------------------------------------------------

ln_Il10 | Coef. Std. Err. z P>|z| [95% Conf. Interval]

----------------+----------------------------------------------------------------

time |

3 | .9067113 .8566692 1.06 0.290 -.7723294 2.585752

6 | 1.161136 .8976542 1.29 0.196 -.5982335 2.920506

24 | -4.862511 1.035862 -4.69 0.000 -6.892763 -2.832259

|

restricted |

Restricted | -.3721259 1.239191 -0.30 0.764 -2.800895 2.056643

|

time#restricted |

3#Restricted | -2.175386 1.277518 -1.70 0.089 -4.679276 .3285041

6#Restricted | -.0506406 1.268893 -0.04 0.968 -2.537624 2.436343

24#Restricted | .5713433 1.426918 0.40 0.689 -2.225365 3.368052

|

pscore | 5.962435 4.420293 1.35 0.177 -2.701179 14.62605

_cons | -3.19259 2.32901 -1.37 0.170 -7.757366 1.372186

----------------+----------------------------------------------------------------

/sigma_u | 3.907108 .4679572 8.35 0.000 2.989929 4.824287

/sigma_e | 3.482468 .2572349 13.54 0.000 2.978297 3.98664

----------------+----------------------------------------------------------------

rho | .5572756 .0681509 .4229917 .6852307

---------------------------------------------------------------------------------

LR test of sigma_u=0: chibar2(01) = 62.22 Prob >= chibar2 = 0.000

. testparm time#restricted

( 1) [ln_Il10]3.time#1.restricted = 0

( 2) [ln_Il10]6.time#1.restricted = 0

( 3) [ln_Il10]24.time#1.restricted = 0

chi2( 3) = 4.82

Prob > chi2 = 0.1857

. xttobit ln_Il10 i.time i.restricted , ll(ln(0.130001)) nolog

Random-effects tobit regression Number of obs = 329

Uncensored = 159

Limits: lower = ln(0.130001) Left-censored = 170

upper = +inf Right-censored = 0

Group variable: RecordID Number of groups = 95

Random effects u_i ~ Gaussian Obs per group:

min = 1

avg = 3.5

max = 4

Integration method: mvaghermite Integration pts. = 12

Wald chi2(4) = 67.75

Log likelihood = -577.27086 Prob > chi2 = 0.0000

------------------------------------------------------------------------------

ln_Il10 | Coef. Std. Err. z P>|z| [95% Conf. Interval]

-------------+----------------------------------------------------------------

time |

3 | -.0438755 .6362435 -0.07 0.945 -1.29089 1.203139

6 | 1.362382 .6420611 2.12 0.034 .1039652 2.620798

24 | -4.63016 .7318432 -6.33 0.000 -6.064546 -3.195773

|

restricted |

Restricted | -.6200112 .962195 -0.64 0.519 -2.505879 1.265856

_cons | -.2469124 .7899379 -0.31 0.755 -1.795162 1.301337

-------------+----------------------------------------------------------------

/sigma_u | 3.967113 .4675435 8.49 0.000 3.050745 4.883481

/sigma_e | 3.575525 .2611998 13.69 0.000 3.063583 4.087467

-------------+----------------------------------------------------------------

rho | .5517771 .0670971 .4198439 .6781735

------------------------------------------------------------------------------

LR test of sigma_u=0: chibar2(01) = 63.76 Prob >= chibar2 = 0.000

. xttobit ln_Il10 i.time i.restricted pscore, ll(ln(0.130001)) nolog

Random-effects tobit regression Number of obs = 313

Uncensored = 155

Limits: lower = ln(0.130001) Left-censored = 158

upper = +inf Right-censored = 0

Group variable: RecordID Number of groups = 90

Random effects u_i ~ Gaussian Obs per group:

min = 1

avg = 3.5

max = 4

Integration method: mvaghermite Integration pts. = 12

Wald chi2(5) = 64.57

Log likelihood = -557.90832 Prob > chi2 = 0.0000

------------------------------------------------------------------------------

ln_Il10 | Coef. Std. Err. z P>|z| [95% Conf. Interval]

-------------+----------------------------------------------------------------

time |

3 | -.0357759 .6405295 -0.06 0.955 -1.291191 1.219639

6 | 1.157761 .6455412 1.79 0.073 -.1074761 2.422999

24 | -4.561402 .7322422 -6.23 0.000 -5.99657 -3.126233

|

restricted |

Restricted | -.8754433 1.002582 -0.87 0.383 -2.840467 1.089581

pscore | 6.067617 4.434247 1.37 0.171 -2.623347 14.75858

_cons | -3.026954 2.302073 -1.31 0.189 -7.538934 1.485026

-------------+----------------------------------------------------------------

/sigma_u | 3.910285 .4703758 8.31 0.000 2.988365 4.832205

/sigma_e | 3.532893 .2608096 13.55 0.000 3.021716 4.044071

-------------+----------------------------------------------------------------

rho | .550573 .068538 .4159199 .679593

------------------------------------------------------------------------------

LR test of sigma_u=0: chibar2(01) = 60.82 Prob >= chibar2 = 0.000

# VEGFR1 (censored values)

T0: n=37 below n=2 above

T3: n=37 below n=3 above

T6: n=35 below n=2 above

T24: n=16 below n=3 above

bysort restricted:tabstat VEGFR1Tn ln_VEGFR1, ///

> s(n mean sd median min max p25 p75) by(time) col(stats) long

-> restricted = Standard

time variable | N mean sd p50 min max p25 p75

------------------+--------------------------------------------------------------------------

0 VEGFR1Tn | 43 2665.626 4945.756 509.4 125 24000 125 2555.7

ln_VEGFR1 | 43 6.506791 1.697263 6.233233 4.828314 10.08581 4.828314 7.846081

------------------+--------------------------------------------------------------------------

3 VEGFR1Tn | 44 2139.973 4232.228 636.45 125 24000 125 2195.25

ln_VEGFR1 | 44 6.418595 1.598537 6.455588 4.828314 10.08581 4.828314 7.69164

------------------+--------------------------------------------------------------------------

6 VEGFR1Tn | 38 2346.076 4515.03 349.05 125 24000 125 2562

ln_VEGFR1 | 38 6.384369 1.671106 5.852934 4.828314 10.08581 4.828314 7.848544

------------------+--------------------------------------------------------------------------

24 VEGFR1Tn | 36 3136.892 4979.815 1227.75 125 24000 662.85 3161.85

ln_VEGFR1 | 36 7.112987 1.43381 7.112461 4.828314 10.08581 6.482829 8.051241

------------------+--------------------------------------------------------------------------

Total VEGFR1Tn | 161 2551.924 4637.962 768 125 24000 125 2555.7

ln_VEGFR1 | 161 6.58934 1.618625 6.64379 4.828314 10.08581 4.828314 7.846081

---------------------------------------------------------------------------------------------

-> restricted = Restricted

time variable | N mean sd p50 min max p25 p75

------------------+--------------------------------------------------------------------------

0 VEGFR1Tn | 46 2222.872 4292.602 743.25 125 24000 125 2040

ln_VEGFR1 | 46 6.427778 1.627774 6.610825 4.828314 10.08581 4.828314 7.620705

------------------+--------------------------------------------------------------------------

3 VEGFR1Tn | 43 2385.225 5328.867 393.3 125 24000 125 1560.9

ln_VEGFR1 | 43 6.32881 1.615763 5.974573 4.828314 10.08581 4.828314 7.353018

------------------+--------------------------------------------------------------------------

6 VEGFR1Tn | 42 2205.236 4630.014 348.6 125 24000 125 1401.9

ln_VEGFR1 | 42 6.24723 1.629237 5.850642 4.828314 10.08581 4.828314 7.245584

------------------+--------------------------------------------------------------------------

24 VEGFR1Tn | 42 3287.805 5429.933 1057.2 125 24000 333 3093

ln_VEGFR1 | 42 7.015775 1.579097 6.961132 4.828314 10.08581 5.808143 8.036897

------------------+--------------------------------------------------------------------------

Total VEGFR1Tn | 173 2517.483 4908.102 661.2 125 24000 125 2040

ln_VEGFR1 | 173 6.502097 1.626998 6.494056 4.828314 10.08581 4.828314 7.620705

---------------------------------------------------------------------------------------------

. xttobit ln_VEGFR1 i.time##restricted, ll(ln(125.0001)) ul(ln(24000)) nolog

Random-effects tobit regression Number of obs = 334

Uncensored = 199

Limits: lower = ln(125.0001) Left-censored = 125

upper = ln(24000) Right-censored = 10

Group variable: RecordID Number of groups = 95

Random effects u_i ~ Gaussian Obs per group:

min = 1

avg = 3.5

max = 4

Integration method: mvaghermite Integration pts. = 12

Wald chi2(7) = 35.16

Log likelihood = -468.36873 Prob > chi2 = 0.0000

---------------------------------------------------------------------------------

ln_VEGFR1 | Coef. Std. Err. z P>|z| [95% Conf. Interval]

----------------+----------------------------------------------------------------

time |

3 | -.1825172 .2495306 -0.73 0.465 -.6715881 .3065537

6 | -.2837888 .2621451 -1.08 0.279 -.7975837 .2300062

24 | .788322 .2625259 3.00 0.003 .2737807 1.302863

|

restricted |

Restricted | -.1571335 .5126217 -0.31 0.759 -1.161854 .8475865

|

time#restricted |

3#Restricted | .0676248 .3551216 0.19 0.849 -.6284008 .7636503

6#Restricted | .0959273 .3647918 0.26 0.793 -.6190515 .8109062

24#Restricted | -.0861244 .360098 -0.24 0.811 -.7919036 .6196548

|

_cons | 6.035524 .3677093 16.41 0.000 5.314827 6.756221

----------------+----------------------------------------------------------------

/sigma_u | 2.136682 .2009523 10.63 0.000 1.742823 2.530541

/sigma_e | 1.014274 .064814 15.65 0.000 .8872405 1.141307

----------------+----------------------------------------------------------------

rho | .8161027 .0327108 .7452235 .8732086

---------------------------------------------------------------------------------

LR test of sigma_u=0: chibar2(01) = 227.96 Prob >= chibar2 = 0.000

. testparm time#restricted

( 1) [ln_VEGFR1]3.time#1.restricted = 0

( 2) [ln_VEGFR1]6.time#1.restricted = 0

( 3) [ln_VEGFR1]24.time#1.restricted = 0

chi2( 3) = 0.29

Prob > chi2 = 0.9619

. xttobit ln_VEGFR1 i.time##restricted pscore, ll(ln(125.0001)) ul(ln(24000)) nolog

Random-effects tobit regression Number of obs = 318

Uncensored = 189

Limits: lower = ln(125.0001) Left-censored = 119

upper = ln(24000) Right-censored = 10

Group variable: RecordID Number of groups = 90

Random effects u_i ~ Gaussian Obs per group:

min = 1

avg = 3.5

max = 4

Integration method: mvaghermite Integration pts. = 12

Wald chi2(8) = 38.14

Log likelihood = -445.03965 Prob > chi2 = 0.0000

---------------------------------------------------------------------------------

ln_VEGFR1 | Coef. Std. Err. z P>|z| [95% Conf. Interval]

----------------+----------------------------------------------------------------

time |

3 | -.1918793 .2555712 -0.75 0.453 -.6927897 .3090311

6 | -.2677674 .2687735 -1.00 0.319 -.7945538 .2590191

24 | .8142118 .2697804 3.02 0.003 .285452 1.342972

|

restricted |

Restricted | .1949324 .5322704 0.37 0.714 -.8482984 1.238163

|

time#restricted |

3#Restricted | .0709608 .3661461 0.19 0.846 -.6466723 .7885939

6#Restricted | -.0095293 .3749617 -0.03 0.980 -.7444407 .725382

24#Restricted | -.1660507 .3721275 -0.45 0.655 -.8954071 .5633057

|

pscore | -4.828723 2.161425 -2.23 0.025 -9.065038 -.5924091

_cons | 8.353672 1.10865 7.53 0.000 6.180758 10.52659

----------------+----------------------------------------------------------------

/sigma_u | 2.108426 .2036758 10.35 0.000 1.709229 2.507623

/sigma_e | 1.022127 .067001 15.26 0.000 .8908071 1.153446

----------------+----------------------------------------------------------------

rho | .8097078 .0344779 .7350095 .8698336

---------------------------------------------------------------------------------

LR test of sigma_u=0: chibar2(01) = 212.10 Prob >= chibar2 = 0.000

. testparm time#restricted

( 1) [ln_VEGFR1]3.time#1.restricted = 0

( 2) [ln_VEGFR1]6.time#1.restricted = 0

( 3) [ln_VEGFR1]24.time#1.restricted = 0

chi2( 3) = 0.42

Prob > chi2 = 0.9363

. xttobit ln_VEGFR1 i.time i.restricted , ll(ln(125.0001)) ul(ln(24000)) nolog

Random-effects tobit regression Number of obs = 334

Uncensored = 199

Limits: lower = ln(125.0001) Left-censored = 125

upper = ln(24000) Right-censored = 10

Group variable: RecordID Number of groups = 95

Random effects u_i ~ Gaussian Obs per group:

min = 1

avg = 3.5

max = 4

Integration method: mvaghermite Integration pts. = 12

Wald chi2(4) = 34.87

Log likelihood = -468.51375 Prob > chi2 = 0.0000

------------------------------------------------------------------------------

ln_VEGFR1 | Coef. Std. Err. z P>|z| [95% Conf. Interval]

-------------+----------------------------------------------------------------

time |

3 | -.1499173 .1775882 -0.84 0.399 -.4979838 .1981492

6 | -.2356528 .1823685 -1.29 0.196 -.5930886 .1217829

24 | .7419385 .1806271 4.11 0.000 .387916 1.095961

|

restricted |

Restricted | -.142662 .465776 -0.31 0.759 -1.055566 .7702421

_cons | 6.028751 .3509936 17.18 0.000 5.340816 6.716686

-------------+----------------------------------------------------------------

/sigma_u | 2.135929 .2009151 10.63 0.000 1.742142 2.529715

/sigma_e | 1.014865 .0648591 15.65 0.000 .8877438 1.141987

-------------+----------------------------------------------------------------

rho | .8158216 .0327593 .7448433 .8730154

------------------------------------------------------------------------------

LR test of sigma_u=0: chibar2(01) = 227.72 Prob >= chibar2 = 0.000

. xttobit ln_VEGFR1 i.time i.restricted pscore, ll(ln(125.0001)) ul(ln(24000)) nolog

Random-effects tobit regression Number of obs = 318

Uncensored = 189

Limits: lower = ln(125.0001) Left-censored = 119

upper = ln(24000) Right-censored = 10

Group variable: RecordID Number of groups = 90

Random effects u_i ~ Gaussian Obs per group:

min = 1

avg = 3.5

max = 4

Integration method: mvaghermite Integration pts. = 12

Wald chi2(5) = 37.73

Log likelihood = -445.24922 Prob > chi2 = 0.0000

------------------------------------------------------------------------------

ln_VEGFR1 | Coef. Std. Err. z P>|z| [95% Conf. Interval]

-------------+----------------------------------------------------------------

time |

3 | -.1597191 .1830611 -0.87 0.383 -.5185123 .1990741

6 | -.2746533 .1875231 -1.46 0.143 -.6421918 .0928851

24 | .7261781 .1867818 3.89 0.000 .3600926 1.092264

|

restricted |

Restricted | .1676923 .484952 0.35 0.729 -.7827961 1.118181

pscore | -4.828891 2.160789 -2.23 0.025 -9.06396 -.5938225

_cons | 8.367822 1.102933 7.59 0.000 6.206112 10.52953

-------------+----------------------------------------------------------------

/sigma_u | 2.107811 .2036622 10.35 0.000 1.708641 2.506982

/sigma_e | 1.022889 .0670663 15.25 0.000 .8914415 1.154337

-------------+----------------------------------------------------------------

rho | .8093879 .0345377 .7345667 .8696213

------------------------------------------------------------------------------

LR test of sigma_u=0: chibar2(01) = 211.75 Prob >= chibar2 = 0.000

# Heparan Sulfate (no censoring)

. bysort restricted:tabstat HeparanSulfateT ln_HepSulfate, ///

> s(n mean sd median min max p25 p75) by(time) col(stats) long f(%7.5g)

-> restricted = Standard

time Variable | N Mean SD p50 Min Max p25 p75

-------------------+-----------------------------------------------------------------------

0 HeparanSul~T | 44 2650.4 1687.4 2264.3 476.65 7108 1481.3 3361.9

ln_HepSulf~e | 44 7.691 .63789 7.725 6.1668 8.869 7.3005 8.1197

-------------------+-----------------------------------------------------------------------

3 HeparanSul~T | 44 2737.8 1651.4 2142.6 574.88 7019.2 1368.9 4093.2

ln_HepSulf~e | 44 7.7226 .64995 7.6691 6.3542 8.8564 7.2217 8.3164

-------------------+-----------------------------------------------------------------------

6 HeparanSul~T | 39 3028.2 2710.7 2064.8 435.6 15728 1381.6 4048

ln_HepSulf~e | 39 7.7406 .73747 7.6328 6.0767 9.6632 7.231 8.306

-------------------+-----------------------------------------------------------------------

24 HeparanSul~T | 39 2683.3 1608.8 2244 756.88 6971.2 1295.2 3644.8

ln_HepSulf~e | 39 7.7148 .61988 7.716 6.6292 8.8495 7.1664 8.2011

-------------------+-----------------------------------------------------------------------

Total HeparanSul~T |166 2770.1 1939.8 2219.5 435.6 15728 1384.8 3691.2

ln_HepSulf~e |166 7.7166 .6559 7.705 6.0767 9.6632 7.2333 8.2137

-------------------------------------------------------------------------------------------

-> restricted = Restricted

time Variable | N Mean SD p50 Min Max p25 p75

--------------------+------------------------------------------------------------------------

0 HeparanSul~T | 46 3123.8 2265 2248.2 524.64 9232 1601.5 3853.5

ln_HepSulf~e | 46 7.804 .71382 7.7178 6.2627 9.1304 7.3787 8.2567

--------------------+------------------------------------------------------------------------

3 HeparanSul~T | 44 2819.7 1790.7 2141.2 730 8544 1522.8 4048.4

ln_HepSulf~e | 44 7.7621 .61092 7.669 6.593 9.053 7.3279 8.3059

--------------------+------------------------------------------------------------------------

6 HeparanSul~T | 42 2994.8 2235.2 2118 482.88 7652.8 1138 4536.8

ln_HepSulf~e | 42 7.7124 .79856 7.6569 6.1798 8.9428 7.037 8.42

--------------------+------------------------------------------------------------------------

24 HeparanSul~T | 45 3020.1 1891.7 2624.5 657.5 8288 1505.6 4183.2

ln_HepSulf~e | 45 7.8231 .63506 7.8726 6.4884 9.0226 7.3169 8.3388

--------------------+------------------------------------------------------------------------

Total HeparanSul~T | 177 2991.2 2041 2264.8 482.88 9232 1457.6 4008

ln_HepSulf~e | 177 7.7767 .68748 7.7252 6.1798 9.1304 7.2845 8.296

---------------------------------------------------------------------------------------------

mixed ln_HepSulfate i.time##restricted ||RecordID:, mle

Mixed-effects ML regression Number of obs = 343

Group variable: RecordID Number of groups = 95

Obs per group:

min = 1

avg = 3.6

max = 4

Wald chi2(7) = 1.95

Log likelihood = -229.19329 Prob > chi2 = 0.9623

---------------------------------------------------------------------------------

ln_HepSulfate | Coefficient Std. err. z P>|z| [95% conf. interval]

----------------+----------------------------------------------------------------

time |

3 | .0295097 .0724818 0.41 0.684 -.1125521 .1715714

6 | .0266738 .0756145 0.35 0.724 -.1215279 .1748755

24 | -.0003066 .0760922 -0.00 0.997 -.1494446 .1488314

|

restricted |

Restricted | .1149708 .1378878 0.83 0.404 -.1552842 .3852259

|

time#restricted |

3#Restricted | -.0780619 .1024271 -0.76 0.446 -.2788154 .1226916

6#Restricted | -.114078 .1052161 -1.08 0.278 -.3202977 .0921416

24#Restricted | -.0386052 .1046664 -0.37 0.712 -.2437474 .1665371

|

_cons | 7.690974 .0988447 77.81 0.000 7.497242 7.884706

---------------------------------------------------------------------------------

------------------------------------------------------------------------------

Random-effects parameters | Estimate Std. err. [95% conf. interval]

-----------------------------+------------------------------------------------

RecordID: Identity |

var(_cons) | .3268991 .052206 .2390431 .4470451

-----------------------------+------------------------------------------------

var(Residual) | .1146247 .0102765 .0961536 .1366442

------------------------------------------------------------------------------

LR test vs. linear model: chibar2(01) = 239.89 Prob >= chibar2 = 0.0000

. testparm time#restricted

( 1) [ln_HepSulfate]3.time#1.restricted = 0

( 2) [ln_HepSulfate]6.time#1.restricted = 0

( 3) [ln_HepSulfate]24.time#1.restricted = 0

chi2( 3) = 1.32

Prob > chi2 = 0.7247

No significant interaction between time and group detected (p=0.72).

The next model is the same as the one above but on the sample with complete propensity score data. This is included because in the model after this one, the pscore variable was significant. The model below illustrates the effect of the loss of n=5 patients before we add the propensity score variable.

. mixed ln_HepSulfate i.time##restricted if pscore~=. ||RecordID:, mle

Mixed-effects ML regression Number of obs = 327

Group variable: RecordID Number of groups = 90

Obs per group:

min = 1

avg = 3.6

max = 4

Wald chi2(7) = 1.92

Log likelihood = -219.59974 Prob > chi2 = 0.9639

---------------------------------------------------------------------------------

ln_HepSulfate | Coefficient Std. err. z P>|z| [95% conf. interval]

----------------+----------------------------------------------------------------

time |

3 | .0389303 .073562 0.53 0.597 -.1052485 .1831092

6 | .0261229 .0768244 0.34 0.734 -.1244502 .1766959

24 | .0027663 .0774777 0.04 0.972 -.1490872 .1546198

|

restricted |

Restricted | .1041874 .1421898 0.73 0.464 -.1744995 .3828743

|

time#restricted |

3#Restricted | -.0855969 .1052184 -0.81 0.416 -.2918211 .1206273

6#Restricted | -.1161365 .1076928 -1.08 0.281 -.3272105 .0949375

24#Restricted | -.0560791 .1076016 -0.52 0.602 -.2669744 .1548162

|

_cons | 7.712032 .1011746 76.22 0.000 7.513734 7.910331

---------------------------------------------------------------------------------

------------------------------------------------------------------------------

Random-effects parameters | Estimate Std. err. [95% conf. interval]

-----------------------------+------------------------------------------------

RecordID: Identity |

var(_cons) | .3315503 .0542739 .2405525 .4569713

-----------------------------+------------------------------------------------

var(Residual) | .1153598 .0105803 .0963798 .1380775

------------------------------------------------------------------------------

LR test vs. linear model: chibar2(01) = 230.61 Prob >= chibar2 = 0.0000

. testparm time#restricted

( 1) [ln_HepSulfate]3.time#1.restricted = 0

( 2) [ln_HepSulfate]6.time#1.restricted = 0

( 3) [ln_HepSulfate]24.time#1.restricted = 0

chi2( 3) = 1.28

Prob > chi2 = 0.7332

The interaction of group and time is still not significant (p=0.73) and the p value is of similar magnitude (a little bigger which is not surprising given the sample is slightly reduced).

. mixed ln_HepSulfate i.time##restricted pscore ||RecordID:, mle

Performing EM optimization ...

Mixed-effects ML regression Number of obs = 327

Group variable: RecordID Number of groups = 90

Obs per group:

min = 1

avg = 3.6

max = 4

Wald chi2(8) = 13.65

Log likelihood = -214.10266 Prob > chi2 = 0.0913

---------------------------------------------------------------------------------

ln_HepSulfate | Coefficient Std. err. z P>|z| [95% conf. interval]

----------------+----------------------------------------------------------------

time |

3 | .040274 .0735891 0.55 0.584 -.1039581 .184506

6 | .0306033 .0768435 0.40 0.690 -.1200071 .1812138

24 | .0029329 .0774878 0.04 0.970 -.1489405 .1548062

|

restricted |

Restricted | .0100705 .138112 0.07 0.942 -.2606242 .2807651

|

time#restricted |

3#Restricted | -.0849953 .1052435 -0.81 0.419 -.2912688 .1212782

6#Restricted | -.1217269 .1077217 -1.13 0.258 -.3328576 .0894037

24#Restricted | -.0549571 .1076139 -0.51 0.610 -.2658764 .1559623

|

pscore | 1.874252 .5472901 3.42 0.001 .8015829 2.946921

_cons | 6.802451 .2824616 24.08 0.000 6.248837 7.356066

---------------------------------------------------------------------------------

------------------------------------------------------------------------------

Random-effects parameters | Estimate Std. err. [95% conf. interval]

-----------------------------+------------------------------------------------

RecordID: Identity |

var(_cons) | .2887943 .0480512 .2084304 .4001441

-----------------------------+------------------------------------------------

var(Residual) | .1154603 .0105984 .0964492 .1382187

------------------------------------------------------------------------------

LR test vs. linear model: chibar2(01) = 203.78 Prob >= chibar2 = 0.0000

. testparm time#restricted

( 1) [ln_HepSulfate]3.time#1.restricted = 0

( 2) [ln_HepSulfate]6.time#1.restricted = 0

( 3) [ln_HepSulfate]24.time#1.restricted = 0

chi2( 3) = 1.38

Prob > chi2 = 0.7099

Inclusion of the propensity score has not changed the conclusions from the unadjusted model.

. mixed ln_HepSulfate i.time i.restricted ||RecordID:, mle

Mixed-effects ML regression Number of obs = 343

Group variable: RecordID Number of groups = 95

Obs per group:

min = 1

avg = 3.6

max = 4

Wald chi2(4) = 0.63

Log likelihood = -229.85122 Prob > chi2 = 0.9593

-------------------------------------------------------------------------------

ln_HepSulfate | Coefficient Std. err. z P>|z| [95% conf. interval]

--------------+----------------------------------------------------------------

time |

3 | -.0098581 .0513342 -0.19 0.848 -.1104713 .0907551

6 | -.0320035 .0527024 -0.61 0.544 -.1352982 .0712913

24 | -.0198716 .0523604 -0.38 0.704 -.122496 .0827529

|

restricted |

Restricted | .059383 .1234141 0.48 0.630 -.1825043 .3012702

_cons | 7.71929 .0937863 82.31 0.000 7.535472 7.903108

-------------------------------------------------------------------------------

------------------------------------------------------------------------------

Random-effects parameters | Estimate Std. err. [95% conf. interval]

-----------------------------+------------------------------------------------

RecordID: Identity |

var(_cons) | .327189 .0522702 .2392296 .4474893

-----------------------------+------------------------------------------------

var(Residual) | .115179 .010326 .0966188 .1373047

------------------------------------------------------------------------------

LR test vs. linear model: chibar2(01) = 239.20 Prob >= chibar2 = 0.0000

. mixed ln_HepSulfate i.time i.restricted pscore ||RecordID:, mle

Mixed-effects ML regression Number of obs = 327

Group variable: RecordID Number of groups = 90

Obs per group:

min = 1

avg = 3.6

max = 4

Wald chi2(5) = 12.25

Log likelihood = -214.79172 Prob > chi2 = 0.0315

-------------------------------------------------------------------------------

ln_HepSulfate | Coefficient Std. err. z P>|z| [95% conf. interval]

--------------+----------------------------------------------------------------

time |

3 | -.0016368 .0527423 -0.03 0.975 -.1050098 .1017362

6 | -.0310831 .0539809 -0.58 0.565 -.1368837 .0747174

24 | -.0246426 .0538924 -0.46 0.647 -.1302698 .0809846

|

restricted |

Restricted | -.0529104 .1230273 -0.43 0.667 -.2940394 .1882186

pscore | 1.86661 .5476935 3.41 0.001 .7931507 2.94007

_cons | 6.837774 .2808052 24.35 0.000 6.287406 7.388142

-------------------------------------------------------------------------------

------------------------------------------------------------------------------

Random-effects parameters | Estimate Std. err. [95% conf. interval]

-----------------------------+------------------------------------------------

RecordID: Identity |

var(_cons) | .2892064 .0481339 .2087077 .4007534

-----------------------------+------------------------------------------------

var(Residual) | .1160538 .0106525 .0969456 .1389283

------------------------------------------------------------------------------

LR test vs. linear model: chibar2(01) = 203.27 Prob >= chibar2 = 0.0000

# Syndecan 4 (censored values)

. bysort restricted:tabstat Syn4 ln_Syn4, ///

> s(n mean sd median min max p25 p75) by(time) col(stats) long f(%7.4g)

-> restricted = Standard

time Variable | N Mean SD p50 Min Max p25 p75

----------------+-------------------------------------------------------------------------

0 Syn4 | 44 2853 5595 804.5 113.8 30720 510 2136

ln_Syn4 | 44 7.032 1.26 6.69 4.735 10.33 6.234 7.666

----------------+-------------------------------------------------------------------------

3 Syn4 | 44 3439 6410 1195 259.9 30780 502.1 2388

ln_Syn4 | 44 7.203 1.229 7.085 5.56 10.33 6.219 7.776

----------------+-------------------------------------------------------------------------

6 Syn4 | 39 2407 4030 756.9 113.8 16010 411.7 1628

ln_Syn4 | 39 6.909 1.212 6.629 4.735 9.681 6.02 7.395

----------------+-------------------------------------------------------------------------

24 Syn4 | 39 2144 4462 542.1 113.8 24000 282.8 1281

ln_Syn4 | 39 6.572 1.34 6.295 4.735 10.09 5.645 7.155

----------------+-------------------------------------------------------------------------

Total Syn4 |166 2737 5238 772.1 113.8 30780 435.2 1872

ln_Syn4 |166 6.94 1.27 6.649 4.735 10.33 6.076 7.535

------------------------------------------------------------------------------------------

-> restricted = Restricted

time Variable | N Mean SD p50 Min Max p25 p75

----------------+--------------------------------------------------------------------------

0 Syn4 | 46 3189 7021 868.2 113.8 39325 483.6 2156

ln_Syn4 | 46 7.034 1.301 6.766 4.735 10.58 6.181 7.676

----------------+--------------------------------------------------------------------------

3 Syn4 | 44 3773 11950 826 113.8 78250 449.4 1918

ln_Syn4 | 44 6.961 1.33 6.716 4.735 11.27 6.106 7.557

----------------+--------------------------------------------------------------------------

6 Syn4 | 42 2494 5723 602.1 113.8 29000 361.1 2134

ln_Syn4 | 42 6.752 1.304 6.399 4.735 10.28 5.889 7.666

----------------+--------------------------------------------------------------------------

24 Syn4 | 45 3301 8633 776.1 113.8 52150 324.2 1714

ln_Syn4 | 45 6.837 1.388 6.654 4.735 10.86 5.781 7.447

----------------+--------------------------------------------------------------------------

Total Syn4 | 177 3197 8600 798 113.8 78250 388.4 1885

ln_Syn4 | 177 6.899 1.325 6.682 4.735 11.27 5.962 7.542

-------------------------------------------------------------------------------------------

. xttobit ln_Syn4 i.time##restricted, ll(ln(113.86)) nolog

Random-effects tobit regression Number of obs = 343

Uncensored = 328

Limits: Lower = ln(113.86) Left-censored = 15

Upper = +inf Right-censored = 0

Group variable: RecordID Number of groups = 95

Random effects u_i ~ Gaussian Obs per group:

min = 1

avg = 3.6

max = 4

Integration method: mvaghermite Integration pts. = 12

Wald chi2(7) = 38.04

Log likelihood = -405.05226 Prob > chi2 = 0.0000

---------------------------------------------------------------------------------

ln_Syn4 | Coefficient Std. err. z P>|z| [95% conf. interval]

----------------+----------------------------------------------------------------

time |

3 | .1575458 .1115512 1.41 0.158 -.0610906 .3761822

6 | -.1836505 .1167338 -1.57 0.116 -.4124446 .0451435

24 | -.4581449 .1182957 -3.87 0.000 -.6900003 -.2262895

|

restricted |

Restricted | .0307179 .2728157 0.11 0.910 -.5039911 .565427

|

time#restricted |

3#Restricted | -.2902201 .1583524 -1.83 0.067 -.600585 .0201449

6#Restricted | -.079336 .1630564 -0.49 0.627 -.3989207 .2402487

24#Restricted | .1722969 .1625629 1.06 0.289 -.1463205 .4909144

|

_cons | 7.012577 .1955473 35.86 0.000 6.629311 7.395842

----------------+----------------------------------------------------------------

/sigma_u | 1.211318 .0935654 12.95 0.000 1.027934 1.394703

/sigma_e | .5200429 .0240994 21.58 0.000 .472809 .5672768

----------------+----------------------------------------------------------------

rho | .8443696 .0238699 .7929702 .8865514

---------------------------------------------------------------------------------

LR test of sigma_u=0: chibar2(01) = 346.83 Prob >= chibar2 = 0.000

. testparm time#restricted

( 1) [ln_Syn4]3.time#1.restricted = 0

( 2) [ln_Syn4]6.time#1.restricted = 0

( 3) [ln_Syn4]24.time#1.restricted = 0

chi2( 3) = 8.28

Prob > chi2 = 0.0405

Residuals a little skewed so have bootstrapped:

. xttobit ln_Syn4 i.time##restricted, ll(ln(113.86)) nolog ///

> vce(bootstrap, reps(500) seed(010967))

(running xttobit on estimation sample)

Random-effects tobit regression Number of obs = 343

Uncensored = 328

Limits: Lower = ln(113.86) Left-censored = 15

Upper = +inf Right-censored = 0

Replications = 500

Group variable: RecordID Number of groups = 95

Random effects u_i ~ Gaussian Obs per group:

min = 1

avg = 3.6

max = 4

Integration method: mvaghermite Integration pts. = 12

Wald chi2(7) = 26.78

Log likelihood = -405.05226 Prob > chi2 = 0.0004

(Replications based on 95 clusters in RecordID)

---------------------------------------------------------------------------------

| Observed Bootstrap Normal-based

ln_Syn4 | coefficient std. err. z P>|z| [95% conf. interval]

----------------+----------------------------------------------------------------

time |

3 | .1575458 .0931941 1.69 0.091 -.0251113 .3402029

6 | -.1836505 .0970846 -1.89 0.059 -.3739328 .0066317

24 | -.4581449 .1558037 -2.94 0.003 -.7635145 -.1527753

|

restricted |

Restricted | .0307179 .2724896 0.11 0.910 -.5033518 .5647877

|

time#restricted |

3#Restricted | -.2902201 .1330865 -2.18 0.029 -.5510649 -.0293753

6#Restricted | -.079336 .1744528 -0.45 0.649 -.4212572 .2625852

24#Restricted | .1722969 .2012213 0.86 0.392 -.2220895 .5666833

|

_cons | 7.012577 .1851127 37.88 0.000 6.649762 7.375391

----------------+----------------------------------------------------------------

/sigma_u | 1.211318 .1166645 10.38 0.000 .9826603 1.439977

/sigma_e | .5200429 .0363995 14.29 0.000 .4487013 .5913845

----------------+----------------------------------------------------------------

rho | .8443696 .0339179 .7686307 .9016162

---------------------------------------------------------------------------------

LR test of sigma_u=0: chibar2(01) = 346.83 Prob >= chibar2 = 0.000

.

. testparm time#restricted

( 1) [ln_Syn4]3.time#1.restricted = 0

( 2) [ln_Syn4]6.time#1.restricted = 0

( 3) [ln_Syn4]24.time#1.restricted = 0

chi2( 3) = 10.87

Prob > chi2 = 0.0125

The model below just swaps the reference group to T24 (chosen because the difference between the coefficients for T3 and T24 is the largest) which illustrates that the difference between T3 and T24 is also significantly different between groups.

. xttobit ln_Syn4 b24.time##b1.restricted, ll(ln(113.86)) nolog ///

> vce(bootstrap, reps(500) seed(010967))

(running xttobit on estimation sample)

Random-effects tobit regression Number of obs = 343

Uncensored = 328

Limits: Lower = ln(113.86) Left-censored = 15

Upper = +inf Right-censored = 0

Replications = 500

Group variable: RecordID Number of groups = 95

Random effects u_i ~ Gaussian Obs per group:

min = 1

avg = 3.6

max = 4

Integration method: mvaghermite Integration pts. = 12

Wald chi2(7) = 26.78

Log likelihood = -405.05226 Prob > chi2 = 0.0004

(Replications based on 95 clusters in RecordID)

---------------------------------------------------------------------------------

| Observed Bootstrap Normal-based

ln_Syn4 | coefficient std. err. z P>|z| [95% conf. interval]

----------------+----------------------------------------------------------------

time |

0 | .285848 .1251752 2.28 0.022 .040509 .5311869

3 | .1531737 .1041052 1.47 0.141 -.0508686 .3572161

6 | .0228614 .1296245 0.18 0.860 -.2311979 .2769208

|

restricted |

Standard | -.2030148 .2896151 -0.70 0.483 -.7706499 .3646202

|

time#restricted |

0#Standard | .1722969 .2012213 0.86 0.392 -.2220895 .5666833

3#Standard | .462517 .1707834 2.71 0.007 .1277878 .7972462

6#Standard | .2516329 .1740342 1.45 0.148 -.0894679 .5927337

|

_cons | 6.757447 .2013934 33.55 0.000 6.362723 7.15217

----------------+----------------------------------------------------------------

/sigma_u | 1.211318 .1166645 10.38 0.000 .9826603 1.439977

/sigma_e | .5200429 .0363995 14.29 0.000 .4487013 .5913845

----------------+----------------------------------------------------------------

rho | .8443696 .0339179 .7686307 .9016162

---------------------------------------------------------------------------------

LR test of sigma_u=0: chibar2(01) = 346.83 Prob >= chibar2 = 0.000

These results indicate that the two groups do not differ significantly at T0 or T24 but in change from T0 to T3 or T3 to T24 differ between the two groups.

Running the same analysis using the sample for which the propensity score is available (but not including the propensity score) :

. xttobit ln_Syn4 i.time##restricted if pscore~=., ll(ln(113.86)) nolog ///

> vce(bootstrap, reps(500) seed(010967))

(running xttobit on estimation sample)

Random-effects tobit regression Number of obs = 327

Uncensored = 312

Limits: Lower = ln(113.86) Left-censored = 15

Upper = +inf Right-censored = 0

Replications = 500

Group variable: RecordID Number of groups = 90

Random effects u_i ~ Gaussian Obs per group:

min = 1

avg = 3.6

max = 4

Integration method: mvaghermite Integration pts. = 12

Wald chi2(7) = 28.45

Log likelihood = -385.42247 Prob > chi2 = 0.0002

(Replications based on 90 clusters in RecordID)

---------------------------------------------------------------------------------

| Observed Bootstrap Normal-based

ln_Syn4 | coefficient std. err. z P>|z| [95% conf. interval]

----------------+----------------------------------------------------------------

time |

3 | .1481744 .0913151 1.62 0.105 -.0307998 .3271487

6 | -.1901112 .0958544 -1.98 0.047 -.3779823 -.0022402

24 | -.4809138 .1523536 -3.16 0.002 -.7795214 -.1823061

|

restricted |

Restricted | .0164356 .2666881 0.06 0.951 -.5062635 .5391347

|

time#restricted |

3#Restricted | -.2478275 .1345608 -1.84 0.066 -.5115619 .0159069

6#Restricted | -.0767018 .1583969 -0.48 0.628 -.3871541 .2337505

24#Restricted | .2104599 .1993733 1.06 0.291 -.1803045 .6012243

|

_cons | 7.028807 .1988937 35.34 0.000 6.638982 7.418631

----------------+----------------------------------------------------------------

/sigma_u | 1.241883 .1162347 10.68 0.000 1.014068 1.469699

/sigma_e | .5145952 .0384321 13.39 0.000 .4392697 .5899208

----------------+----------------------------------------------------------------

rho | .8534609 .0306653 .7851074 .9054467

---------------------------------------------------------------------------------

LR test of sigma_u=0: chibar2(01) = 343.13 Prob >= chibar2 = 0.000

. testparm time#restricted

( 1) [ln_Syn4]3.time#1.restricted = 0

( 2) [ln_Syn4]6.time#1.restricted = 0

( 3) [ln_Syn4]24.time#1.restricted = 0

chi2( 3) = 7.80

Prob > chi2 = 0.0503

Adjusting for propensity score:

. xttobit ln_Syn4 i.time##restricted pscore, ll(ln(113.86)) nolog ///

> vce(bootstrap, reps(500) seed(010967))

(running xttobit on estimation sample)

Random-effects tobit regression Number of obs = 327

Uncensored = 312

Limits: Lower = ln(113.86) Left-censored = 15

Upper = +inf Right-censored = 0

Replications = 500

Group variable: RecordID Number of groups = 90

Random effects u_i ~ Gaussian Obs per group:

min = 1

avg = 3.6

max = 4

Integration method: mvaghermite Integration pts. = 12

Wald chi2(8) = 29.51

Log likelihood = -384.60977 Prob > chi2 = 0.0003

(Replications based on 90 clusters in RecordID)

---------------------------------------------------------------------------------

| Observed Bootstrap Normal-based

ln_Syn4 | coefficient std. err. z P>|z| [95% conf. interval]

----------------+----------------------------------------------------------------

time |

3 | .1476291 .0912759 1.62 0.106 -.0312685 .3265266

6 | -.1915386 .0958194 -2.00 0.046 -.3793412 -.0037359

24 | -.4805029 .152289 -3.16 0.002 -.7789838 -.182022

|

restricted |

Restricted | .0953196 .2826329 0.34 0.736 -.4586307 .6492699

|

time#restricted |

3#Restricted | -.2478558 .1345866 -1.84 0.066 -.5116407 .015929

6#Restricted | -.0747924 .1581544 -0.47 0.636 -.3847693 .2351846

24#Restricted | .2101319 .1991845 1.05 0.291 -.1802625 .6005263

|

pscore | -1.557384 1.45092 -1.07 0.283 -4.401135 1.286367

_cons | 7.784444 .7830686 9.94 0.000 6.249657 9.31923

----------------+----------------------------------------------------------------

/sigma_u | 1.230157 .1112836 11.05 0.000 1.012045 1.448269

/sigma_e | .5144177 .038422 13.39 0.000 .439112 .5897234

----------------+----------------------------------------------------------------

rho | .8511593 .0299305 .7848014 .9022234

---------------------------------------------------------------------------------

LR test of sigma_u=0: chibar2(01) = 341.62 Prob >= chibar2 = 0.000

. testparm time#restricted

( 1) [ln_Syn4]3.time#1.restricted = 0

( 2) [ln_Syn4]6.time#1.restricted = 0

( 3) [ln_Syn4]24.time#1.restricted = 0

chi2( 3) = 7.80

Prob > chi2 = 0.0504

In the sample with a propensity score, the significance of the interaction term is lost (p=0.05) due to the loss of sample (power). Including the propensity has no impact on the significance of the interaction term (0.05) and shows no evidence that the propensity score is necessary in the model (p=0.28).
